# TLR4 aggravates microglial pyroptosis by promoting DDX3X‐mediated NLRP3 inflammasome activation via JAK2/STAT1 pathway after spinal cord injury

**DOI:** 10.1002/ctm2.894

**Published:** 2022-06-12

**Authors:** Jin Wang, Fan Zhang, Haocheng Xu, Haiyuan Yang, Minghao Shao, Shun Xu, Feizhou Lyu

**Affiliations:** ^1^ Department of Orthopedics Huashan Hospital Fudan University Shanghai P. R. China; ^2^ Department of Orthopedics Shanghai Fifth People's Hospital Fudan University Shanghai P. R. China

**Keywords:** DDX3X, JAK2/STAT1 pathway, NLRP3 inflammasome, pyroptosis, spinal cord injury

## Abstract

**Background:**

Toll‐like receptor 4 (TLR4) participates in the initiation of neuroinflammation in various neurological diseases, including central nervous system injuries. NLR family pyrin domain containing 3 (NLRP3) inflammasome‐mediated microglial pyroptosis is crucial for the inflammatory response during secondary spinal cord injury (SCI). However, the underlying mechanism by which TLR4 regulates NLRP3 inflammasome activation and microglial pyroptosis after SCI remains uncertain.

**Methods:**

We established an in vivo mouse model of SCI using TLR4‐knockout (TLR4‐KO) and wild‐type (WT) mice. The levels of pyroptosis, tissue damage and neurological function recovery were evaluated in the three groups (Sham, SCI, SCI‐TLR4‐KO). To identify differentially expressed proteins, tandem mass tag (TMT)‐based proteomics was conducted using spinal cord tissue between TLR4‐KO and WT mice after SCI. For our in vitro model, mouse microglial BV2 cells were exposed to lipopolysaccharides (1 µg/ml, 8 h) and adenosine triphosphate (ATP) (5 mM, 2 h) to induce pyroptosis. A series of molecular biological experiments, including Western blot (WB), real‐time quantitative polymerase chain reaction (RT‐qPCR), enzyme‐linked immunosorbent assay (ELISA), immunofluorescence (IF), immunohistochemical (IHC), chromatin immunoprecipitation (ChIP), Dual‐Luciferase Reporter assay (DLA) and co‐immunoprecipitation (Co‐IP), were performed to explore the specific mechanism of microglial pyroptosis in vivo and in vitro.

**Results:**

Our results indicated that TLR4 promoted the expression of dead‐box helicase 3 X‐linked (DDX3X), which mediated NLRP3 inflammasome activation and microglial pyroptosis after SCI. Further analysis revealed that TLR4 upregulated the DDX3X/NLRP3 axis by activating the JAK2/STAT1 signalling pathway, and importantly, STAT1 was identified as a transcription factor promoting *DDX3X* expression. In addition, we found that biglycan was increased after SCI and interacted with TLR4 to jointly regulate microglial pyroptosis through the JAK2/STAT1/DDX3X/NLRP3 axis after SCI.

**Conclusion:**

Our study preliminarily identified a novel mechanism by which TLR4 regulates NLRP3 inflammasome‐mediated microglial pyroptosis in response to SCI—providing a novel and promising therapeutic target for SCI.

## Graphical abstract

1. TLR4 and DDX3X are upregulated after SCI in mice.
2. TLR4 deficiency alleviated DDX3X‐dependent NLRP3 inflammasome activation and microglial pyroptosis after SCI in mice.
3. TLR4 interacts with BGN and jointly promotes STAT1‐dependent transcriptional activation of DDX3X by activating the JAK2/STAT1 pathway after SCI in mice.

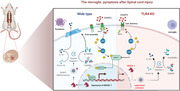

## 1 INTRODUCTION

Spinal cord injury (SCI), as one of the most severe central nervous system (CNS) injuries, usually leads to motor and sensory dysfunction, which greatly impairs the quality of life of patients. Although various strategies have been developed (e.g., steroid hormones, neurotrophic drugs, hyperbaric oxygen therapy and functional electrical stimulation),^1^, ^2^, ^3^, ^4^, ^5^ none is satisfactory for treating SCI in the clinic. In general, SCI is divided into two stages (primary and secondary injuries).^6^, ^7^ Different from primary SCI, which is characterised by destruction of the spinal cord structure, secondary SCI is induced by a series of pathophysiological responses (including hypoxia, ischaemia, oxidative stress, neuroinflammation, cell apoptosis and cell death) following primary injury and is relatively remediable.^8^, ^9^, ^10^ Recently, neuroinflammation has attracted increasing attention for its essential role in secondary SCI. Moreover, extensive evidence indicates that microglial pyroptosis is a significant cause of neuroinflammation after SCI.^11^, ^12^ Therefore, inhibiting microglial pyroptosis may be one of the promising and effective therapeutic strategies for alleviating neuroinflammation after SCI.

Pyroptosis, a novel programmed inflammatory cell death characterised by the formation of transmembrane pores, swelling of cells and rupture, and release of many inflammatory cytokines and other cytoplasmic contents, is reportedly caused by the activation of inflammasomes.^13^ Notably, NLR family pyrin domain containing 3 (NLRP3) inflammasomes comprising ASC (associated speck‐like protein containing a caspase recruitment domain), pro‐caspase‐1 and NLRP3, are one of the most extensively studied inflammasomes in CNS injuries, including traumatic brain injury (TBI) and SCI.^14^, ^15^ Although pyroptosis mediated by the NLRP3 inflammasome has been reported in a variety of diseases, its regulatory mechanism in SCI remains to be elucidated.

Toll‐like receptor (TLR4), a crucial innate immune receptor, is known to initiate an inflammatory response to various diseases, such as acute lung injury, sepsis, inflammatory bowel disease and cancer.^16^, ^17^, ^18^ Ghosh et al.^19^ reported that TLR4 deficiency exhibited a protective effect in ageing adipose tissue inflammation. Caso et al.^20^ found that TLR4‐deficient mice only presented with a smaller infarction volume and reduced inflammatory response after experimental stroke. Furthermore, the vital role of TLR4‐mediated NLRP3 inflammasome activation and neuroinflammation in CNS diseases is well established. However, few studies have explored the link between TLR4 and NLRP3 inflammasome‐associated microglial pyroptosis after SCI.

Therefore, in the present study, the differentially expressed proteins (DEPs) between TLR4‐knockout (TLR4‐KO) and wild‐type (WT) mice after SCI were identified by using tandem mass tag (TMT)‐based proteomics experiments. Furthermore, we investigated the potential molecular mechanisms of TLR4‐induced pyroptosis after SCI by using a series of in vivo and in vitro molecular biological experiments.

## 2 MATERIALS AND METHODS

### 2.1 Animal

TLR4‐KO and WT C57BL/6 female mice (8–12 weeks old) were obtained from the Laboratory Animal Center of the Chinese Academy of Science (Shanghai, China) and housed individually in sterile experimental cages. All cages were individually ventilated, and the temperature and humidity were controlled and kept at 22 ± 1°C and 45%–55%, respectively. All procedures involving animals were carried out strictly following the guide approved by Fudan University's Animal Care and Use Committee (2019‐JS‐053).

### 2.2 SCI model establishment and drug treatment

The mouse SCI model was established according to a previously reported study.^21^ Briefly, mice were first weighed before anesthesia using 1% pentobarbital sodium (35 mg/kg, intraperitoneally [i.p.] injected). A T8‐9 laminectomy was performed to expose the spinal cord. In the SCI group, the spinal cord was subjected to lateral compression injury for 60 s by Dumont‐type forceps with a spacer of 0.2 mm. On the other hand, only laminectomy was conducted in the Sham group. After the operation, all mice were individually housed and received an intramuscular injection of antibiotics (20 000 units penicillin) for 3 consecutive days. The bladders of mice in the SCI group were massaged manually to help void twice a day until spontaneous voiding resumed.

According to the needs of a particular experiment, the mice received an i.p. injection of SB1518 (30 mg/kg, 100 µl, once a day for 7 consecutive days, or an equivalent amount of Dimethyl sulphoxide (DMSO) for the control; Selleck Chemicals, Houston, TX, USA), fludarabine (40 mg/kg, 100 µl, once a day for 7 consecutive days, or an equivalent amount of DMSO for the control; Selleck Chemicals), or a tail vein injection of pLVX‐dead‐box helicase 3 X‐linked (DDX3X) (5 ×  10^7^ transducing units [TU] in 100 µl of phosphate‐buffered saline [PBS], once every 2 days for 7 days, equivalent amount of pLVX‐NC for the control) after the operation (SCI or Sham surgery).

On day 7 post‐SCI, mice were sacrificed with an overdose of 4% pentobarbital sodium (i.p. injection) and spinal cord tissues (for the SCI and SCI‐TLR4‐KO groups, about 1 cm length of the spinal cord tissue was harvested at the centre of the injury site, and the same length of spinal cord tissue was collected at a similar site for the Sham group) were collected for subsequent experiments, including real‐time quantitative polymerase chain reaction (RT‐qPCR), Western blot (WB), enzyme‐linked immunosorbent assay (ELISA), immunohistochemical (IHC) and immunofluorescence (IF).

### 2.3 Locomotor function recovery assessment

The neurological function of mice in each group was assessed at predetermined time points after the operation.

For the Basso Mouse Scale (BMS) score, all mice were assessed, including hindlimb performance, including coordination, movement of ankle joint, support of weight, plantar stepping and trunk stability, with scores ranging from 0 (complete paralysis) to 9 (normal).

To evaluate the relationship between the mRNA expression of NLRP3 and the BMS score after SCI in mice, we established three new groups of SCI mice (*N* = 6 for each group), which were divided according to different durations of lateral compression of the spinal cord (30, 60 and 90 s, respectively). And then, the Pearson correlation and linear regression analysis was performed 7 days after injury.

For the Von Frey test, mice were placed on a mesh floor and allowed free movement for 5 min prior to the experiment. Then, the plantar skin of the mouse hind paws received repeated stimulation with a calibrated set of Von Frey monofilaments (0.04–60 g). The positive signs included paw lifting or withdrawal, licking or biting immediately in response to stimulus within 5 s. The paw withdrawal threshold (PWT) of each mouse was calculated as described by previous research.^22^

For further assessment of gross motor capability and coordination of mice after SCI, each mouse was evaluated two times on an accelerating rotarod (0–40 rpm) with an interval of 20 min between experiments. The speed and duration were recorded and averaged for each mouse.

### 2.4 Radiographic assessment

Magnetic resonance imaging (MRI) scans of mice were performed 7 days after SCI to assess the integrity of the spinal cord. In brief, mice were anaesthetised as described above and placed in an MR scanner (3.0 T, Trio Tim, Siemens Medical Solutions, Erlangen, Germany) with a whole‐body coil to obtain MR images of the spinal cord.

### 2.5 Mass spectrometry proteomics analysis

The TMT–liquid chromatography (LC)–mass spectrometry (MS)/MS experiment was conducted and analysed by Oebiotech (Shanghai, China). In brief, total protein in spinal cord tissue (three samples each for SCI‐WT and SCI‐TLR4‐KO groups, collected at 7 days post‐SCI) was extracted, followed by digestion and labelling using a Thermo Fisher Scientific TMT labelling kit (Waltham, MA, USA). Then, the above TMT‐labelled sample was fractionated by basic pH reverse‐phase LC using an Agilent 1100 high‐pressure LC system with fraction combing (Santa Clara, CA, USA). The samples were then loaded onto a trap column (350 nl/min) and analytical column (RP‐C18; New Objective, Littleton, MA, USA) before being analysed using a mass spectrometer (Q‐Exactive HF). Finally, the data were analysed with Proteome Discover v2.4 (Thermo Fisher Scientific).

### 2.6 BV2 cell culture and treatments

In this study, the Chinese Academy of Science cell bank (Shanghai, China) provided the mouse microglial cell line, BV2 cells and cultured in high‐glucose Dulbecco's modified eagle medium (DMEM) containing foetal bovine serum (10%, FBS; Gibco, Carlsbad, CA, USA) and penicillin–streptomycin solution (100 U/ml and 100 mg/ml, respectively, Invitrogen, Carlsbad, CA, USA). Cells were maintained with 5% CO_2_ at 37°C. The plasmids pcDNA3.1‐TLR4 and pcDNA3.1‐DDX3X were constructed by Asia‐Vector Biotechnology (Shanghai, China). The siRNA sequences and negative control siRNA/shRNA were provided by Sangon Biotech (Guangzhou, China) (Table S1). The plasmids and siRNAs were transfected into microglial cells using Lipo3000 (Invitrogen) following the manufacturer's instructions. Then, the cells were exposed to lipopolysaccharides (LPS, 1 µg/ml for 8 h) and adenosine triphosphate (ATP, 5 mM for 2 h) obtained from Sigma–Aldrich (St. Louis, MO, USA) to induce pyroptosis as previously described,^23^, ^24^ with or without the treatment of pacritinib (SB1518; Selleck Chemicals; 10 µM for 48 h) 24 h after siRNA or plasmid transfection. After 8 h, the mRNA and protein were harvested for subsequent experiments.

### 2.7 Enzyme‐linked immunosorbent assay

In short, the relative levels of inflammatory cytokines (interleukin‐1β [IL‐1β] and IL‐18) in tissue homogenates of the spinal cord or cell culture supernatants were measured by ELISA using commercial kits (Sigma–Aldrich) following the manufacturer's protocols.

### 2.8 Cytotoxicity assay

To measure cytotoxicity, released lactate dehydrogenase (LDH) activity in tissue homogenate or cell culture supernatants was measured by an LDH cytotoxicity assay kit (Beyotime, Shanghai, China) in accordance with the supplier's instructions.

### 2.9 Real‐time quantitative polymerase chain reaction

The tissue and cell samples were collected after specific treatment. To extract total RNA, TRIzol reagent (Invitrogen) was used, and cDNA was synthesised followed by PCR amplification in accordance with the supplier's protocols with an iCycler iQ Real‐Time PCR Detection System (Bio‐Rad, Hercules, CA, USA). Then, the relative mRNA expression of genes was calculated and analysed by using the 2^−∆∆Ct^ method, and the housekeeping gene glyceraldehyde 3‐phosphate dehydrogenase (GAPDH) was used for normalisation. The primer sequences of the target genes in this study are provided in Table 1.

**TABLE 1:** Sequences of primers used for real‐time quantitative polymerase chain reaction (RT‐qPCR) in this study

Gene	Primer sequence (forward)	Primer sequence (reverse)
GAPDH	AGGTCGGTGTGAACGGATTTG	TGTAGACCATGTAGTTGAGGTCA
TLR4	ATGGCATGGCTTACACCACC	GAGGCCAATTTTGTCTCCACA
NLRP3	ATTACCCGCCCGAGAAAGG	TCGCAGCAAAGATCCACACAG
GSDMD	CCATCGGCCTTTGAGAAAGTG	ACACATGAATAACGGGGTTTCC
ASC	CTTGTCAGGGGATGAACTCAAAA	GCCATACGACTCCAGATAGTAGC
Caspase‐1	TGGAAATGTGCCATCTTCTTT	TCAGCTCCATCAGCTGAAAC
IL‐1β	CGAAGACTACAGTTCTGCCATT	GACGTTTCAGAGGTTCTCAGAG
IL‐18	ACTTCACTGTACAACCGCA	TCAGTCATATCCTCGAACAC
DDX3X	GGATCACGGGGTGATTCAAGAGG	CTATCTCCACGGCCACCAATGC
BGN	GACAACCGTATCCGCAAAGT	CAAAGGCTCCTGGTTCAAAG

Abbreviations: ASC, associated speck‐like protein containing a caspase recruitment domain; BGN, biglycan; DDX3X, dead‐box helicase 3 X‐linked; GAPDH, glyceraldehyde 3‐phosphate dehydrogenase; IL, interleukin; NLRP3, NLR family pyrin domain containing 3; TLR4, Toll‐like receptor 4.

### 2.10 Immunoprecipitation and Western blot analysis

WB was performed routinely as described in a previous study.^25^ In short, protein from spinal cord tissues (collected at 7 days post‐operation in each group) and cultured BV2 cells (in each group with different treatments as needed) was extracted by using appropriate amounts of radioimmunoprecipitation assay (RIPA) lysis buffer (Beyotime) supplemented with 1% phenylmethylsulphonyl fluoride (PMSF; Beyotime) and 1% protease inhibitor cocktail (Beyotime). After that, Sodium dodecyl sulfate‐polyacrylamide gel electrophoresis (SDS–PAGE) was conducted for the separation of samples using equivalent protein samples of each group before transferred to 0.22 µm polyvinylidene fluoride (PVDF) membranes (Millipore, Burlington, MA, USA). Afterwards, PVDF membranes were soaked in 5% bovine serum albumin (BSA), which was diluted with Tris‐buffered saline with 0.1% Tween 20 (TBST), and blocked for 2 h at 25°C. Then, the PVDF membranes were soaked in primary antibodies including TLR4 (1:1000; #14358S; CST, Danvers, MA, USA), NLRP3 (1:1000; ab214185; Abcam, Cambridge, UK), Gasdermin D (GSDMD) (1:1000; ab209845; Abcam), Caspase‐1 (CASP‐1) (1:1000; ab207802; Abcam), IL‐1β (1:1000; A16288; ABclonal, Woburn, MA, USA), DDX3X (1:1000; ab235940; Abcam), Janus Kinase 2 (JAK2) (1:1000; #3230S; CST), phospho‐(p)‐JAK2 (1:1000; #3771S; CST), Signal Transducer and Activator of Transcription 1 (STAT1) (1:1000; ab239360; Abcam), p‐STAT1 (1:1000; ab109457; Abcam), biglycan (BGN; 1:1000; bs‐7552R; Bioss, Boston, MA, USA), GAPDH (1:5000; ab8245; Abcam) and β‐actin (1:5000; ab8227; Abcam) at 4°C for overnight. On the next day, PVDF membranes were washed using TBST three times (10 min each time), and then horseradish peroxidase (HRP)‐conjugated secondary antibodies were added for incubation at 25°C for 1 h. After being washed with TBST three times (10 minutes each time), PVDF membranes were incubated with the high‐sensitivity enhanced chemiluminescence (ECL) reagents (Thermo Fisher Scientific) to visualise protein bands by Tanon‐5500 (Tanon Science & Technology, Shanghai, China), which were then quantified using densitometric analysis with ImageJ v1.57.

For immunoprecipitation (IP), 1 mg total protein and 5 µg antibodies were mixed for incubation at 4°C for overnight and then incubated with protein A/G PLUS‐Agarose beads (sc‐2003; Santa Cruz Biotechnology, Santa Cruz, CA, USA) at 4°C for an additional 4 h. After being washed with RIPA and PBS, the precipitates were resuspended in SDS loading buffer, and WB analysis was performed as mentioned.

### 2.11 PI/Hoechst 33342 staining

BV2 cell death was evaluated by Hoechst 33342 and propidium iodide (PI) staining in different groups. In brief, after treatments as needed, the cells were washed three times using cold PBS. Then, Hoechst 33342 (10 µg/ml; Beyotime) and PI (10 µg/ml; Beyotime) were used for incubation at 37°C. After 15 min, BV2 cells were observed by a fluorescence microscope (Olympus, Tokyo, Japan), and the proportion of apoptotic cells (PI‐positive) was calculated using ImageJ v1.57 in five random samples of each group.

### 2.12 Reactive oxygen species (ROS) production

Intracellular ROS were measured using 2′,7′‐dichlorofluorescein‐diacetate (DCFH‐DA, Sigma–Aldrich) as described.^26^ BV2 cells were treated with or without BGN‐shRNA (sh‐NC for control) prior to LPS (1 µg/ml, 8 h) and ATP (5 mM, 2 h) exposure. Then, DCFH‐DA (10 µM) was added and incubated for 0.5 h at 37°C prior to washing three times using serum‐free medium. An Olympus fluorescence microscope (Tokyo, Japan) was used to observe the staining of DCFH‐DA (485 nm excitation, 535 nm emission), and flow cytometry was applied to analyse the ROS production in three random samples of each group.

### 2.13 Histological analysis

On day 7 after surgery, mice (*N* = 5 each group) were sacrificed and perfused with 4% paraformaldehyde (PFA) in 0.01 M PBS. Then, the spinal cord tissues (0.5 cm above and below the injury site) were harvested for post‐fixation in 4% PFA overnight and embedded in paraffin. For IHC staining was performed as described previously. In brief, to block endogenous peroxidase, deparaffinised transverse sections (25 µm) were incubated with 3% H_2_O_2_ in methanol. After 10 min, serum‐blocking solution was added to block for 30 min. After that, sections were exposed to primary antibodies of NLRP3 (1:100), CASP‐1 (1:100, ab1872; Abcam) or DDX3X (1:100) for 2 h, and HRP‐conjugated secondary antibodies were added to incubate for 30 min. Then, slides were incubated with 3,3′‐diaminobenzidine tetrahydrochloride (DAB) for 10 min to visualise the bound antibodies. All images were acquired by a Leica DMI6000B microscope (Wetzlar, Germany). Semiquantitative analysis of staining was carried out by Image‐Pro Plus software (v6.0) in five random samples of each group and presented as integrated optical density (IOD)/area.

Haematoxylin and eosin (H&E) and Nissl staining were performed to assess histopathological changes and neuronal survival in spinal cord tissue according to the manufacturer's protocols (Beyotime).

### 2.14 Immunofluorescence staining

IF staining was conducted according to a previous study.^21^ Briefly, spinal cord tissue samples in different groups were collected at 7 days post‐SCI, and 12‐µm‐thick frozen sections were prepared. BV2 cells were soaked in 4% PFA to fix for approximately 10 min at 25°C. After washing three times (10 min each time) with PBS, all samples were permeabilised using Triton X‐100 (0.3%, 15 min) before being blocked in 5% BSA for half an hour. Then, the samples were incubated against the primary antibodies, including NLRP3 (1:100, a12694;ABclonal), DDX3X (1:100, ab196032; Abcam) or Iba‐1 (1:100, ab178846; Abcam), at 4°C for overnight. This was followed by Dylight488‐ and Dylight594‐conjugated secondary antibodies (1:1000; Jackson ImmunoResearch, West Grove, PA, USA) for 2 h at 25°C. Double IF staining of TUNEL and NeuN (1:100, ab177487; Abcam) or Glial ibrillary acidic protein (GFAP) (1:100, ab7260; Abcam) in spinal cord tissue sections was conducted according to the manufacturer's protocol (In Situ Cell Death Detection kit, Roche, Germany). After being incubated at 4°C for overnight, sections were exposed to secondary antibodies (rhodamine‐conjugated, 1:100, Jackson ImmunoResearch). Finally, the nuclei in the samples were captured by counterstaining with 4′,6‐diamidino‐2‐phenylindole (DAPI), and the images were acquired with a Leica DMI6000B microscope. The fluorescence intensity and the proportion of TUNEL‐positive neuron cells or astrocytes were analysed using ImageJ v1.57 in five random samples from each group.

### 2.15 Luciferase assay

A DDX3X promoter (–2000/+200 bp) of C57BL/6J mouse genomic DNA was cloned and inserted into the pGL3 basic vector (Promega, Madison, WI, USA). Subsequent deletion generated −1200/+200 bp and −600/+200 bp reporter plasmids. The TLR4 plasmid (pc‐TLR4), luciferase reporter plasmids and pRL‐TK reporter plasmid (control reporter) were transfected into BV2 cells using Lipofectamine 2000 (Invitrogen) before treatment with the JAK2 inhibitor SB1518. After transfection for 72 h, the cells were harvested, and the Dual‐Luciferase Reporter Assay (Promega) was used to detect the luciferase activity of each group following the manufacturer's protocols. Renilla luciferase activity was referred as an internal control to calculate the relative luciferase activity.

### 2.16 Chromatin immunoprecipitation assay

Chromatin immunoprecipitation (ChIP) experiment was conducted in accordance with our previous study.^21^ A total of 10^7^ BV2 cells (cultured in 10 cm cell culture dishes with or without TLR4 overexpression) were treated with 1% formaldehyde at 25°C for 10 min to crosslink DNA and then incubated with 125 mM glycine for 2 min to stop the reaction. Then, after being washed three times with ice‐cold PBS, the cells were harvested for resuspension in 1% SDS lysis buffer on ice for 10 min. Thereafter, the genomic DNA was fragmented into 300–700 bp lengths for 30 min by sonication (5 × 10 s intervals). The ninefold ChIP dilution buffer was added to dilute the prepared chromatin and precleared with Protein G agarose for 2 h at 4°C. Then, lysates were incubated with anti‐STAT1 antibody (normal IgG was used as a control) at 4°C for overnight. The subsequent day, the prepared Protein G agarose was added, and the immunocomplexes were incubated at 4°C for 1 h before washing one time in low salt, high salt, LiCl and twice in Tris‐EDTA (TE) buffer. After elution and reverse crosslinking, a PCR purification kit (Qiagen, Hilden, Germany) was adopted to purify DNA, which was analysed using qPCR and agarose gel electrophoresis. Primers for ChIP‐qPCR are listed in Table S2.

### 2.17 Statistical analysis

All data are presented as the mean ± standard deviation (SD). To compare differences among groups, Student's unpaired *t*‐test and one‐way analysis of variance (ANOVA) followed by Dunnett's test were adopted. Pearson's correlation analysis and linear regression were performed to measure the correlation between the mRNA level of NLRP3 and the BMS score. All analyses were performed using SPSS v18.0 software (Chicago, IL, USA). *p* < .05 was considered to be statistically significant.

## 3 RESULTS

### 3.1 TLR4 and NLRP3‐mediated neuroinflammation are upregulated in SCI mice

**FIGURE 1:**
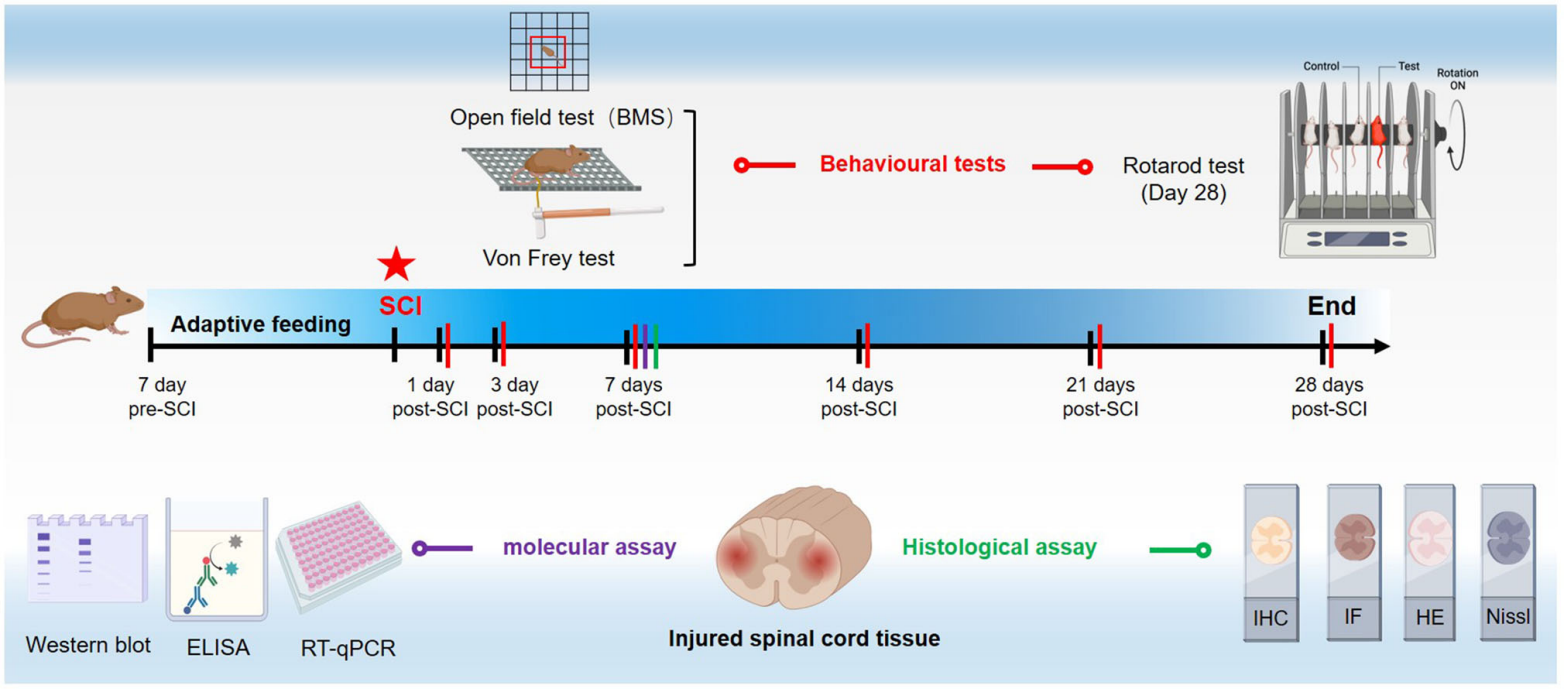
Schematic diagram of the experimental design and main molecular biological assays at predetermined time points of this study

As indicated in Figure 1, mice were fed adaptively for 1 week before the formal experiment. All mice received different kinds of locomotor function assessments at 1, 3, 7, 14, 21 and 28 days after operation, and the spinal cord tissues in each group were collected 7 days post‐SCI for subsequent biomolecular experiments (including WB, ELISA, RT‐qPCR and histological assays).

Figure S1A,B shows representative intraoperative photographs of spinal cords and the appearance of the hind limbs (photographed at 7 days post‐injury) in the SCI and control groups. Additionally, the lower BMS score and PWT (Figure S1C) in SCI mice indicated the successful establishment of the SCI mouse model in our study. In addition, a total of 38 mouse spinal cord tissue samples (19 SCI and 19 control) were harvested to detect the relative mRNA levels of *TLR4, NLRP3* and inflammatory‐related cytokines (*IL‐18* and *IL‐1β*) 7 days after surgery. The results of ELISA (Figure S1D) and RT‐qPCR (Figure S1E) showed a significant increase in IL‐18, IL‐1β and mRNA levels of *TLR4* and *NLRP3* in the SCI group. Importantly, the mRNA level of *NLRP3* was negatively correlated with the BMS score (partially reflecting the severity of tissue injury) 7 days post‐SCI by linear correlation analysis (*r* = ‐0.6226, *p* < .01) (Figure S1F). These preliminary results suggested that the mRNA level of *NLRP3* was positively correlated with the severity of SCI, whereas TLR4 may play a crucial role in SCI.

### 3.2 TLR4 deficiency alleviated SCI by inhibiting NLRP3 inflammasome‐mediated pyroptosis in vivo

**FIGURE 2:**
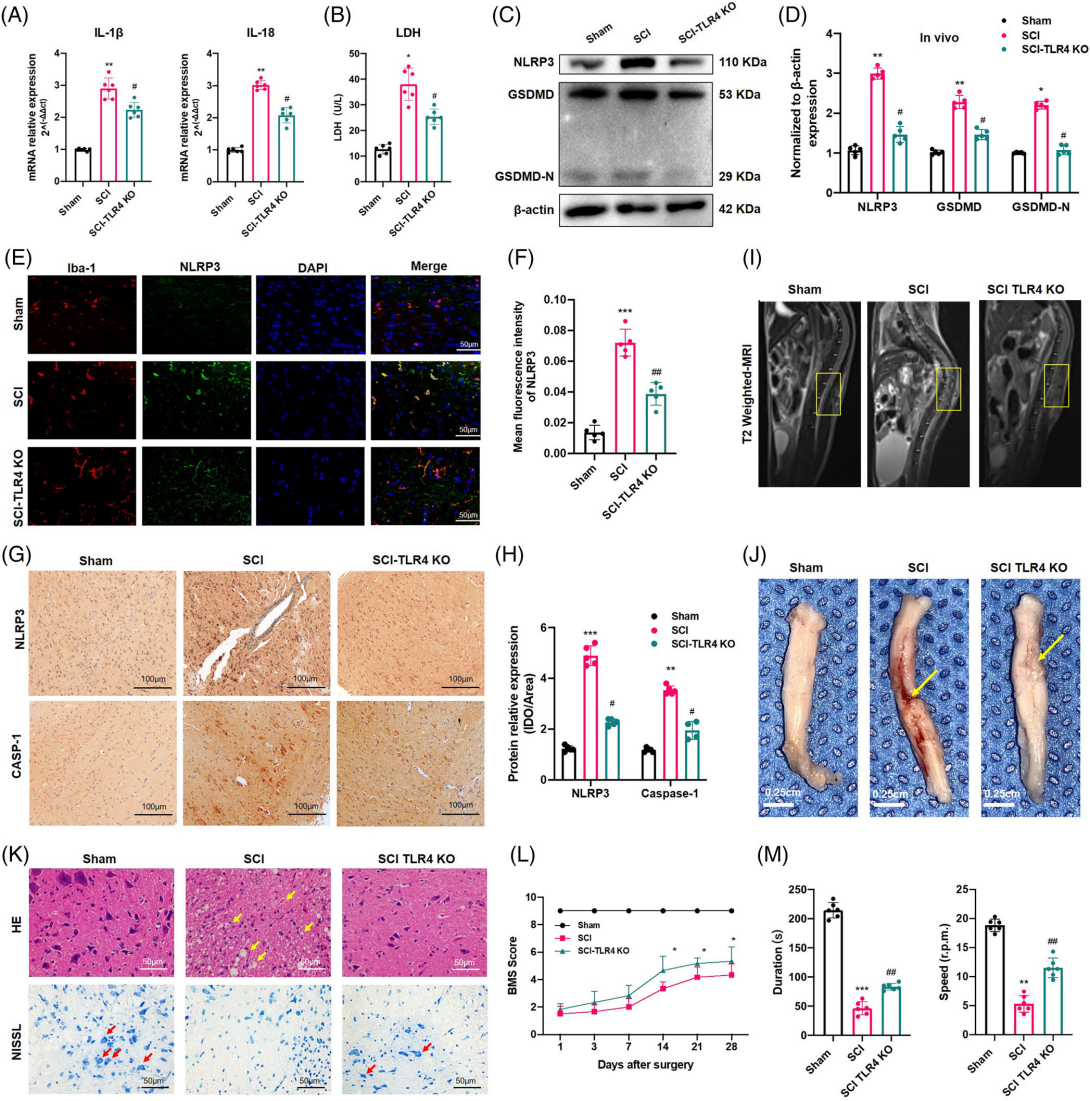
Toll‐like receptor 4 (TLR4) deficiency alleviates spinal cord injury (SCI) via suppressing NLR family pyrin domain containing 3 (NLRP3) inflammasome activation and pyroptosis. (A and B) Interleukin‐1β (IL‐1β), IL‐18 and lactate dehydrogenase (LDH) levels in the spinal cord homogenates of the Sham, SCI and SCI‐TLR4‐knockout (KO) mice (*N* = 6). *p < .05, **p < .01 versus Sham, ^#^ *p* < .05 versus SCI. (C and D) Representative immunoblot and quantification of NLRP3, GSDMD, GSDMD‐N proteins in each group (*N* = 5). *p < .05, **p < .01 versus Sham, ^#^ *p* < .05 versus SCI. (E and F) Representative two‐photon excitation images of immunofluorescence of Iba‐1 and NLRP3 as well as semiquantitative analysis acquired from Sham, SCI and SCI‐TLR4‐KO mice 7 days post‐injury or Sham surgery. Nuclei are stained with 4′,6‐diamidino‐2‐phenylindole (DAPI) (blue). Scale bar = 50 µm. (G and H) Immunopositive particles for NLRP3 and CASP‐1 and quantification 7 days post‐injury or Sham surgery in wild‐type (WT) or TLR4‐KO mice (*N* = 5). Scale bar = 100 µm. **p < .01, ***p < .001 versus Sham, ^#^ *p* < .05 versus SCI. (I) Representative T2‐weighted magnetic resonance (MR) images of spines 7 days after SCI or Sham surgery. (J) Gross appearance of spinal cord tissue from the three groups. The yellow arrow indicates the injury site. Scale bar = 0.25 cm. (K) Representative haematoxylin and eosin (H&E) staining and Nissl staining of the spinal cord tissue in the three groups. Scale bar = 50 µm. (L) Basso Mouse Scale (BMS) scores of the different groups on days 1, 3, 7, 14, 21 and 28 after SCI or Sham surgery (*N* = 6). *p < .05 SCI‐TLR4‐KO versus SCI. (M) Rotarod test at day 28 post‐surgery in different groups (*N* = 6). **p < .01, ***p < .001 versus Sham, ^##^ *p* < .01 versus SCI

To identify the regulatory role of TLR4 in NLRP3 inflammasome‐mediated pyroptosis after SCI, spinal cord segments from Sham, SCI and SCI‐TLR4‐KO mice were collected 7 days post‐operation. Firstly, WB results confirmed the deficiency of TLR4 in the TLR4‐KO group (Figure S2). As shown in Figure 2A, the elevated levels of IL‐1β and IL‐18 post‐SCI decreased markedly in the SCI‐TLR4‐KO group. In addition, a similar expression pattern of LDH was detected by ELISAs (Figure 2B). Additionally, the increased mRNA levels of NLRP3 inflammasome‐related genes (*NLRP3*, *GSDMD*, *ASC*, *Caspase‐1*) in the SCI group all showed an obvious decline in the SCI‐TLR4‐KO group (Figure S2). Consistently, similar expression trends of the protein levels of NLRP3 and GSDMD in the three groups were revealed by WB (Figure 2C,D). Comparing with the Sham group, IF results also showed a greater level of NLRP3 (green fluorescence), which was colocalised with Iba‐1 (a marker of microglia, red fluorescence), in the SCI group, which was again reduced in the SCI‐TLR4‐KO group (Figure 2E,F). Similar results were also found by IHC for NLRP3 and CASP‐1 (Figure 2G,H). The representative T2‐weighted MRI images of spinal cords showed that there was a more severe tissue injury (more obvious heterogeneous signal intensity with an ill‐defined margin) in the SCI group than in the SCI‐TLR4‐KO group (Figure 2I), which was further confirmed by the gross appearance of the harvested specimens (Figure 2J). H&E and Nissl staining also revealed alleviated tissue injury and less neuronal loss (characterised by damaged nuclei and shrunken cytoplasm, as indicated by arrow) in the SCI‐TLR4‐KO group than in the SCI group (Figure 2K). In addition, to better evaluate neuronal survival, we performed a TUNEL assay with NeuN co‐stain and the results also indicated that TLR4 deficiency alleviates neuronal apoptosis after SCI (Figure S2). Additionally, IF staining of GFAP suggested the suppressed activation of astrocytes in the SCI‐TLR4‐KO group compared with the SCI group (Figure S2). Furthermore, a higher BMS score and PWT were recorded in the SCI‐TLR4‐KO group than in the SCI group at all the indicated time points (Figures 2L and S2). The results of the rotarod test (28 days post‐SCI) also implied better recovery of motor function in the SCI‐TLR4‐KO group than in the SCI group (Figure 2M). In summary, these results demonstrated that TLR4 deficiency mitigated pyroptosis and promoted functional recovery after SCI.

### 3.3 TLR4‐knockdown attenuated NLRP3 inflammasome activation and pyroptosis in vitro

**FIGURE 3:**
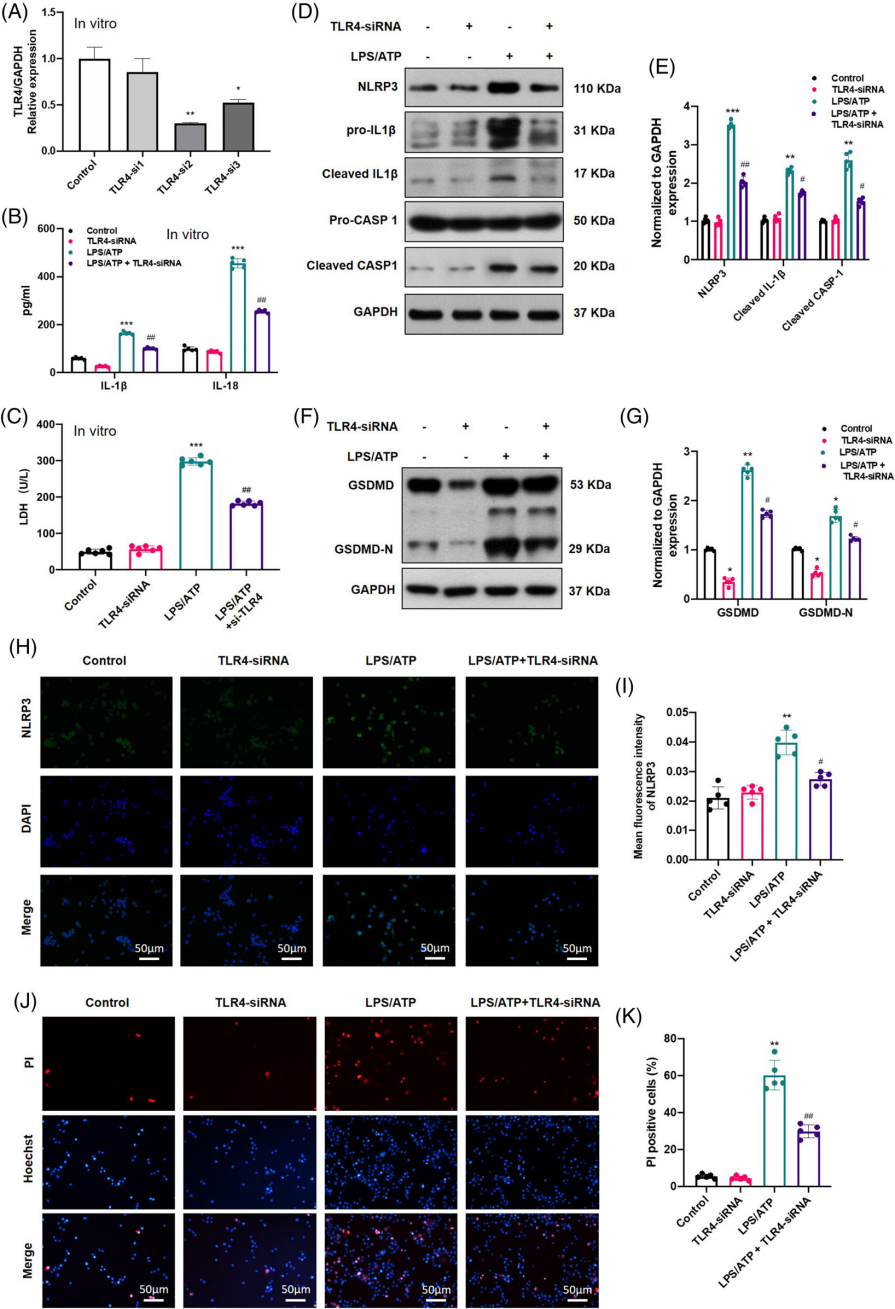
Toll‐like receptor 4‐knockdown (TLR4‐KO) inhibited NLR family pyrin domain containing 3 (NLRP3) inflammasome activation and pyroptosis in lipopolysaccharides (LPS)/adenosine triphosphate (ATP)‐induced microglia. (A) The efficiency of TLR4 siRNAs detected by real‐time quantitative polymerase chain reaction (RT‐qPCR) (*N* = 3). (B and C) Levels of inflammatory cytokines and lactate dehydrogenase (LDH) in the cell culture supernatant of different treated groups (*N* = 6). (D–G) Representative immunoblot of pyroptosis‐related protein expression and statistical comparison in microglial cells in different treatment groups (*N* = 5). (H and I) Representative immunofluorescence images of NLRP3 (green) and quantification in each group (*N* = 5). Nuclei are stained with 4′,6‐diamidino‐2‐phenylindole (DAPI) (blue). Scale bar = 50 µm. (J and K) propidium iodide (PI)/Hoechst staining of microglia and quantification in different treatment groups (*N* = 5). Scale bar = 50 µm. *p < .05, **p < .01, ***p < .001 versus control, ^#^ *p* < .05, ^##^ *p* < .01 versus LPS/ATP

To further confirm the inhibitory effects of TLR4 on pyroptosis in vitro, BV2 cells were treated with LPS (1 µg/ml, 8 h, as a priming signal) and ATP (5 mM, 2 h, as a secondary signal) to activate pyroptosis with or without *TLR4* siRNA treatment. We observed that *TLR4* mRNA expression was efficiently inhibited by *TLR4* siRNA2 (TLR4_si2), which is shown in Figure 3A. As expected, *TLR4* siRNA significantly decreased the levels of inflammatory cytokines (IL‐1β and IL‐18) and LDH in the cell culture supernatant compared to the LPS/ATP group (Figure 3B,C). Moreover, the protein levels of pyroptosis‐associated molecules (*NLRP3*, *GSDMD*, *cleaved IL‐1β* and *cleaved CASP‐1*) all decreased in the presence of *TLR4* siRNA (Figure 3D–G). Correspondingly, the lower IF intensity of NLRP3 and lower PI‐positive staining in the LPS/ATP + TLR4‐siRNA group also confirmed that the *TLR4* knockdown inhibited NLRP3 inflammasome activation and pyroptosis. Taken together, the above results further demonstrated the key role of TLR4 in microglial pyroptosis: TLR4 downregulation could alleviate BV2 microglial pyroptosis by reducing NLRP3 inflammasome activation in vitro.

### 3.4 TLR4 upregulated DDX3X and activated JAK2/STAT1 signalling after SCI

**FIGURE 4:**
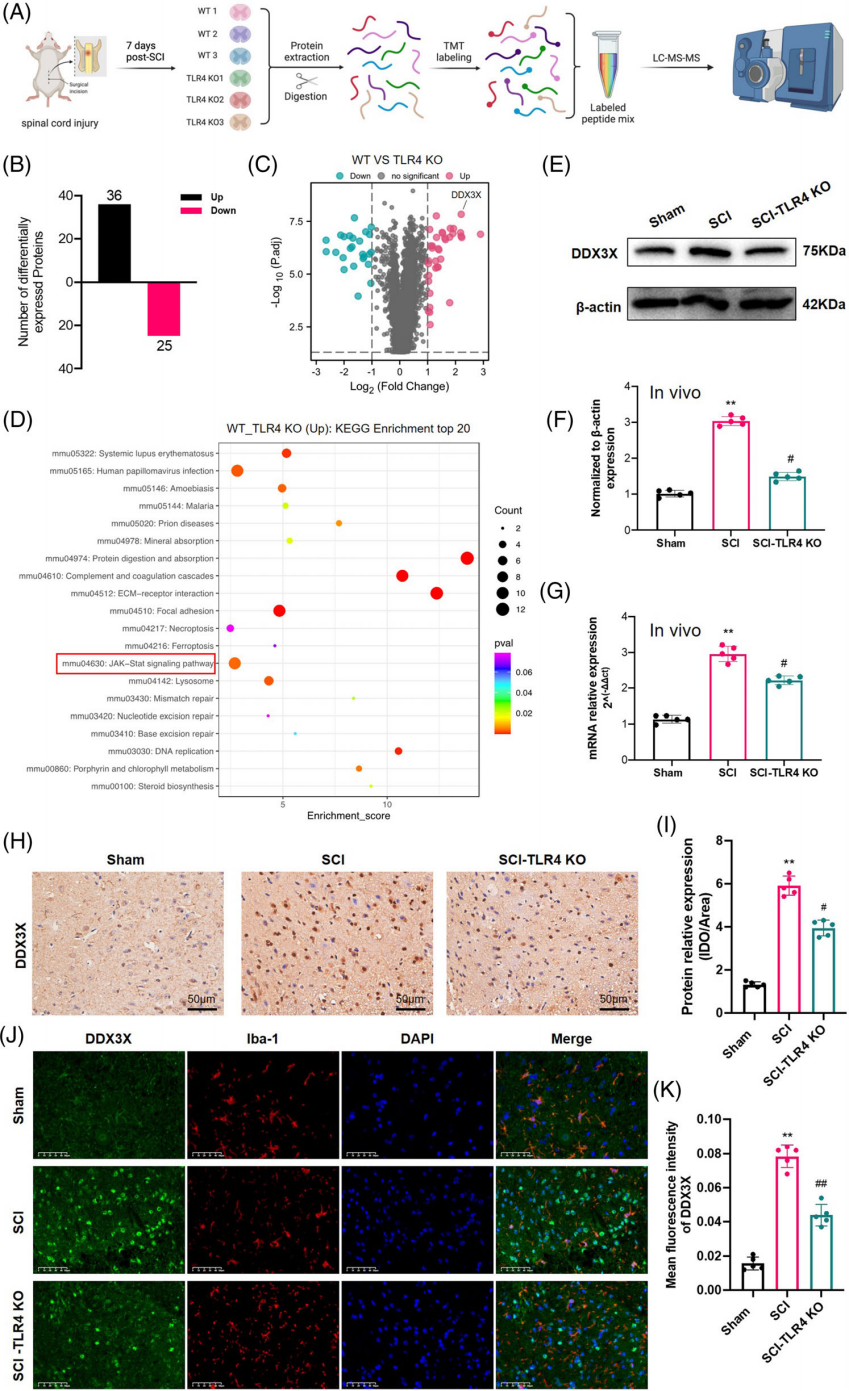
The upregulation of dead‐box helicase 3 X‐linked (DDX3X) after spinal cord injury (SCI) was dependent on Toll‐like receptor 4 (TLR4). (A) The workflow of tandem mass tag (TMT)‐based proteomics analysis. (B and C) The number and volcano map of differentially expressed proteins (DEPs) between wild‐type (WT) and TLR4‐knockout (KO) mice after SCI. (D) KEGG pathway analysis of upregulated DEPs in the SCI group compared with the SCI‐TLR4‐KO group. (E–G) Representative immunoblot and mRNA levels of DDX3X and semiquantitative analysis in WT and TLR4‐KO mice after SCI (*N* = 5). (H and I) Representative photomicrographs and semiquantitative analysis of immunohistochemistry staining for DDX3X in each group (*N* = 5). Scale bar = 50 µm. (J and K) Representative immunofluorescence images showing DDX3X (green) and Iba‐1 (red) expression and quantification analysis at day 7 post‐injury (*N* = 5). Nuclei are stained with 4′,6‐diamidino‐2‐phenylindole (DAPI) (blue). Scale bar = 50 µm. **p < .01 versus Sham, ^#^ *p* < .05, ^##^ *p* < .01 versus SCI

To explore in detail the underlying mechanism by which TLR4 promotes pyroptosis after SCI, TMT‐MS‐based proteomics analysis of spinal cord tissues from the SCI and SCI‐TLR4‐KO groups 7 days post‐operation was performed. The screening criteria for DEPs were set with *p* < .05 and | log_2_(fold‐change)| > 1. A total of 61 DEPs (36 upregulated and 25 downregulated in the SCI group) were identified (Figure 4A–C). KEGG pathway enrichment analysis indicated that the JAK‐STAT pathway was one of the enriched pathways of the DEPs (Figure 4D, red box highlighted). Among the DEPs, DDX3X was one of the most differentially upregulated proteins when comparing the SCI group with the SCI‐TLR4‐KO group, while both WB and qPCR confirmed that its increased expression level after SCI was regulated by TLR4 (Figure 4E–G). Furthermore, the results of IHC and IF staining also displayed a reduced expression level of DDX3X in the SCI‐TLR4‐KO group compared with the SCI group. For in vitro analysis, increased mRNA and protein levels of DDX3X were also detected in LPS/ATP‐induced BV2 microglial cells (Figure S3), which significantly declined in the presence of *TLR4* siRNA. We also found that the protein levels of p‐JAK2/JAK2 and p‐STAT1/STAT1 decreased significantly both in the SCI‐TLR4‐KO group and in the LPS/ATP + TLR4‐siRNA group (Figure S3). Collectively, *DDX3X* was highly expressed, and JAK2/STAT1 signalling was activated after SCI, which were both dependent on TLR4.

### 3.5 DDX3X abolished the anti‐pyroptotic effect of TLR4 deficiency after SCI

**FIGURE 5:**
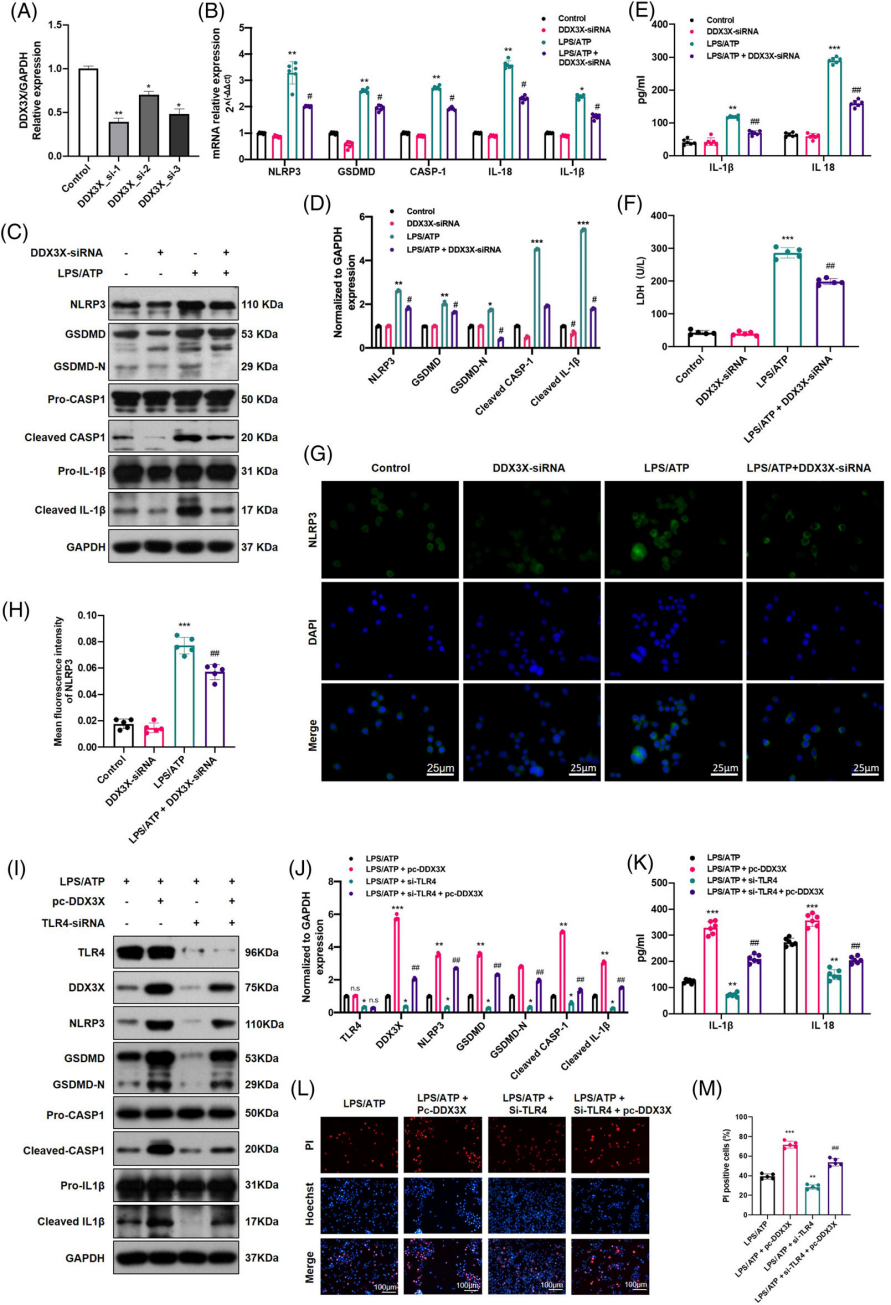
Dead‐box helicase 3 X‐linked (DDX3X) reversed the inhibition of NLR family pyrin domain containing 3 (NLRP3) inflammasome activation and pyroptosis in Toll‐like receptor 4 (TLR4)‐deficient microglia. (A) The downregulation efficiency of siRNAs was measured by real‐time quantitative polymerase chain reaction (RT‐qPCR) (*N* = 3). (B–D) mRNA and protein levels of pyroptosis‐related genes (NLRP3, GSDMD, CASP‐1, interleukin [IL]‐1β and IL‐18) in different treated microglia (*N* = 5). (E and F) The levels of inflammatory factors (IL‐1β and IL‐18)（*N* = 6） and lactate dehydrogenase (LDH) in each group (*N* = 5). (G and H) Representative immunofluorescence images of NLRP3 and semiquantitative analysis in each group (*N* = 5). Scale bar = 100 µm. (I and J) Representative immunoblot and statistical comparison of TLR4, DDX3X and pyroptosis‐associated proteins in lipopolysaccharides (LPS)/adenosine triphosphate (ATP)‐induced microglia with or without the treatment of pc‐DDX3X and TLR4 siRNA (*N* = 3). (K) IL‐1β and IL‐18 levels in the above groups of microglia (*N* = 6). (L and M) Representative images of propidium iodide (PI)/Hoechst staining and the relative ratio of PI‐positive cells in different treated microglia (*N* = 5). Scale bar = 100 µm. *p < .05, **p < .01, ***p < .001 versus control, ^#^ *p* < .05, ^##^ *p* < .01 versus LPS/ATP

**FIGURE 6:**
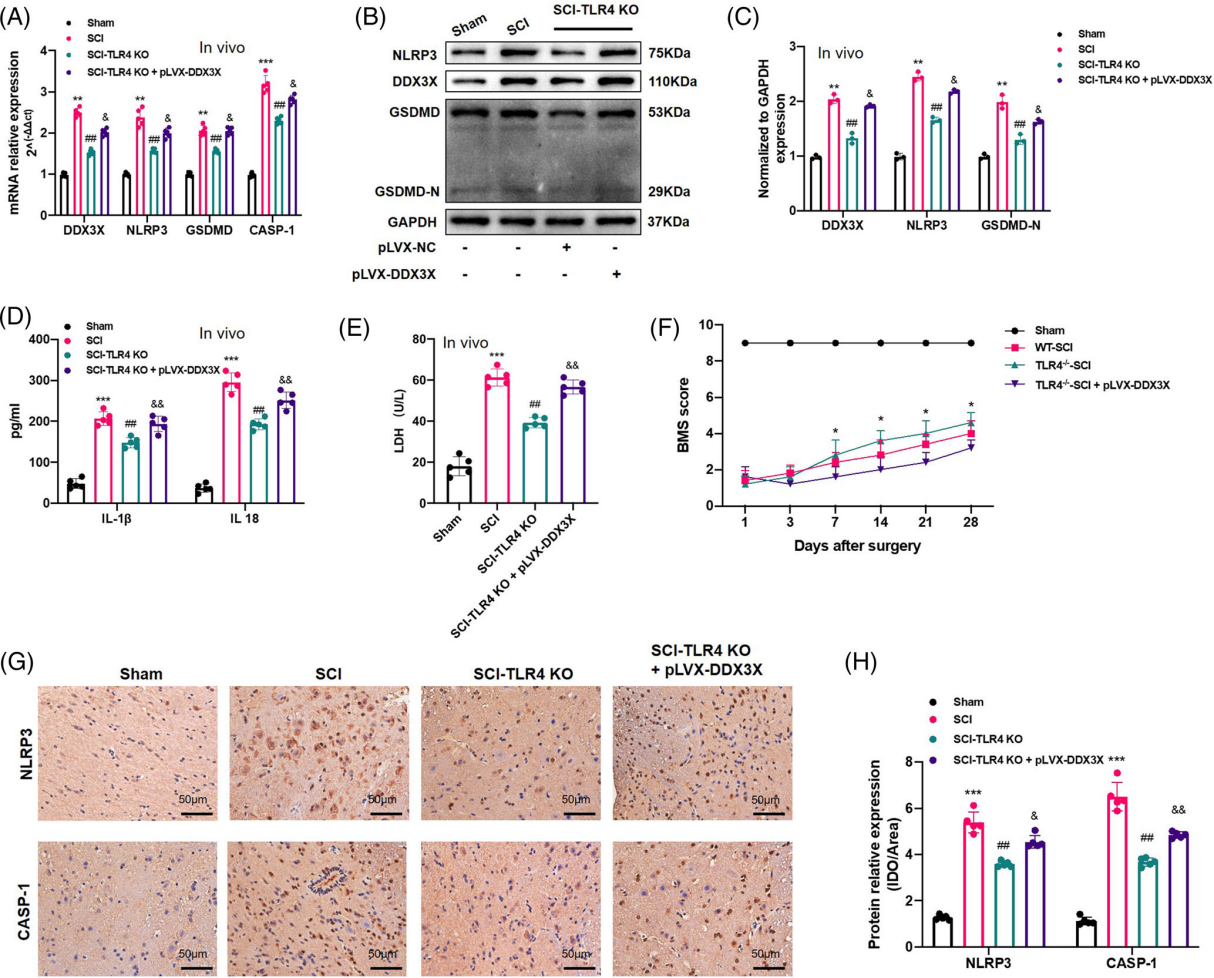
Dead‐box helicase 3 X‐linked (DDX3X) abrogated anti‐pyroptotic role of Toll‐like receptor 4 (TLR4) deficiency after spinal cord injury (SCI) in mice. (A–C) Relative mRNA level (A, *N* = 5) and representative immunoblot of DDX3X and pyroptosis‐associated genes (NLR family pyrin domain containing 3 [NLRP3], GSDMD, GSDMD‐N) and statistical comparison in each group of mice at 7 days post‐operation (B and C, *N* = 3). (D and E) The release of interleukin (IL)‐1β, IL‐18 and lactate dehydrogenase (LDH) in spinal cord homogenate measured by enzyme‐linked immunosorbent assay (ELISA) kits, respectively, at 7 days after SCI or Sham surgery in each group (*N* = 5). (F) Basso Mouse Scale (BMS) score at 1, 3, 7, 14, 21 and 28 days in each group (*N* = 5). (G and H) Representative immunohistochemical (IHC) staining and analysis of NLRP3 and CASP‐1 in each group (*N* = 5). Scale bar = 50 µm. **p < .01, ***p < .001 versus Sham, ^##^ *p* < .01 versus SCI, ^&^ *p* < .05, ^&&^ *p* < .01 versus SCI‐TLR4‐knockout (KO) group

To further elucidate the key role of DDX3X in microglial pyroptosis, we up‐ and downregulated *DDX3X* expression in BV2 cells using specific *DDX3X* siRNAs and overexpression plasmids, respectively. As indicated in Figure 5A–D, the qPCR and WB results indicated that *DDX3X* siRNA effectively alleviated NLRP3 inflammasome activation and pyroptosis in LPS/ATP‐treated BV2 microglia. Likewise, the increased levels of IL‐1β, IL‐18 and LDH in the LPS/ATP group were all reversed by *DDX3X* siRNA (Figure 5E,F). IF staining of NLRP3 also displayed a similar trend to the WB and qPCR results. On the other hand, the transfection of the *DDX3X* overexpression plasmid effectively upregulated the protein levels of DDX3X and obviously reversed the anti‐pyroptotic effect of *TLR4* siRNA, as shown by the WB results in Figure 5I,J. Also, there were higher levels of inflammatory cytokines (IL‐1β and IL‐18) and a higher ratio of PI‐positive cells after *DDX3X* overexpression in *TLR4* siRNA‐treated BV2 microglial cells (Figure 5K–M). For in vivo analysis, stable *DDX3X* overexpression in TLR4‐KO mice further exacerbated pyroptosis (all the parameters were detected in spinal cord tissue collected at 7 days post‐operation) and hindered the recovery of motor function (lower BMS score) after SCI (Figure 6A–H). Taken together, these results identified DDX3X as an essential factor in mediating TLR4‐induced NLRP3 inflammasome activation and microglial pyroptosis after SCI in mice.

### 3.6 TLR4 activated the DDX3X/NLRP3 signalling axis via the JAK2/STAT1 pathway

**FIGURE 7:**
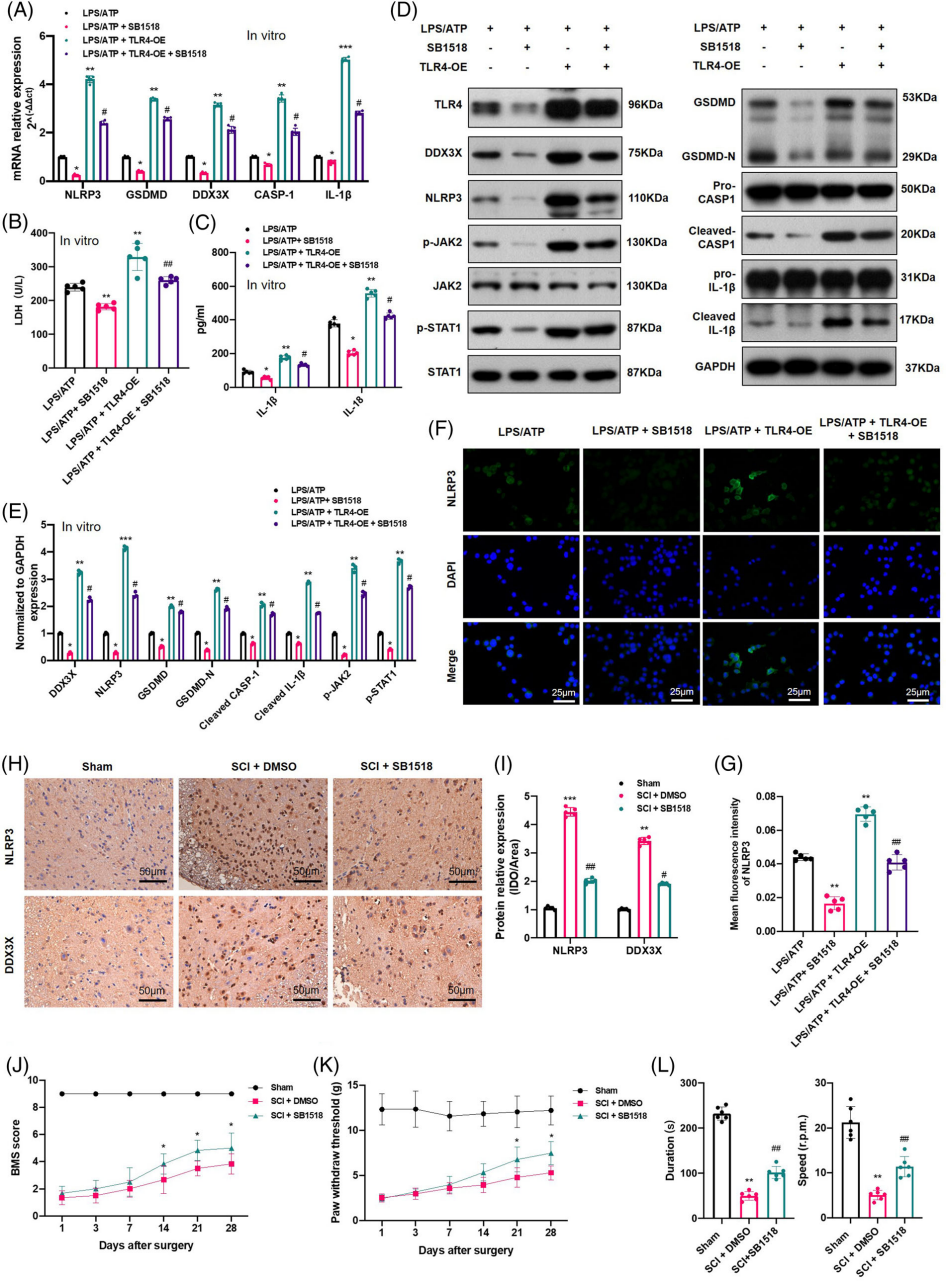
Toll‐like receptor 4 (TLR4) promoted dead‐box helicase 3 X‐linked (DDX3X)/NLR family pyrin domain containing 3 (NLRP3) axis‐mediated pyroptosis via the JAK2/STAT1 pathway. (A) Relative mRNA levels of DDX3X and pyroptosis‐related genes (NLRP3, GSDMD, CASP‐1, interleukin [IL]‐1β) in lipopolysaccharides (LPS)/adenosine triphosphate (ATP)‐stimulated microglial cells with or without TLR4 overexpression and SB1518 treatment (10 µM, 48 h) (*N* = 5). (B and C) Released levels of lactate dehydrogenase (LDH) and inflammatory cytokines (IL‐1β and IL‐18) in the culture supernatants of each group (*N* = 5). (D and E) Representative immunoblot and statistical comparison of DDX3X and pyroptosis‐related proteins in each group (*N* = 3). (F and G) Representative immunofluorescence images of NLRP3 and quantification in different treated microglia (*N* = 5). (H and I) Representative immunohistochemical staining for NLRP3 and DDX3X and semiquantitative analysis at day 7 after spinal cord injury (SCI) or Sham surgery (*N* = 5). (J and K) Basso Mouse Scale (BMS) score and paw withdrawal threshold of the different groups on days 1, 3, 7, 14, 21 and 28 after SCI or Sham surgery with/without SB1518 treatment (*N* = 6). (L) Rotarod test at day 28 post‐surgery in different groups (*N* = 6). *p < .05, **p < .01, ***p < .001 versus LPS/ATP or Sham, ^#^ *p* < .05, ^##^ *p* < .01 versus LPS/ATP + TLR4‐OE or SCI + DMSO

As mentioned in the TMT‐based proteomics analysis, the JAK‐STAT pathway was enriched after SCI; therefore, to identify whether the JAK2/STAT1 axis was involved in TLR4‐induced DDX3X/NLRP3 signalling axis activation, the mRNA and protein levels of JAK2, STAT1, and their phosphorylated forms (p‐JAK2 and p‐STAT1) were examined in vivo and in vitro. WB results showed that *TLR4* deficiency reduced the levels of p‐JAK2/JAK2 and p‐STAT1/STAT1 (as described in Section 3.4). To clarify whether TLR4 promoted pyroptosis by activating the JAK2/STAT1 pathway, SB1518 (a JAK2 inhibitor, 10 µM, 48 h) was used to treat BV2 cells with or without transfection of the *TLR4* overexpression plasmid following LPS/ATP treatment. As shown in Figure 7A,D,E, *TLR4* overexpression further promoted the expression of *DDX3X*, pyroptosis‐associated genes (*NLRP3*, *GSDMD*, *GSDMD‐N*, *Pro‐IL1β*, *Pro‐CASP1*), and the protein levels of p‐JAK2 and p‐STAT1. The ELISA results of IL‐1β, IL‐18 and LDH (Figure 7B,C), and IF staining of NLRP3 (Figure 6F–G) were all consistent with the WB and qPCR results. However, SB1518 partially abolished the pro‐pyroptotic function of *TLR4* overexpression in BV2 cells, as mentioned above (Figure 7A–G). Importantly, because SB1518 also targets FLT3, we explored the role of FLT3 in BV2 pyroptosis using FLT3‐siRNA. The results (as shown in Figure S4) indicated that there was no significant decrease in pyroptosis‐associated proteins (*NLRP3, GSDMD, GSDMD‐N, cleaved CASP‐1*) and LDH in the LPS/ATP + si‐FLT3 group when compared with the LPS/ATP group. These results implied that the anti‐pyroptotic role of SB1518 is mediated by inhibiting the JAK2/STAT1 signalling axis. Furthermore, for in vivo analysis, SB1518 was injected i.p. (30 mg/kg, once a day for 7 days) after SCI, and the protein levels of JAK2, p‐JAK2, STAT1 and p‐STAT1 in spinal cord tissue were all inhibited compared with those in the SCI group (Figure S4). As expected, SB1518 markedly suppressed the levels of *NLRP3* and *DDX3X* (indicated by IHC in Figure 7H,I). Also, SB1518 treatment facilitated locomotion recovery after SCI, as demonstrated by the higher BMS scores, higher PWT and better gross motor capability when compared with SCI + DMSO group (Figure 7J–L). In short, activation of the JAK2/STAT1 signalling pathway was involved in TLR4‐mediated *DDX3X* upregulation, which further led to NLRP3 inflammasome activation and pyroptosis in microglia.

### 3.7 DDX3X is a direct transcriptional target of STAT1

**FIGURE 8:**
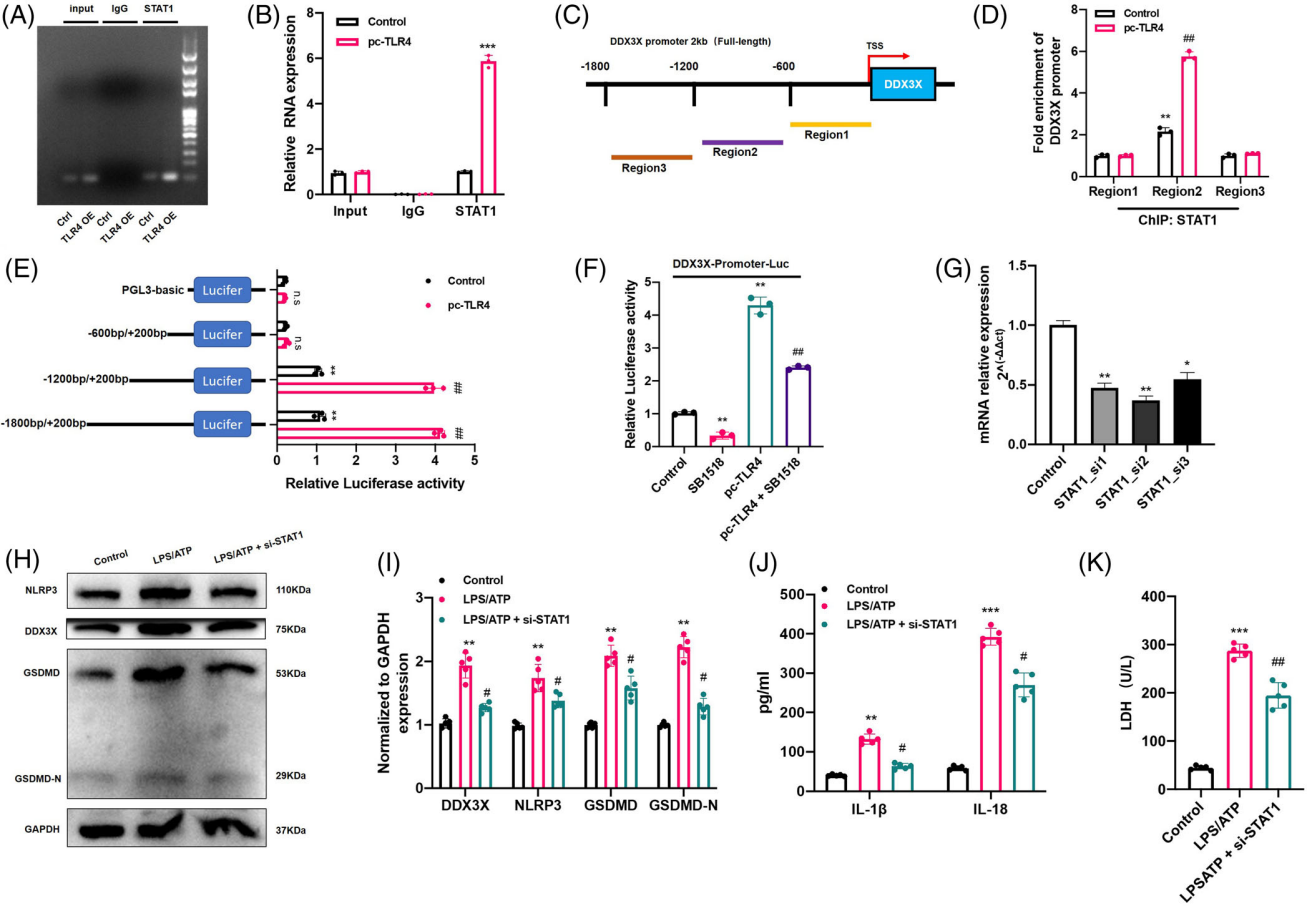
STAT1 is a transcription factor of the dead‐box helicase 3 X‐linked (DDX3X) gene. (A and B) STAT1 binds to its binding sites in the DDX3X promoter region as shown by the chromatin immunoprecipitation (ChIP) assay in BV2 cells with or without the transfection of Toll‐like receptor 4 (TLR4) overexpression plasmid (*N* = 3). (C and D) Schematic of the division of the promoter region of DDX3X and ChIP analysis between STAT1 and each region of the DDX3X promoter (*N* = 3). (E) A series of plasmids containing 5′ unidirectional deletions of the promoter region of the DDX3X gene fused in frame to the luciferase gene were transfected into microglia (*N* = 3). (F) Dual‐luciferase reporter assay of DDX3X and STAT1 with or without SB1518 and pc‐TLR4 treatment (*N* = 3). (G) The efficiency of STAT1‐siRNA knockdown was measured by real‐time quantitative polymerase chain reaction (RT‐qPCR) (*N* = 3). (H and I) Representative immunoblot and semiquantitative analysis of DDX3X, NLR family pyrin domain containing 3 (NLRP3), GSDMD and GSDMD‐N in different treated microglia (*N* = 5). (J and K) Released levels of inflammatory cytokines (interleukin [IL]‐1β and IL‐18) and lactate dehydrogenase (LDH) in the cell culture supernatant of different groups (*N* = 5). *p < .05, **p < .01, ***p < .001 versus control, ^#^ *p* < .05, ^##^ *p* < .01 versus lipopolysaccharides (LPS)/adenosine triphosphate (ATP)

Given that STAT1 is an important transcription factor involved in various diseases, we speculated that STAT1 could activate *DDX3X* gene transcription by binding to its promoter region. First, the results obtained from the Cistrome database (http://cistrome.org/db/#/) revealed a transcriptional regulatory relationship between STAT1 and DDX3X (Figure S5). Moreover, the online bioinformatics JASPAR database (http://jaspar.generge.net) was also used, and the results also indicated that there was a potential binding site between STAT1 and the DDX3X promoter region (Table S3). These results jointly indicated that STAT1 likely acted as a transcriptional activator to promote *DDX3X* expression. To confirm this, BV2 cells transfected with or without a TLR4 overexpression plasmid (pc‐TLR4) were used for the ChIP assay. The results of agarose gel electrophoresis analysis suggested that there was direct binding of STAT1 to the *DDX3X* promoter region, and *TLR4* overexpression resulted in increased binding of STAT1 to the *DDX3X* promoter (Figure 8A). The RT‐qPCR results were also similar to the DNA electrophoresis results (Figure 8B). Next, the *DDX3X* promoter region was divided into three parts: region 1 (–1800/–1200 bp), region 2 (–1200 bp/–600 bp) and region 3 (–600/0 bp). Further ChIP analysis implied that the positive regulatory binding site for STAT1 was located in region 2 (−1200/–600 bp of the *DDX3X* promoter) (Figure 8C,D). In addition, a series of luciferase assays further elucidated the results of ChIP (Figure 8E). Moreover, the JAK2 inhibitor SB1518 obviously decreased luciferase activity. On the other hand, pc‐TLR4 significantly increased luciferase activity, which was suppressed by SB1518 treatment (Figure 8F). Consistently, as shown in Figure 8G–K, *STAT1* siRNA markedly reduced *DDX3X* expression and inhibited NLRP3 inflammasome activation and microglial pyroptosis. Additionally, in vivo, compared with the SCI group, WB results also showed that the protein expression levels of DDX3X and pyroptosis‐associated molecules (NLRP3, GSDMD, GSDMD‐N, cleaved CASP‐1) were significantly inhibited in the SCI + fludarabine (a STAT1 inhibitor) group (Figure S5). From these results, we could safely conclude that TLR4 activated NLRP3 inflammasomes and pyroptosis by upregulating *DDX3X* in a JAK2/STAT1‐dependent manner.

### 3.8 TLR4/BGN interaction promoted STAT1/DDX3X/NLRP3 axis‐mediated pyroptosis in LPS/ATP induced BV2 microglial cells

**FIGURE 9:**
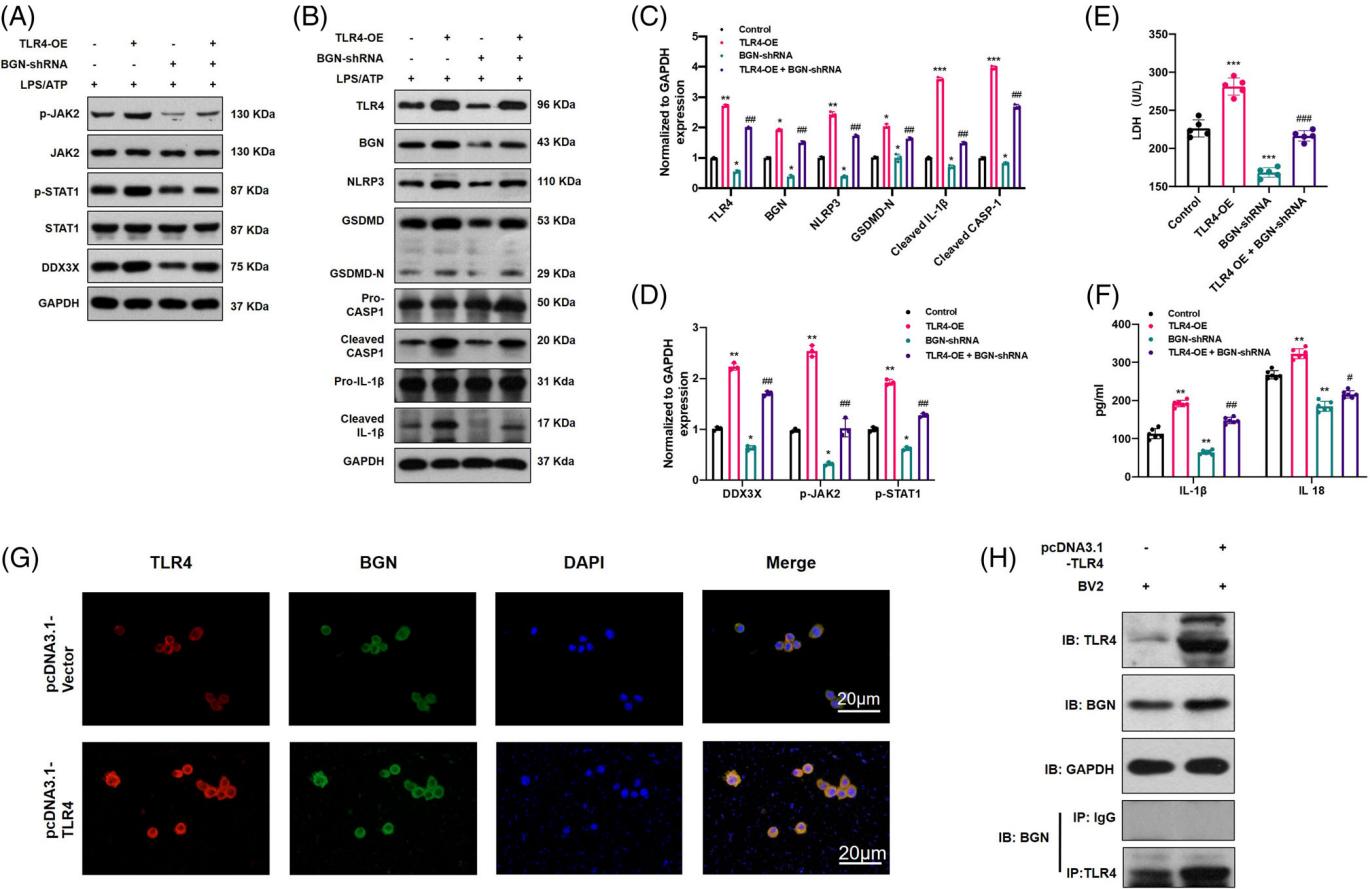
Toll‐like receptor 4 (TLR4) promoted STAT1/dead‐box helicase 3 X‐linked (DDX3X)/NLR family pyrin domain containing 3 (NLRP3)‐mediated microglial pyroptosis by interacting with biglycan (BGN). (A–D) Representative immunoblot of TLR4, BGN, DDX3X, P‐JAK2/JAK2, P‐STAT1/STAT1 and pyroptosis‐related genes and semiquantitative analysis in each group (*N* = 3). *p < .05, **p < .01, ***p < .001 versus control, ^##^ *p* < .01 versus TLR4‐OE. (E and F) The levels of inflammatory factors (interleukin [IL]‐1β and IL‐18) （*N* = 6） and lactate dehydrogenase (LDH) in the cell culture supernatant of each group (*N* = 5). **p < .01, ***p < .001 versus control, ^#^ *p* < .05, ^##^ *p* < .01, ^###^ *p* < .01 versus TLR4‐OE. (G) Representative immunofluorescence images of TLR4 and BGN colocalisation with or without TLR4 overexpression. (H) The results of coimmunoprecipitation (Co‐IP) analysis of TLR4 and BGN with or without TLR4 overexpression

BGN, which was reported to activate NLRP3 inflammasomes via TLR4, was also found to be one of the upregulated DEPs after SCI (Figure 4C). The WB results confirmed that the upregulation of BGN in LPS/ATP‐induced microglia was dependent on TLR4 (Figure S6). To investigate the role of BGN in TLR4‐induced DDX3X/NLRP3 signalling in more detail, we downregulated the expression of *BGN* by transfecting a *BGN*‐specific shRNA plasmid into LPS/ATP induced BV2 cells. First of all, it could be seen that *TLR4* overexpression further upregulated *DDX3X* expression and aggravated BV2 pyroptosis (Figure 9A–F). Moreover, TLR4 also promoted both the mRNA and protein levels of BGN (Figure 9B,C,G). On the other hand, *BGN* shRNA obviously reduced the activation of the JAK2/STAT1/DDX3X axis and suppressed NLRP3 inflammasome‐mediated pyroptosis in LPS/ATP‐stimulated BV2 microglia (Figure 9A–F). As expected, similar results were obtained by IF staining of NLRP3 (Figure S6). Excitingly, *BGN* knockdown, in turn, decreased *TLR4* expression (Figure 9B,C). In addition, ROS production in LPS/ATP‐induced BV2 cells was significantly suppressed in the presence of BGN shRNA (Figure S6). Furthermore, the results of double‐stained IF analysis revealed the colocalisation of TLR4 and BGN in BV2 cells (Figure 9G). Subsequently, a co‐immunoprecipitation (Co‐IP) assay was also performed, which further validated the direct protein–protein interaction between TLR4 and BGN (Figure 9H). To gain further mechanistic insight, we mapped the protein domains that mediate the TLR4–BGN interaction by Co‐IP assays using a series of truncated TLR4 and BGN proteins. The results also demonstrated the possibility of multiple binding sites between TLR4 and BGN (Figure S6). Collectively, these results implied that BGN and TLR4 could promote the expression of each other and jointly promoted the STAT1/DDX3X/NLRP3 axis and BV2 microglial pyroptosis.

## 4 DISCUSSION

Traumatic SCI is a major kind of CNS injury with poor prognosis, significant morbidity and high mortality. Restoration of neurological function after injury has been a huge challenge for both patients with SCI and clinicians. Globally, 250 000–500 000 people suffer from SCI, and more than 15 000 new cases are reported annually in the USA alone.^27^, ^28^ The health and economic burden caused by SCI has made it an urgent issue in the field of neuroscience. In this study, we identified that *DDX3X* is upregulated after SCI and mediates TLR4‐induced NLRP3 inflammasome activation and microglial pyroptosis using in vivo and in vitro molecular biology experiments. DDX3X may thus be a potential target to regulate microglial pyroptosis to alleviate neuroinflammation after SCI.

Neuroinflammation is generally regarded as one of the major factors that leads to further tissue damage and neurodegeneration during the secondary phase of SCI. It is one of the mechanisms that forms part of the innate immune response, which is initiated by the activation of resident innate immune cells and the infiltration of peripheral immune cells. Of these, microglia, as the main resident immune cells in the CNS, play a central role in neuroinflammation initiation and activation after CNS injury such as SCI. For example, Wu and coworkers found that systemic administration of riluzole could facilitate motor function recovery after SCI by promoting microglial M2 polarisation and inhibiting M1 polarisation. Moreover, in our previous study,^21^ we reported that CD73 attenuated neuroinflammation by promoting microglial M2 polarisation after SCI. Recently, microglial pyroptosis was identified as another significant source of neuroinflammation in various neurological diseases. For instance, Chang et al.^29^ showed that microglial pyroptosis plays a critical role in post‐cardiac arrest brain injury. Furthermore, Liu et al.^12^ reported that kaempferol primarily inhibits microglial‐mediated pyroptosis as the underlying mechanism when used to treat SCI. In line with these previous studies, we reported that pyroptosis‐associated genes (*NLRP3*, *GSDMD*, *GSDMD‐N*, *cleaved CASP‐1*, *cleaved IL‐1β*) increased significantly after SCI in vivo and in vitro. We also found that TLR4 deficiency alleviated microglial pyroptosis and promoted the recovery of neurological functions in vivo. Over the years, TLR4, as an important pattern recognition receptor for danger‐ and pathogen‐associated molecular patterns (DAMPs and PAMPs), has been acknowledged to play a vital role in initiating pyroptosis.^30^ Yuan et al.^31^ reported that cucurbitacin B exerts its antitumour activities by directly binding to TLR4 and subsequently activating pyroptosis, while silencing *TLR4* inhibited cucurbitacin B‐induced pyroptosis. In CNS injury, various DAMPs (including HMGB1, Mrp‐8, Mrp‐14) and inflammatory cytokines (such as interleukins) secreted by injured tissue have been reported to activate TLR4 in previous studies, which may also be triggering factors of TLR4 upregulation after SCI.^32^, ^33^ However, we did not detect the expression level of these DAMPs, which is one of the limitations of this study.

The activation of inflammasomes is a key step for the initiation of pyroptosis, which subsequently triggers the production of GSDMD‐N as well as the maturation of IL‐1β and IL‐18 by cleaved caspase‐1. Diverse molecular mechanisms of regulating NLRP3 inflammasome activation have been reported. For example, Lin et al.^34^ found that zinc exerts neuroprotection by inducing ubiquitination and degradation of NLRP3 inflammasomes in a spinal contusion injury model. Barry et al.^35^ also reported that the SUMOylation of NLRP3 inhibits inflammasome activation. Furthermore, noncoding RNAs, such as microRNAs and lncRNAs, have also been thought to be involved in the regulation of NLRP3 inflammasome activation.^36^, ^37^, ^38^ Nevertheless, the specific regulatory mechanism of NLRP3 inflammasomes and pyroptosis in SCI has rarely been reported. Therefore, in this study, we used high‐throughput quantitative proteomics to search for potential targets.

Among the DEPs identified using TMT‐based quantitative proteomics, we found that *DDX3X* decreased significantly in the SCI‐TLR4‐KO group. DDX3X reportedly participates in RNA metabolism, cell apoptosis, cell cycle and innate immunity in various diseases, including cancer, virus infection and inflammation.^39^, ^40^ Importantly, Samir and coworkers showed that DDX3X could act as a positive regulator of the NLRP3 inflammasome. Furthermore, the regulation of NLRP3 inflammasome activation by DDX3X has repeatedly been demonstrated by a large number of studies.^41^, ^42^ However, there has not been a report describing the relationship between DDX3X and SCI. Our in vivo and in vitro results jointly demonstrated for the first time that *DDX3X* expression increased after SCI but decreased when TLR4 was deficient. Moreover, *DDX3X* siRNA alleviated the NLRP3 inflammasome and microglial pyroptosis in vitro, while the upregulated *DDX3X* effectively reversed the anti‐pyroptotic effect of TLR4 deficiency. These results agreed with previously published literature^43^, ^44^ and suggest DDX3X as a potential target to regulate NLRP3 inflammasome‐mediated microglial pyroptosis after SCI.

Increasing evidence indicates that the JAK/STAT signalling pathway participates in many neuroinflammatory diseases by regulating many biological processes, including cell apoptosis, differentiation and proliferation.^45^, ^46^, ^47^ Gao et al.^48^ also reported that the JAK/STAT pathway mediated the inhibitory effect of autophagy on pyroptosis in a TBI model. Herein, we found that the JAK2/STAT1 pathway is activated and that a JAK2 inhibitor (SB1518) alleviates DDX3X/NLRP3‐mediated microglial pyroptosis in vivo and in vitro. In addition, luciferase experiments and ChIP assays together confirmed that STAT1 is a transcriptional activator of DDX3X, which further strengthens the important role of the JAK2/STAT1 pathway in the TLR4‐induced DDX3X/NLRP3 axis after SCI.

BGN is a small leucine‐rich proteoglycan that can function as an endogenous activator of TLR2/4 to initiate inflammation and tissue damage.^49^, ^50^ Of note, Babelova et al.^51^ identified BGN as a DAMP to activate NLRP3 inflammasomes via Toll‐like receptors and P2X receptors in macrophages. We detected that *BGN* shRNA decreased the levels of p‐JAK2, p‐STAT1, *DDX3X* and pyroptosis‐related genes, which reversed the pro‐pyroptotic effect of TLR4 in LPS/ATP‐induced microglia. Moreover, intracellular ROS, as one of the most common stimuli of NLRP3 inflammasome activation, have also been widely reported to be involved in the pathological process of SCI.^52^, ^53^, ^54^ Notably, ROS production in pyroptotic BV2 cells was greatly inhibited by *BGN* shRNA. In addition, ROS was also reported to mediate the activation of JAK/STATs pathway.^55^, ^56^, ^57^ Therefore, ROS and oxidative stress may also participate in BGN‐mediated NLRP3 inflammasome activation and microglial pyroptosis after SCI in mice. Interestingly, *TLR4* siRNA significantly decreased BGN protein levels in LPS/ATP‐treated BV2 cells. Also considering the results of IF and Co‐IP, it would appear that there is a direct interaction between BGN and TLR4, which jointly promotes pyroptosis after SCI.

Our study is not without limitations. First, we used the BV2 microglial cell line in vitro, which is the most widely used cell line in previous research for its similarity with primary microglia in vitro.^58^, ^59^ However, it cannot fully reproduce the characteristics of primary microglial cells. In addition, although the present study focused on microglial pyroptosis‐mediated neuroinflammation after SCI, many other mechanisms could also have been investigated, including myelin phagocytosis, axonal damage, astrogliosis, etc.^60^, ^61^ Another limitation is that the role of BGN in the STAT1/DDX3X/NLRP3 axis was not fully researched in vivo. The specific regulatory role of DDX3X on NLRP3 inflammasome activation was not explored in this study, although some potential mechanisms, such as DDX3X phosphorylation induced by AKT, were investigated.^41^ Therefore, further experimental validation of the present results is required.

## 5 CONCLUSION

**FIGURE 10:**
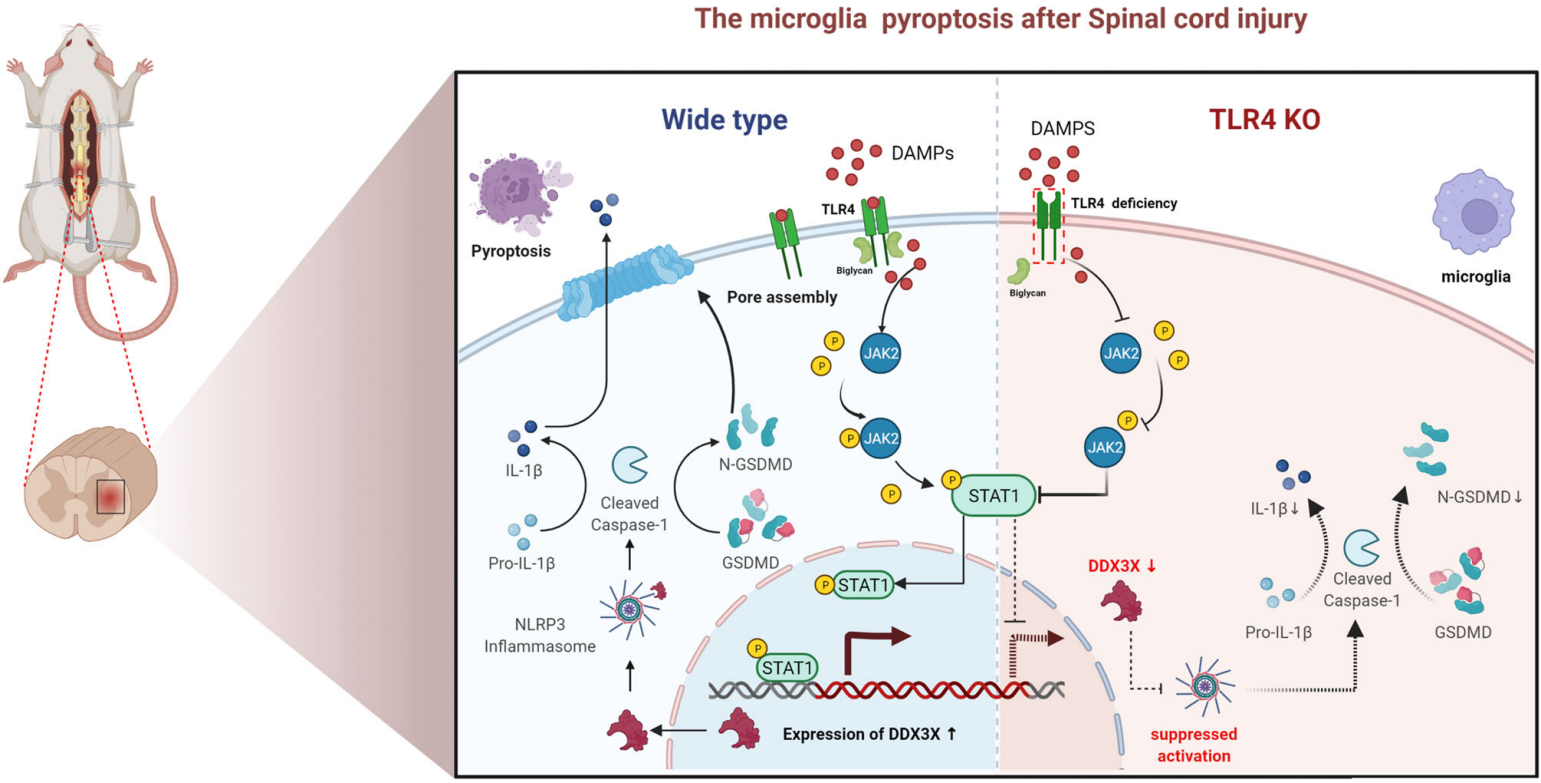
Schematic illustration describing the underlying mechanism of Toll‐like receptor 4 (TLR4)‐mediated microglial pyroptosis after spinal cord injury (SCI). After injury, overexpressed TLR4 recognises danger‐associated molecular patterns (DAMPs) and interacts with biglycan (BGN) to activate the JAK2/STAT1 pathway. Furthermore, STAT1, as a transcriptional activator, upregulates the expression of dead‐box helicase 3 X‐linked (DDX3X) and subsequently aggravates NLR family pyrin domain containing 3 (NLRP3) inflammasome activation and microglial pyroptosis. This figure was drawn with Biorender (www.biorender.com)

Our study revealed that TLR4 promotes microglial pyroptosis by activating the STAT1/DDX3X/NLRP3 signalling axis after SCI in vivo and in vitro. We also found that BGN is an important molecule that mediates the pro‐pyroptotic role of TLR4 in vitro. These preliminary results could help us to better understand the pyroptotic mechanism underlying SCI and provide some experimental evidence to identify potential therapeutic targets for SCI (Figure 10).

## CONFLICTS OF INTEREST

The authors declare that they have no conflicts of interest.

## Supporting information

Supporting Information

Supporting Information

Supporting Information

Supporting Information

Supporting Information

Supporting Information

Supporting Information

## Data Availability

The data that support the findings of this study are available from the corresponding author upon reasonable request.
