# Supplementary figures and images for: TLR4 aggravates microglial pyroptosis by promoting DDX3X‐mediated NLRP3 inflammasome activation via JAK2/STAT1 pathway after spinal cord injury
Source: Clin Transl Med. 2022 Jun 12;12(6):e894. doi: 10.1002/ctm2.894 (PMC9189419; doi:10.1002/ctm2.894)

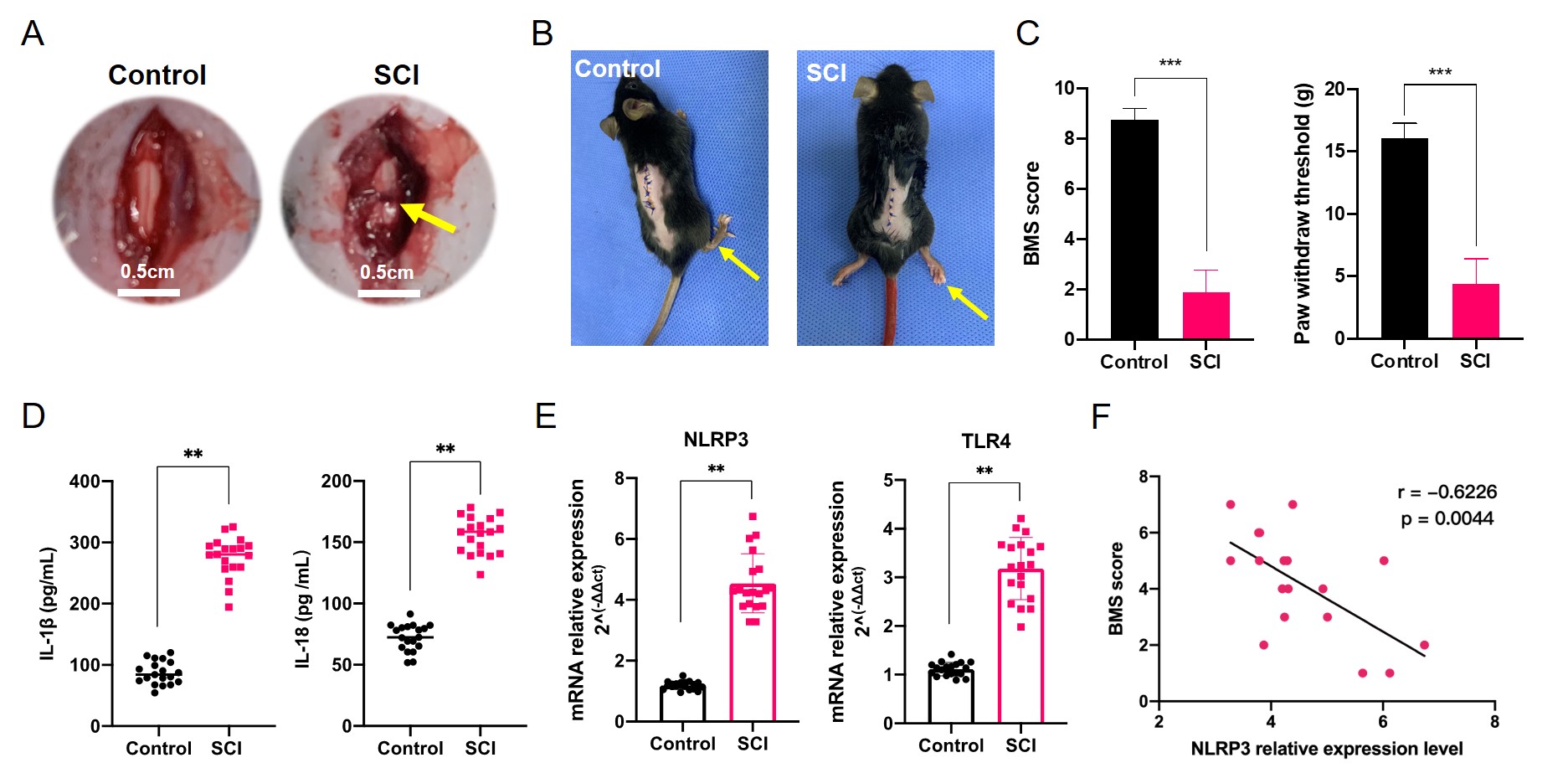

Supplement: Supplementary file 1 — Supporting Information [file CTM2-12-e894-s001.jpg]

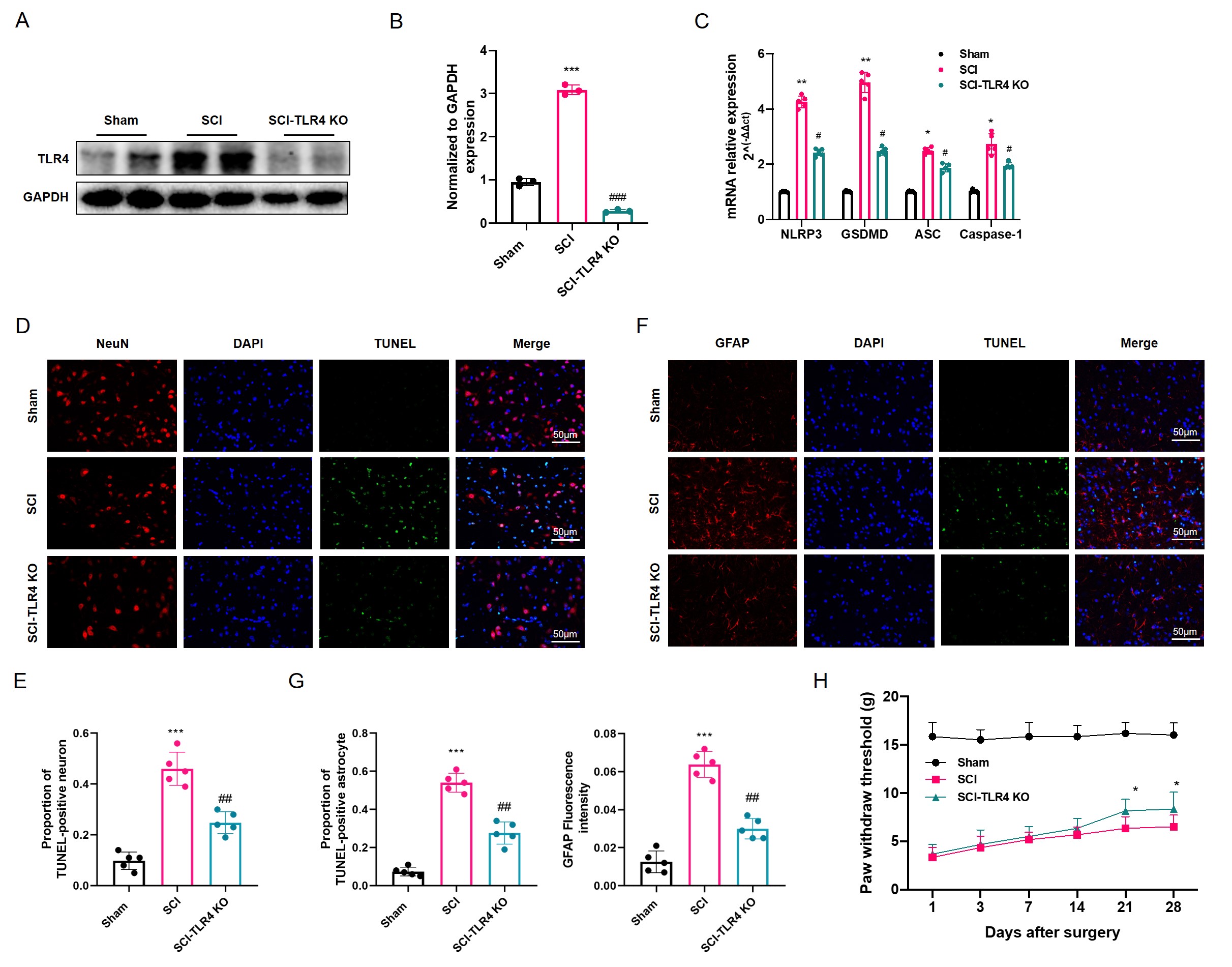

Supplement: Supplementary file 2 — Supporting Information [file CTM2-12-e894-s002.jpg]

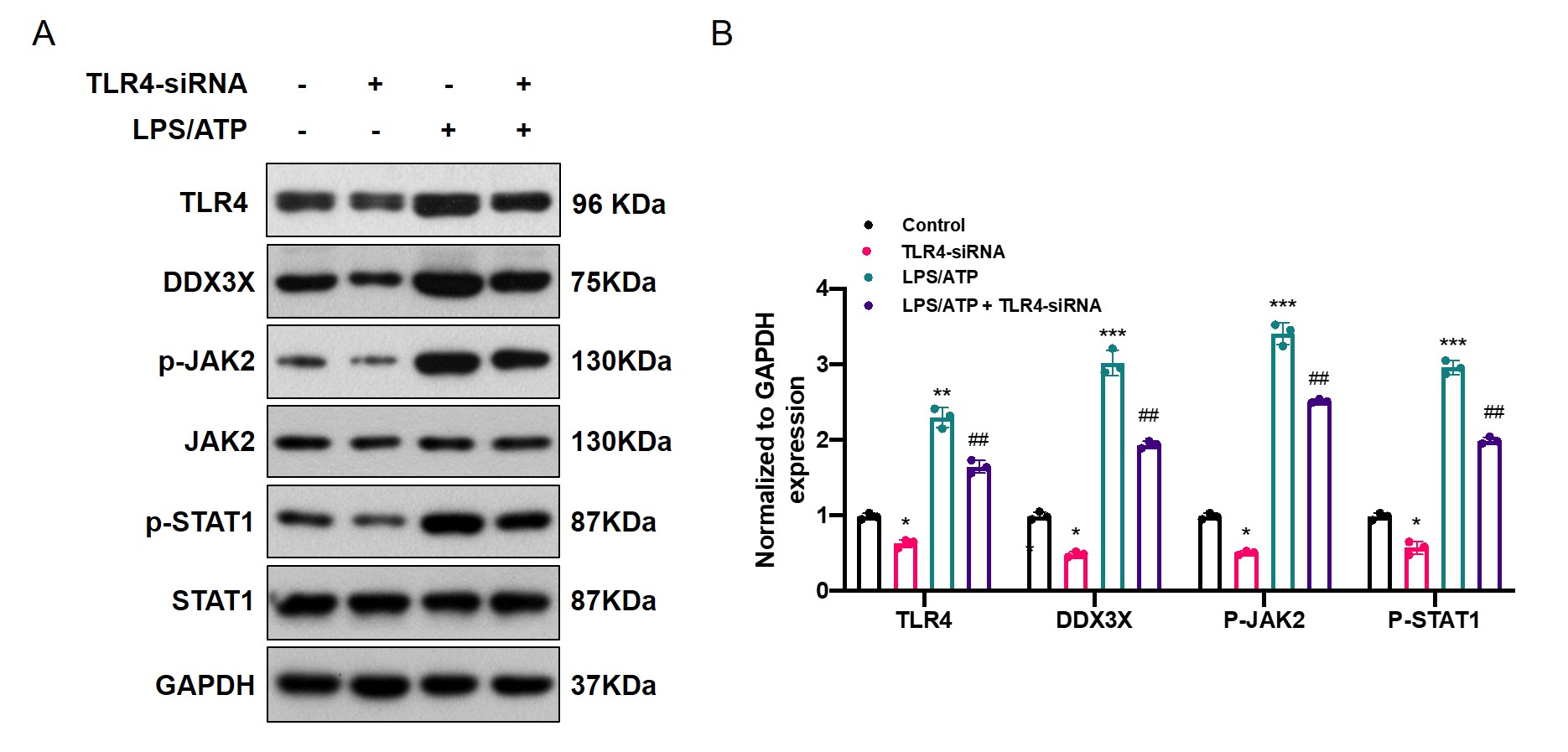

Supplement: Supplementary file 3 — Supporting Information [file CTM2-12-e894-s003.jpg]

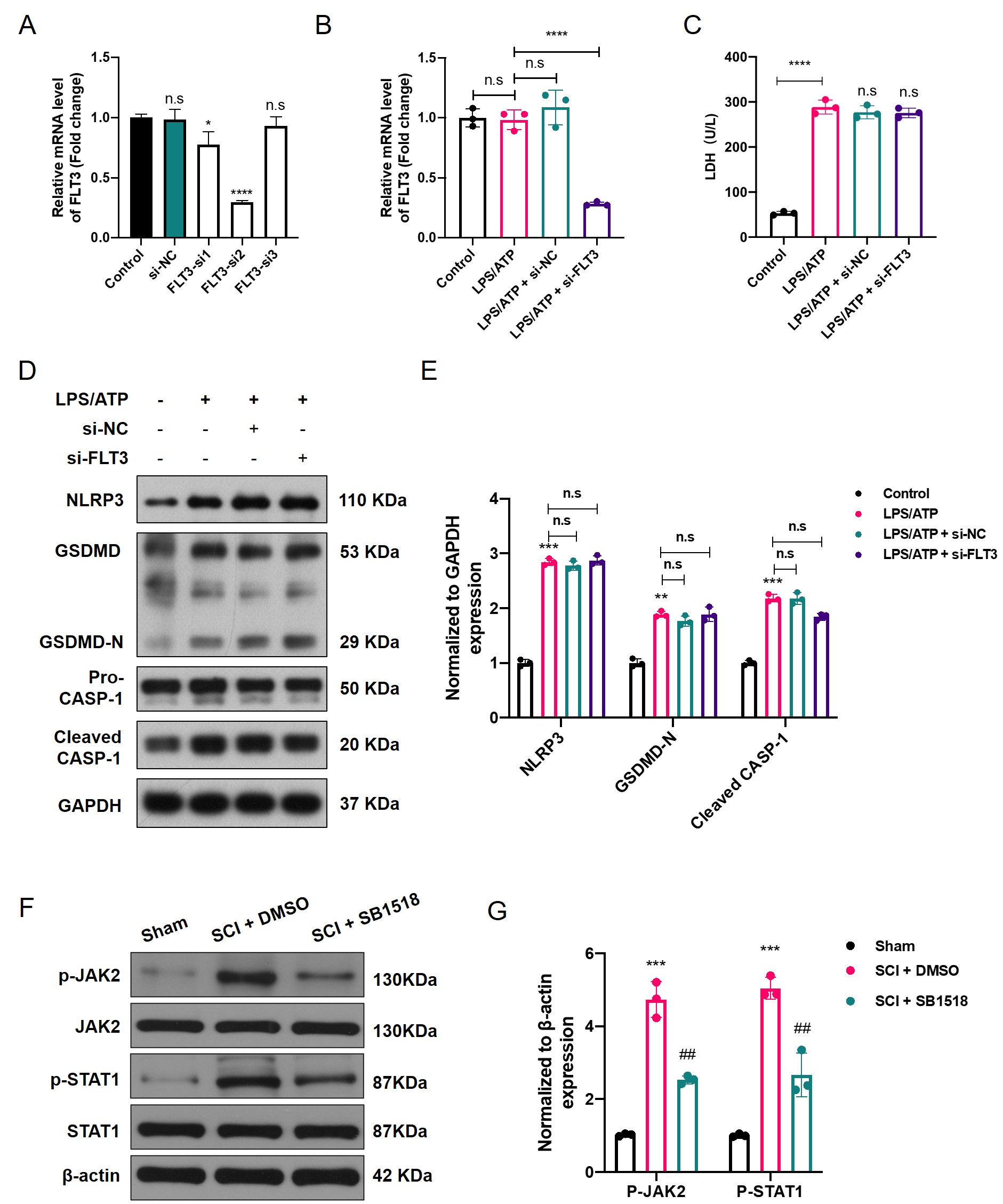

Supplement: Supplementary file 4 — Supporting Information [file CTM2-12-e894-s006.jpg]

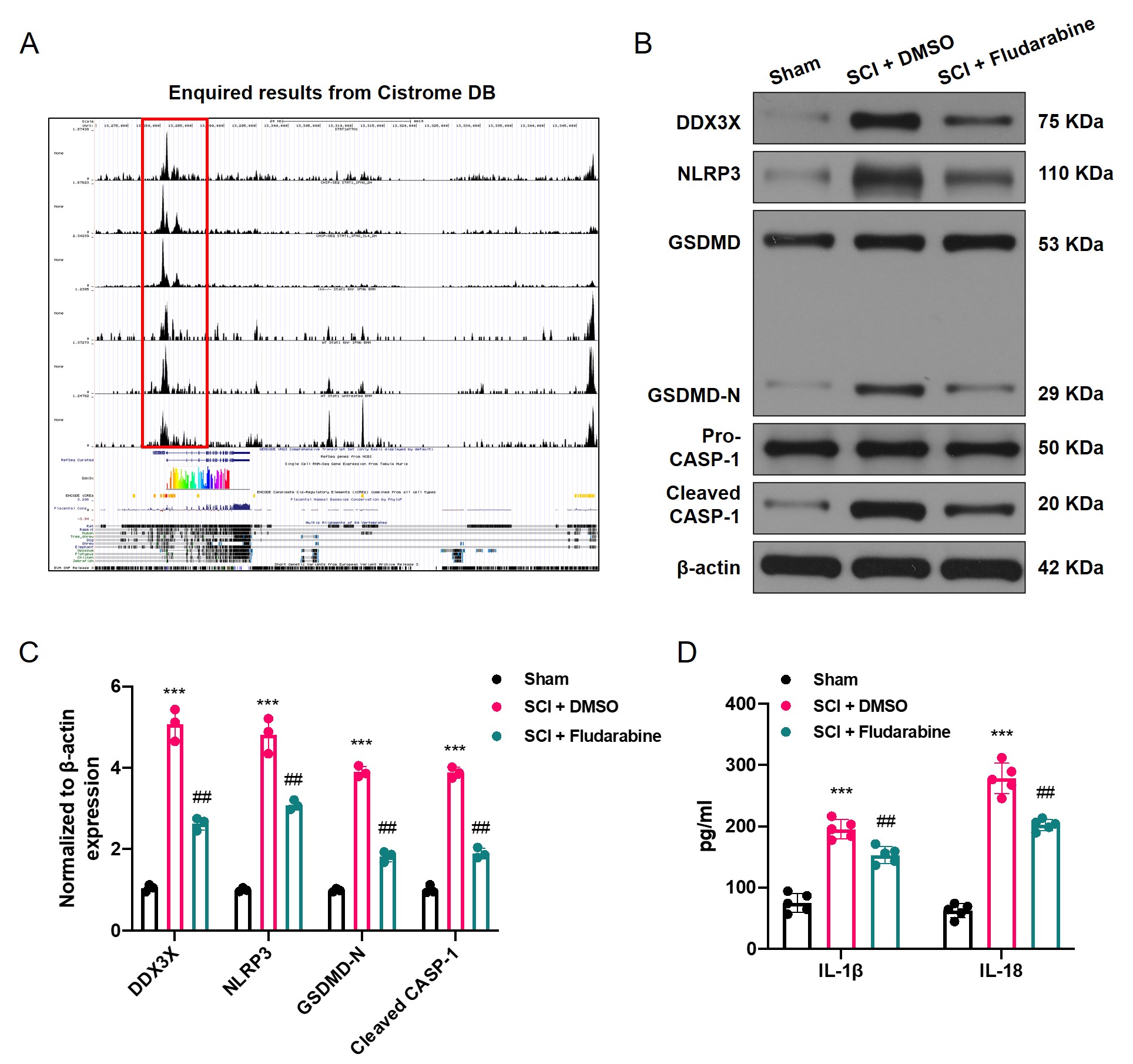

Supplement: Supplementary file 5 — Supporting Information [file CTM2-12-e894-s004.jpg]

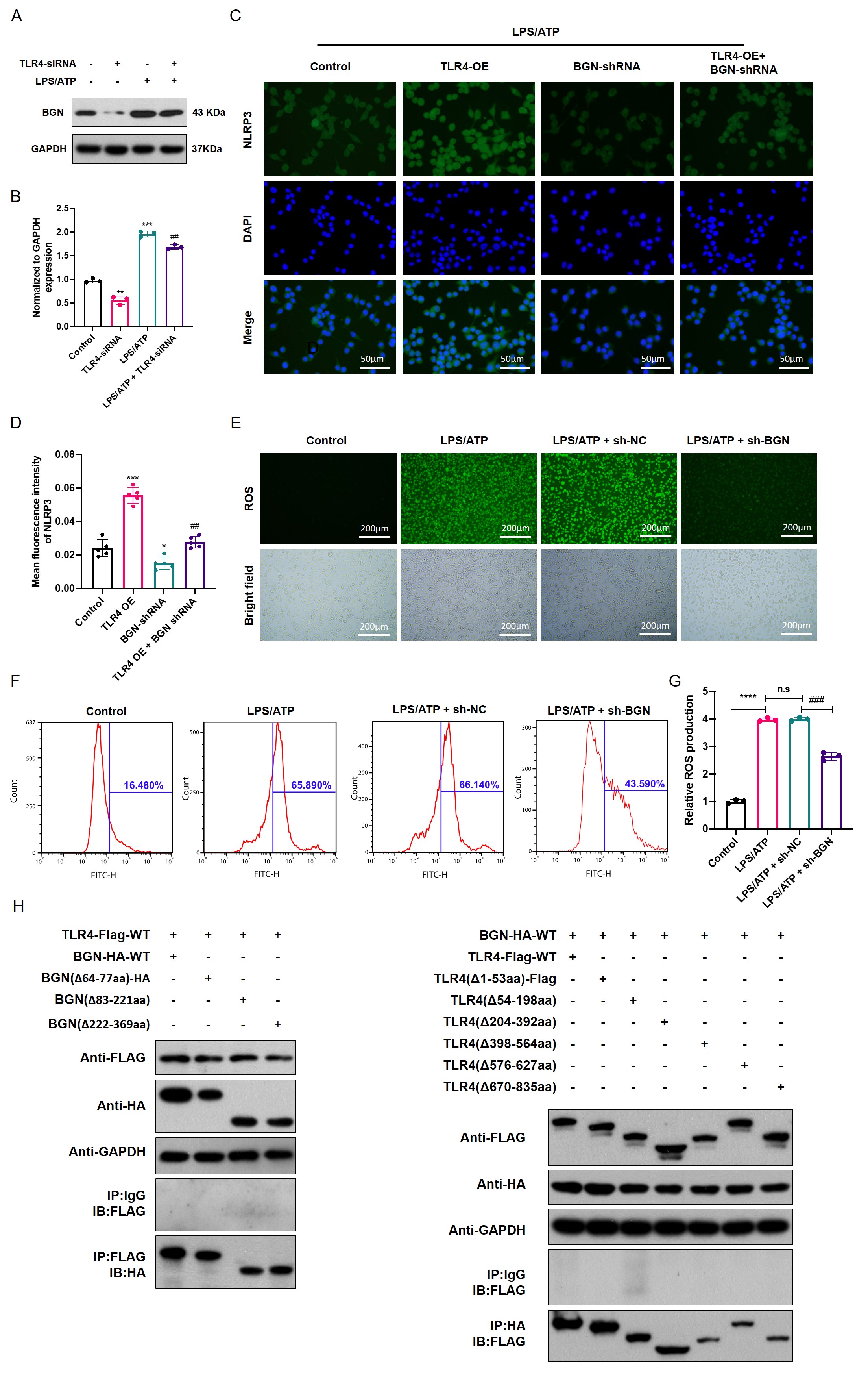

Supplement: Supplementary file 6 — Supporting Information [file CTM2-12-e894-s007.jpg]
