# Supplementary material for: TLR4 aggravates microglial pyroptosis by promoting DDX3X‐mediated NLRP3 inflammasome activation via JAK2/STAT1 pathway after spinal cord injury
Source: Clin Transl Med. 2022 Jun 12;12(6):e894. doi: 10.1002/ctm2.894 (PMC9189419; doi:10.1002/ctm2.894)
Supplement: Supplementary file 7 — Supporting Information [file CTM2-12-e894-s005.docx]

**TLR4 aggravates microglia pyroptosis by promoting DDX3X-mediated NLRP3 inflammasome activation via JAK2/STAT1 pathway after spinal cord injury**

*Jin Wang^‡,a^, Fan Zhang^‡,a^, Haocheng Xu^a^, Haiyuan Yang^a^, Minghao Shao^a^, Shun Xu^*,b^, Feizhou Lyu^*,a,b^*

*^a^* Department of Orthopedics, Huashan Hospital, Fudan University, Shanghai 200040, P. R. China.

*^b^* Department of Orthopedics, Shanghai Fifth People’s Hospital, Fudan University, Shanghai 200240, P. R. China.

*** Author to whom correspondence should be addressed F. Z. Lyu (E-mail: [lyufzsubmit@163.com](mailto:lyufzsubmit@163.com)) and S. Xu (E-mail: xshun16@163.com)

*‡* These authors equally contributed to this work.

**Supplementary Tables**

**Table S1.** The siRNA/shRNA sequences used in this study.

| **Gene** | **Sense sequence（5'-3'）** | **Antisense-sequence（3'-5'）** |
| --- | --- | --- |
| Si-control | UUCUCCGAACGUGUCACGU | ACGUGACACGUUCGGAGAA |
| mTLR4 si-1 | CCGUUGGUGUAUCUUUGAAUA | UAUUCAAAGAUACACCAACGG |
| mTLR4 si-2 | CCUGUAAGUUACCUGCAUAUU | AAUAUGCAGGUAACUUACAGG |
| mTLR4 si-3 | GGUUGCUGUUCUUAUUCUGAU | AUCAGAAUAAGAACAGCAACC |
| mDDX3X si-1 | GCACUGUUCUCAAAGUUAAUG | UUAACUUUGAGAACAGUGCUU |
| mDDX3X si-2 | CAAAGUUAAUGCAAGUAAAUA | UUUACUUGCAUUAACUUUGAG |
| mDDX3X si-3 | CAGGUGUGAUACAACUUAACA | UUAAGUUGUAUCACACCUGUG |
| mSTAT1 si-1 | CGAGAGCUGUCUAGGUUAAC | GUUAACCUAGACAGCUCUCG |
| mSTAT1 si-2 | GGGCAUCAUGCAUCUUACU | AGUAAGAUGCAUGAUGCCC |
| mSTAT1 si-3 | CCAUGAGACUACAGUCAAAUG | UUUGACUGUAGUCUCAUGGGA |
| mBGN sh-1 | GGACTTCACCTTGGATGATGGTTCAAGAGACCATCATCCAAGGTGAAGTCCTTTTTT | AAAAAAGGACTTCACCTTGGATGATGGTCTCTTGAACCATCATCCAAGGTGAAGTCC |
| mBGN sh-2 | GGAGAACAGTGGCTTTGAACCTTCAAGAGAGGTTCAAAGCCACTGTTCTCCTTTTTT | AAAAAAGGAGAACAGTGGCTTTGAACCTCTCTTGAAGGTTCAAAGCCACTGTTCTCC |
| mBGN sh-3 | GGTTGGGCTTAGGTCACAATCTTCAAGAGAGATTGTGACCTAAGCCCAACCTTTTTT | AAAAAAGGTTGGGCTTAGGTCACAATCTCTCTTGAAGATTGTGACCTAAGCCCAACC |
| Scramble shRNA | TTCTCCGAACGTGTCACGTTTCAAGAGAACGTGACACGTTCGGAGAATTTTTT | AAAAAATTCTCCGAACGTGTCACGTTCTCTTGAAACGTGACACGTTCGGAGAA |

**Table S2.** Sequences of primers for chromatin-immunoprecipitation (ChIP)

| **Primer** | **Sequence (5'-3)** | **Product length (bp)** |
| --- | --- | --- |
| mDDX3X-promoter-F1 | AAACCACCACAGGACGATAGG | 142 |
| mDDX3X-promoter-R1 | TGGAATTGATCTAGCACGCCT |  |
| mDDX3X-promoter-F2 | AATCGGCCCGTTTGATGACA | 94 |
| mDDX3X-promoter-R2 | TGTGTCTCCCTGACCGAGAT |  |
| mDDX3X-promoter-F3 | CACGGTCACGTGATCCCACT | 78 |
| mDDX3X-promoter-R3 | GATGATGCAGTGGGCTTTGTC |  |

**Table S3.** JASPAR Prediction results

| **Matrix ID** | **Name** | **Score** | **Relative Score** | **Sequence ID** | **Start** | **End** | **Strand** | **Predicted sequence** |
| --- | --- | --- | --- | --- | --- | --- | --- | --- |
| 10676 | MA0137.3.STAT1 | 10.199511 | 0.899781811601722 | NC_000086.8:13145261-13147360 | 1101 | 1111 | + | GTGCCAGGAAT |
| 10676 | MA0137.3.STAT1 | 6.1640935 | 0.844643115593578 | NC_000086.8:13145261-13147360 | 96 | 106 | + | GTTCTAGCAAC |
| 9383 | MA0137.2.STAT1 | 9.311078 | 0.831954723339962 | NC_000086.8:13145261-13147360 | 1431 | 1445 | + | CCATTCCCAGCACTA |
| 10676 | MA0137.3.STAT1 | 3.5264452 | 0.808603101057811 | NC_000086.8:13145261-13147360 | 1603 | 1613 | + | GTGCTAGAAAG |
| 9383 | MA0137.2.STAT1 | 7.4841437 | 0.802461716897239 | NC_000086.8:13145261-13147360 | 2070 | 2084 | + | GATTTCTCGGTACTC |
| 10676 | MA0137.3.STAT1 | 2.9072356 | 0.800142412793916 | NC_000086.8:13145261-13147360 | 2084 | 2094 | + | CTTCAGGGATG |

**Supplementary Figures**

**Figure s1.** The larger view of the HE and Nissl images in Figure 2K. (According to comments of Reviewer#3).


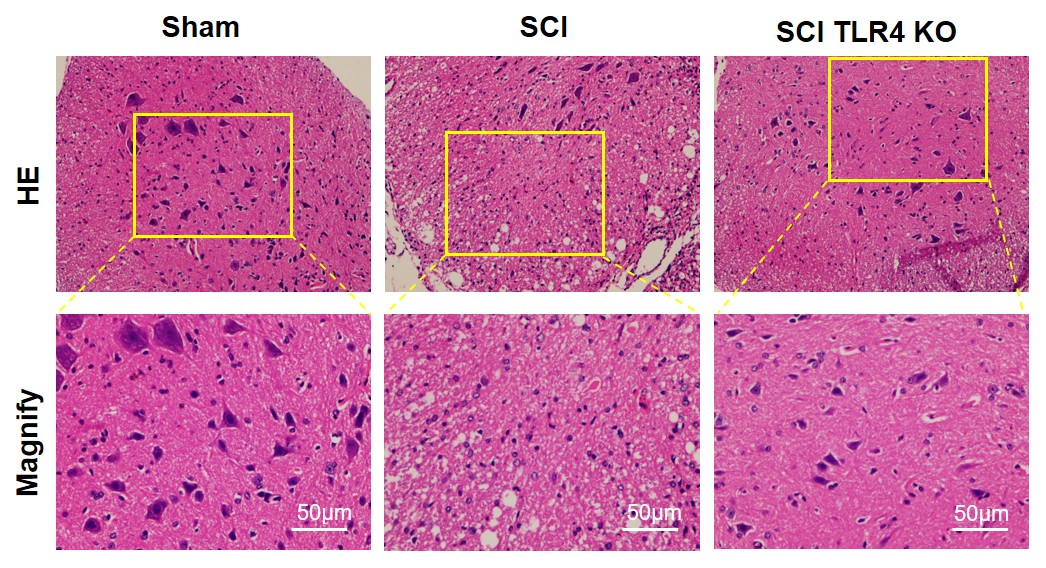
.
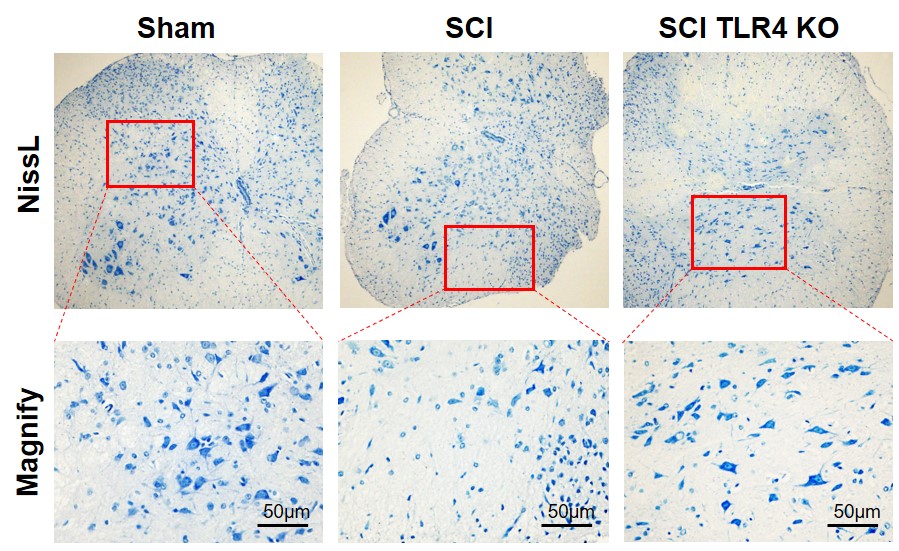


**Figure s2.** The larger view of the IF images in Figure 4J. (According to comments of Reviewer#3).


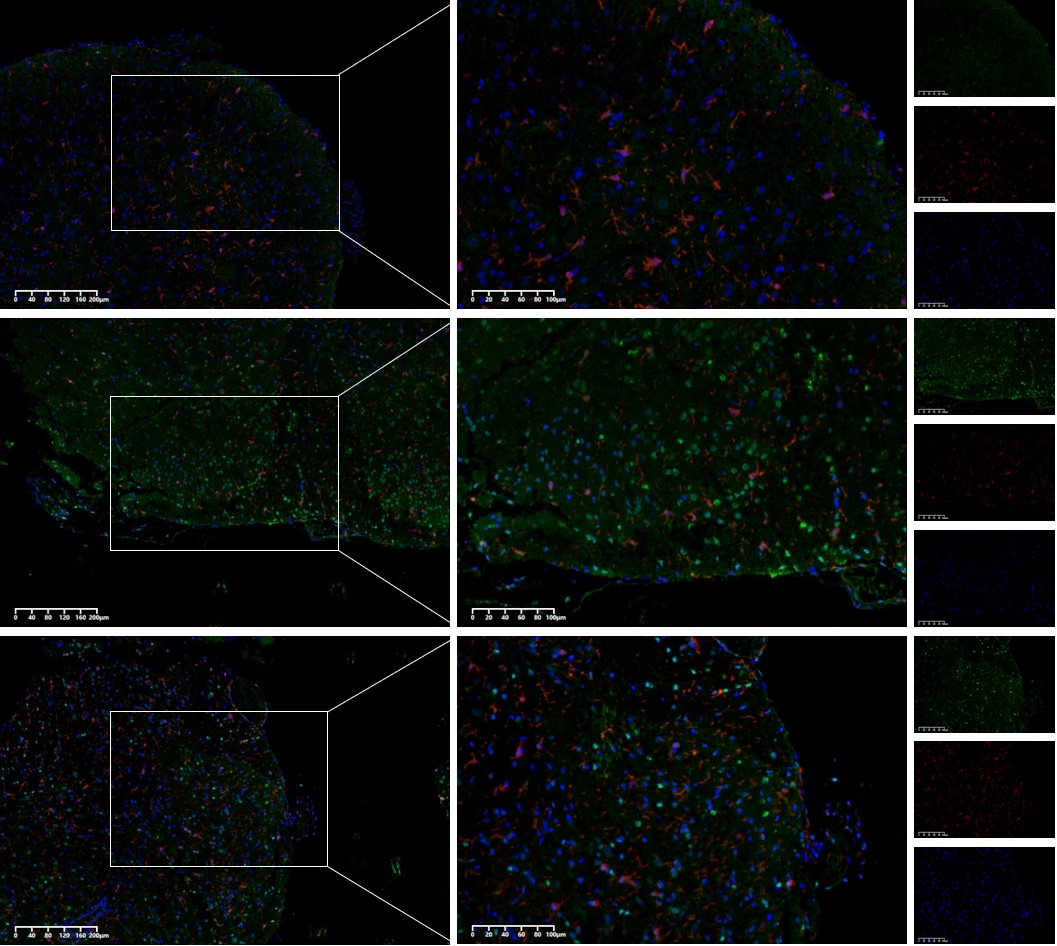


**Figure s3**. The larger view of the IHC images in Figure 4H, Fig.6G, Fig.7H. (According to comments of Reviewer#3).

Fig.4H


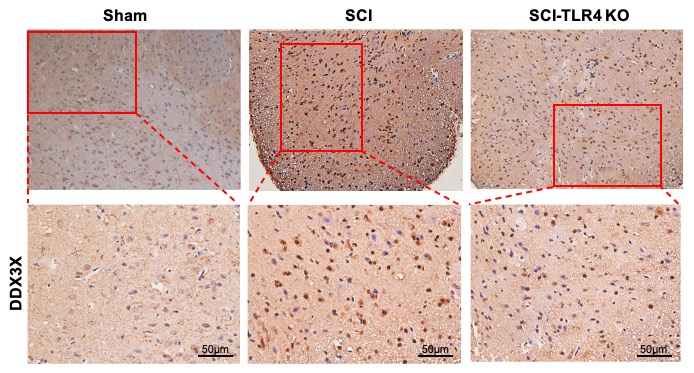


Fig.6G


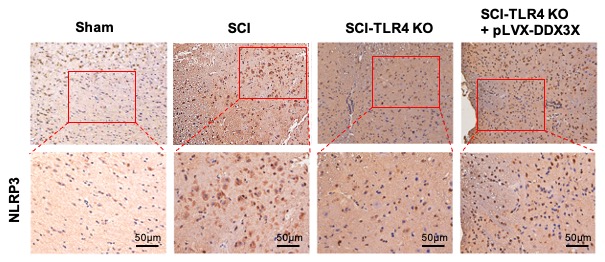


**
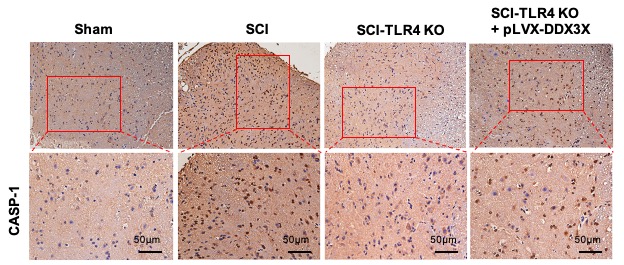
**

Fig.7H


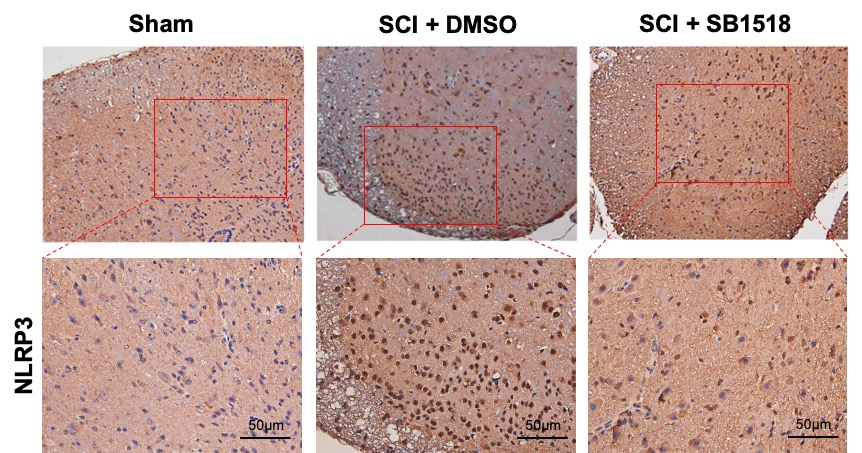


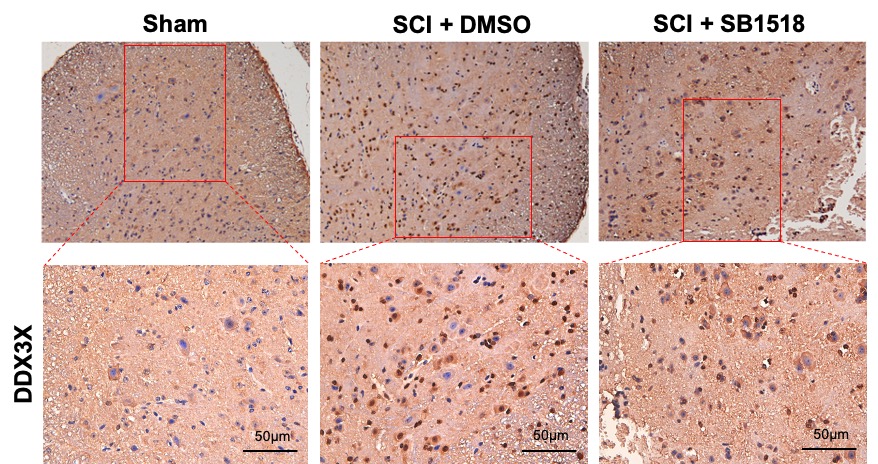


**Construction of Lentiviral Overexpression Vector**

| Gene | DDX3X |
| --- | --- |
| Method | The DDX3X gene sequence of mouse origin was synthesized, and EcoRI-XbaI restriction sites (Red) were added at both ends. The resulting gene fragment was blunt-ended cloned into the PUC57 vector. |
| DDX3X  sequence | GGATCCatgagtcatgtggcagtggaaaatgcgctcgggctggaccagcagtttgctggcctagacctgaactcttcagataatcagagtggaggaagtacagcaagcaaagggcgttatatcccacctcatttaaggaacagagaagctactaaaggattctatgacaaagacagttcagggtggagttctagtaaagataaggatgcatacagcagttttggatcacggggtgattcaagagggaagtctagcttctttggagatcgtggaagtggatcaaggggaaggtttgatgatcgtggacggggagactatgatggcattggtggccgtggagatagaagtggctttggcaaatttgaaagaggtggaaatagtcgctggtgtgacaaatcagatgaagatgactggtcaaagccactcccaccaagtgaacgattggaacaggaactcttttctggaggcaatactgggattaactttgagaaatatgatgacattccagtcgaagcaacaggcaacaactgtcctccacacattgaaagtttcagtgatgtcgagatgggagaaattattatgggaaacattgagcttactcgttatactcgcccaactccagtgcagaagcatgctattcctattatcaaagagaaaagagacttgatggcttgtgctcaaacaggctctggaaaaactgcagcatttctcttgcccatcttgagtcagatctatgctgatggtccaggagaagctctgagggctatgaaggaaaatggaagatatggccgtcgtaaacagtatccaatctctttggtactggcaccaacgagagaattggcagtgcagatctatgaggaagccagaaaattctcataccgatctagagtccgtccttgcgtggtttatggtggtgctgaaattggccagcagattcgagacttagaacgtggatgccacttgttagtagccactccaggacgtctagtggatatgatggagagagggaagatcgggttagacttctgcaaatacctggtgttagatgaagctgaccggatgttagatatggggtttgaacctcagatacgaagaatagttgaacaagacactatgcctccaaaaggtgtccgccacactatgatgtttagtgctacttttcctaaggaaatacagatgctggcccgtgatttcttagatgagtacatatttctggctgtaggaagagttgggtctacttcagagaacatcacacaaaaagtggtttgggtggaggagatagacaaaaggtcatttctgcttgaccttctaaatgcaacaggcaaggattccctgactctagtgtttgtggagaccaaaaagggggcagattcgctggaggatttcttataccatgaaggatatgcttgtaccagtatccatggagaccgttctcagagagatagggaagaggcccttcaccagttccgctcaggaaaaagcccaattctagtggctacagcagtagcagcaagaggactggatatttcaaatgtgaagcatgttattaattttgacctgcctagtgatatcgaagaatatgtgcatcgcataggccgtacaggccgtgtgggaaaccttggtcttgccacctcattctttaatgaaaggaatataaatatcacaaaggatttactggatcttcttgttgaagcaaaacaagaagtgccttcttggttagagaacatggcttttgaacaccactacaagggtagcagtcgtggacgttctaagagcagtcgatttagtggagggtttggtgccagagactaccgacagagtagcggtgccagcagttccagcttcagcagcagccgtgcaagcagcagtcgaagtggtggaggtggccatggcggcagtcgaggatttggtggaggtggctacggaggcttttacaacagtgatggatatggagggaattataactcccagggggttgactggtggggtaacTCTAGA |
| Vector structure | 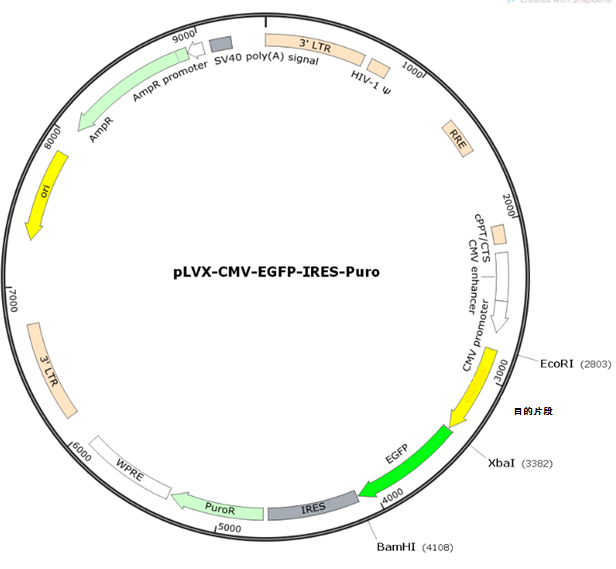 |

**Sequencing results of pc-DDX3X (pcDNA3.1-mDDX3X) plasmid**

*Tag sequence was Yellow highlighted and gene sequence was green highlighted.

Vector structure


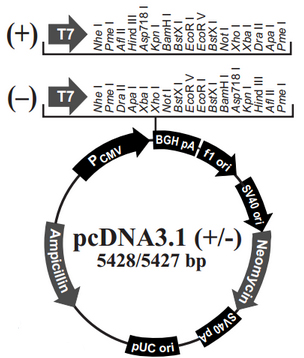


mDDX3X-Myc

CATGACCTTATGGGACTTTCCTACTTGGCAGTACATCTACGTATTAGTCATCGCTATTACCATGGTGATGCGGTTTTGGCAGTACATCAATGGGCGTGGATAGCGGTTTGACTCACGGGGATTTCCAAGTCTCCACCCCATTGACGTCAATGGGAGTTTGTTTTGGCACCAAAATCAACGGGACTTTCCAAAATGTCGTAACAACTCCGCCCCATTGACGCAAATGGGCGGTAGGCGTGTACGGTGGGAGGTCTATATAAGCAGAGCTCTCTGGCTAACTAGAGAACCCACTGCTTACTGGCTTATCGAAATTAATACGACTCACTATAGGGAGACCCAAGCTGGCTAGTTAAGCTTGGTACCGAGCTCGGATCCGCCACCATGAGTCATGTGGCAGTGGAAAATGCGCTCGGGCTGGACCAGCAGTTTGCTGGCCTAGACCTGAACTCTTCAGATAATCAGAGTGGAGGAAGTACAGCAAGCAAAGGGCGTTATATCCCACCTCATTTAAGGAACAGAGAAGCTACTAAAGGATTCTATGACAAAGACAGTTCAGGGTGGAGTTCTAGTAAAGATAAGGATGCATACAGCAGTTTTGGATCACGGGGTGATTCAAGAGGGAAGTCTAGCTTCTTTGGAGATCGTGGAAGTGGATCAAGGGGAAGGTTTGATGATCGTGGACGGGGAGACTATGATGGCATTGGTGGCCGTGGAGATAGAAGTGGCTTTGGCAAATTTGAAAGAGGTGGAAATAGTCGCTGGTGTGACAAATCAGATGAAGATGACTGGTCAAAGCCACTCCCACCAAGTGAACGATTGGAACAGGAACTCTTTTCTGGAGGCAATACTGGGATTAACTTTGAGAAATATGATGACATTCCAGTCGAAGCAACAGGCAACAACTGTCCTCCACACATTGAAAGTTTCAGTGATGTCGAGATGGGAGAAATTATTATGGGAAACATTGAGCTTACTCGTTATACTCGCCCAACTCCAGTGCAGAAGCATGCTATTCCTATTATCAAAGAGAAAAGAGACTTGATGGCTTGTGCTCAAACAGGCTCTGGAAAAACTGCAGCATTTCTCTTGCCCATCTTGAGTCAGATCTATGCTGATGGTCCAGGAGAAGCTCTGAGGGCTATGAAGGAAAATGGAAGATATGGCCGTCGTAAACAGTATCCAATCTCTTTGGTACTGGCACCAACGAGAGAATTGGCAGTGCAGATCTATGAGGAAGCCAGAAAATTCTCATACCGATCTAGAGTCCGTCCTTGCGTGGTTTATGGTGGTGCTGAAATTGGCCAGCAGATTCGAGACTTAGAACGTGGATGCCACTTGTTAGTAGCCACTCCAGGACGTCTAGTGGATATGATGGAGAGAGGGAAGATCGGGTTAGACTTCTGCAAATACCTGGTGTTAGATGAAGCTGACCGGATGTTAGATATGGGGTTTGAACCTCAGATACGAAGAATAGTTGAACAAGACACTATGCCTCCAAAAGGTGTCCGCCACACTATGATGTTTAGTGCTACTTTTCCTAAGGAAATACAGATGCTGGCCCGTGATTTCTTAGATGAGTACATATTTCTGGCTGTAGGAAGAGTTGGGTCTACTTCAGAGAACATCACACAAAAAGTGGTTTGGGTGGAGGAGATAGACAAAAGGTCATTTCTGCTTGACCTTCTAAATGCAACAGGCAAGGATTCCCTGACTCTAGTGTTTGTGGAGACCAAAAAGGGGGCAGATTCGCTGGAGGATTTCTTATACCATGAAGGATATGCTTGTACCAGTATCCATGGAGACCGTTCTCAGAGAGATAGGGAAGAGGCCCTTCACCAGTTCCGCTCAGGAAAAAGCCCAATTCTAGTGGCTACAGCAGTAGCAGCAAGAGGACTGGATATTTCAAATGTGAAGCATGTTATTAATTTTGACCTGCCTAGTGATATCGAAGAATATGTGCATCGCATAGGCCGTACAGGCCGTGTGGGAAACCTTGGTCTTGCCACCTCATTCTTTAATGAAAGGAATATAAATATCACAAAGGATTTACTGGATCTTCTTGTTGAAGCAAAACAAGAAGTGCCTTCTTGGTTAGAGAACATGGCTTTTGAACACCACTACAAGGGTAGCAGTCGTGGACGTTCTAAGAGCAGTCGATTTAGTGGAGGGTTTGGTGCCAGAGACTACCGACAGAGTAGCGGTGCCAGCAGTTCCAGCTTCAGCAGCAGCCGTGCAAGCAGCAGTCGAAGTGGTGGAGGTGGCCATGGCGGCAGTCGAGGATTTGGTGGAGGTGGCTACGGAGGCTTTTACAACAGTGATGGATATGGAGGGAATTATAACTCCCAGGGGGTTGACTGGTGGGGTAACCTCGAGTCTAGAGGGCCCTTCGAACAAAAACTCATCTCAGAAGAGGATCTGTGAGTTTAAACCCGCTGATCAGCCTCGACTGTGCCTTCTAGTTGCCAGCCATCTGTTGTTTGCCCCTCCCCCGTGCCTTCCTTGACCCTGGAAGGTGCCACTCCCACTGTCCTTTCCTAATAAAATGAGGAAATTGCATCGCATTGTCTGAGTAGGTGTCATTCTATTCTGGGGGGTGGGGTGGGGCAGGACAGCAAGGGGGAGGATTGGGAAGACAATAGCAGGCATGCTGGGGATGCGGTGGGCTCTATGGCTTCTGAGGCGGAAAGAACCAGCTGGGGCTCTAGGGGGTATCCCCACGCGCCCTGTAGCGGCGCATTAAGCGCGGCGGGTGTGGTGGTTACGCGCAGCGTGACCGCTACACTTGCCAGCGCCCTAGCGCCCGCTCCTTTCGCTTTCTTCCCTTCCTTTCTCGCCACGTTCGCCGGCTTTCCCCGTCAAGCTCTAAATCGGGGGCTCCCTTTAGGGTTCCGATTTAGTGCTTTACGGCACCTCGACCCCAAAAAACTTGATTAGGGTGATGGTTCACGTAGTGGGCCATCGCCCTGATAGACGGTTTTTCGCCCTTTGACGTTGGAGTCCACGTTCTTTAATAGTGGACTCTTGTTCCAAACTGGAACAACACTCAACCCTATCTCGGTCT

**Sequencing results of shRNA BGN plasmids**

*Interference sequence was green highlighted

Vector structure


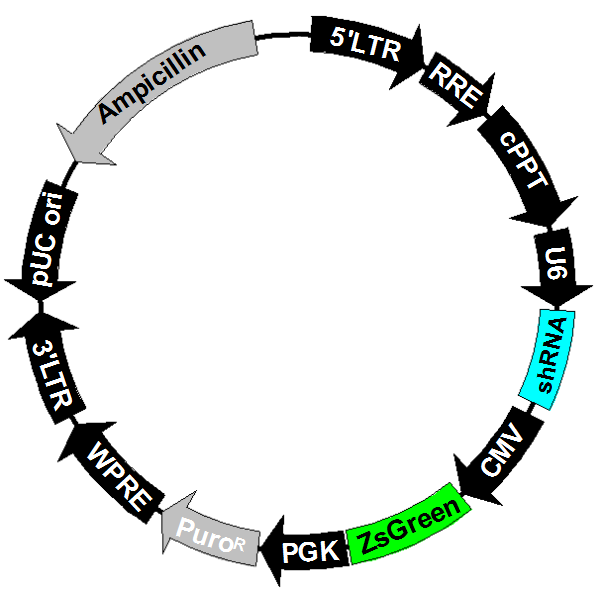


mBgn-sh1

ACAAATTACAAAAATTCAAAATTTTCGGGTTTATTACAGGGACAGCAGAGATCCAGTTTATCGATCTGGGCAGGAAGAGGGCCTATTTCCCATGATTCCTTCATATTTGCATATACGATACAAGGCTGTTAGAGAGATAATTAGAATTAATTTGACTGTAAACACAAAGATATTAGTACAAAATACGTGACGTAGAAAGTAATAATTTCTTGGGTAGTTTGCAGTTTTAAAATTATGTTTTAAAATGGACTATCATATGCTTACCGTAACTTGAAAGTATTTCGATTTCTTGGCTTTATATATCTTGTGGAAAGGACGAGGATCCGGACTTCACCTTGGATGATGGTTCAAGAGACCATCATCCAAGGTGAAGTCCTTTTTTGAATTCTAGTTATTAATAGTAATCAATTACGGGGTCATTAGTTCATAGCCCATATATGGAGTTCCGCGTTACATAACTTACGGTAAATGGCCCGCCTGGCTGACCGCCCAACGACCCCCGCCCATTGACGTCAATAATGACGTATGTTCCCATAGTAACGCCAATAGGGACTTTCCATTGACGTCAATGGGTGGAGTATTTACGGTAAACTGCCCACTTGGCAGTACATCAAGTGTATCATATGCCAAGTACGCCCCCTATTGACGTCAATGACGGTAAATGGCCCGCCTGGCATTATGCCCAGTA

mBgn-sh2

AATTACAAAAATTCAAAATTTTCGGGTTTATTACAGGGACAGCAGAGATCCAGTTTATCGATCTGGGCAGGAAGAGGGCCTATTTCCCATGATTCCTTCATATTTGCATATACGATACAAGGCTGTTAGAGAGATAATTAGAATTAATTTGACTGTAAACACAAAGATATTAGTACAAAATACGTGACGTAGAAAGTAATAATTTCTTGGGTAGTTTGCAGTTTTAAAATTATGTTTTAAAATGGACTATCATATGCTTACCGTAACTTGAAAGTATTTCGATTTCTTGGCTTTATATATCTTGTGGAAAGGACGAGGATCCGGAGAACAGTGGCTTTGAACCTTCAAGAGAGGTTCAAAGCCACTGTTCTCCTTTTTTGAATTCTAGTTATTAATAGTAATCAATTACGGGGTCATTAGTTCATAGCCCATATATGGAGTTCCGCGTTACATAACTTACGGTAAATGGCCCGCCTGGCTGACCGCCCAACGACCCCCGCCCATTGACGTCAATAATGACGTATGTTCCCATAGTAACGCCAATAGGGACTTTCCATTGACGTCAATGGGTGGAGTATTTACGGTAAACTGCCCACTTGGCAGTACATCAAGTGTATCATATGCCAAGTACGCCCCCTATTGACGTCAATGACGGTAAATGGCCCGCCTGGCATTATGCCCAGTACATGACCTTATGGGACT

mBgn-sh3

TACAGGGACAGCAGAGATCCAGTTTATCGATCTGGGCAGGAAGAGGGCCTATTTCCCATGATTCCTTCATATTTGCATATACGATACAAGGCTGTTAGAGAGATAATTAGAATTAATTTGACTGTAAACACAAAGATATTAGTACAAAATACGTGACGTAGAAAGTAATAATTTCTTGGGTAGTTTGCAGTTTTAAAATTATGTTTTAAAATGGACTATCATATGCTTACCGTAACTTGAAAGTATTTCGATTTCTTGGCTTTATATATCTTGTGGAAAGGACGAGGATCCGGTTGGGCTTAGGTCACAATCTTCAAGAGAGATTGTGACCTAAGCCCAACCTTTTTTGAATTCTAGTTATTAATAGTAATCAATTACGGGGTCATTAGTTCATAGCCCATATATGGAGTTCCGCGTTACATAACTTACGGTAAATGGCCCGCCTGGCTGACCGCCCAACGACCCCCGCCCATTGACGTCAATAATGACGTATGTTCCCATA

**Sequencing results of pcDNA3.1 BGN plasmids**

*Tag sequence was Yellow highlighted and gene sequence was green highlighted.

Vector structure


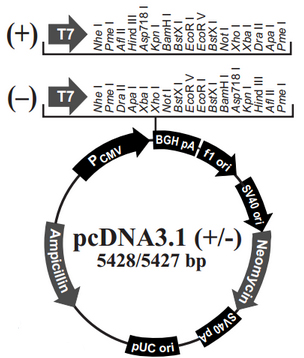


mBgn-HA

ACGGTAAATGGCCCGCCTGGCATTATGCCCAGTACATGACCTTATGGGACTTTCCTACTTGGCAGTACATCTACGTATTAGTCATCGCTATTACCATGGTGATGCGGTTTTGGCAGTACATCAATGGGCGTGGATAGCGGTTTGACTCACGGGGATTTCCAAGTCTCCACCCCATTGACGTCAATGGGAGTTTGTTTTGGCACCAAAATCAACGGGACTTTCCAAAATGTCGTAACAACTCCGCCCCATTGACGCAAATGGGCGGTAGGCGTGTACGGTGGGAGGTCTATATAAGCAGAGCTCTCTGGCTAACTAGAGAACCCACTGCTTACTGGCTTATCGAAATTAATACGACTCACTATAGGGAGACCCAAGCTGGCTAGCGTTTAAACTTAAGCTTGGTACCGAGCTCGGATCCGCCACCATGTGTCCCCTGTGGCTACTCACCTTGCTGCTGGCCCTGAGCCAGGCCTTGCCCTTTGAGCAGAAGGGTTTCTGGGACTTCACCTTGGATGATGGGCTGCTCATGATGAATGATGAGGAGGCTTCAGGTTCAGACACCACTTCAGGTGTCCCCGACCTGGACTCTGTCACACCTACCTTCAGTGCCATGTGTCCTTTCGGTTGCCACTGCCACCTGCGGGTTGTTCAGTGCTCTGACTTGGGTCTGAAGACTGTGCCCAAGGAGATCTCACCTGACACCACACTGCTAGACCTGCAGAACAATGACATTTCTGAGCTTCGCAAGGATGACTTCAAAGGCCTCCAGCACCTCTACGCCCTGGTCTTGGTAAACAATAAGATCTCCAAGATCCATGAGAAGGCCTTTAGCCCTCTGCGGAAGCTGCAAAAACTCTACATCTCCAAGAACCACCTGGTGGAGATTCCTCCCAACCTGCCCAGCTCCCTGGTAGAACTACGAATCCATGACAACCGTATCCGCAAAGTGCCCAAGGGCGTGTTCAGCGGGCTCCGGAACATGAACTGCATTGAGATGGGCGGGAATCCCCTGGAGAACAGTGGCTTTGAACCAGGAGCCTTTGATGGCCTGAAGCTCAATTACCTGCGCATCTCAGAGGCCAAGCTCACTGGCATCCCCAAAGATCTCCCTGAGACCCTGAACGAACTTCACCTGGACCACAACAAAATCCAGGCTATTGAGTTGGAGGACCTACTTCGATACTCCAAGCTGTACAGGTTGGGCTTAGGTCACAATCAGATTCGGATGATTGAGAATGGGAGCCTGAGTTTTCTGCCTACCCTGAGGGAACTTCACTTGGACAACAACAAGCTGTCCCGGGTGCCTGCTGGCCTCCCAGATCTCAAGCTCCTCCAGGTTGTCTATCTGCACTCCAACAACATCACCAAGGTGGGCATCAATGACTTCTGTCCTATGGGCTTCGGAGTCAAGAGGGCCTACTATAATGGCATCAGCCTCTTCAACAACCCTGTGCCCTACTGGGAAGTGCAGCCTGCCACCTTCCGCTGCGTTACTGACCGCCTGGCCATCCAATTTGGAAATTATAAGAAGTACCCATACGACGTCCCAGACTACGCTTAGGAATTCTGCAGATATCCAGCACAGTGGCGGCCGCTCGAGTCTAGAGGGCCCGTTTAAACCCGCTGATCAGCCTCGACTGTGCCTTCTAGTTGCCAGCCATCTGTTGTTTGCCCCTCCCCCGTGCCTTCCTTGACCCTGGAAGGTGCCACTCCCACTGTCCTTTCCTAATAAAATGAGGAAATTGCATCGCATTGTCTGAGTAGGTGTCATTCTATTCTGGGGGGTGGGGTGGGGCAGGACAGCAAGGGGGAGGATTGGGAAGACAATAGCAGGCATGCTGGGGATGCGGTGGGCTCTATGGCTTCTGAGGCGGAAAGAACCAGCTGGGGCTCTAGGGGGTATCCCCACGCGCCCTGTAGCGGCGCATTAAGCGCGGCGGGTGTGGTGGTTACGCGCAGCGTGACCGCTACACTTGCCAGCGCCCTAGCGCCCGCTCCTTTCGCTTTCTTCCCTTCCTTTCTCGCCACGTTCGCCGGCTTTCCCCGTCAAGCTCTAAATCGGGGGCTCCCTTTAGGGTTCCGATTTAGTGCTTTACGGCACCTCGACCCCAAAAAACTTGATTAGGGTGATGGTTCACGTAGTGGGC

mBgn-HA（Δ64-77aa）

GATAGCGGTTTGACTCACGGGGATTTCCAAGTCTCCACCCCATTGACGTCAATGGGAGTTTGTTTTGGCACCAAAATCAACGGGACTTTCCAAAATGTCGTAACAACTCCGCCCCATTGACGCAAATGGGCGGTAGGCGTGTACGGTGGGAGGTCTATATAAGCAGAGCTCTCTGGCTAACTAGAGAACCCACTGCTTACTGGCTTATCGAAATTAATACGACTCACTATAGGGAGACCCAAGCTGGCTAGCGTTTAAACTTAAGCTTGGTACCGAGCTCGGATCCGCCACCATGTGTCCCCTGTGGCTACTCACCTTGCTGCTGGCCCTGAGCCAGGCCTTGCCCTTTGAGCAGAAGGGTTTCTGGGACTTCACCTTGGATGATGGGCTGCTCATGATGAATGATGAGGAGGCTTCAGGTTCAGACACCACTTCAGGTGTCCCCGACCTGGACTCTGTCACACCTACCTTCAGTGCCATGTCTGACTTGGGTCTGAAGACTGTGCCCAAGGAGATCTCACCTGACACCACACTGCTAGACCTGCAGAACAATGACATTTCTGAGCTTCGCAAGGATGACTTCAAAGGCCTCCAGCACCTCTACGCCCTGGTCTTGGTAAACAATAAGATCTCCAAGATCCATGAGAAGGCCTTTAGCCCTCTGCGGAAGCTGCAAAAACTCTACATCTCCAAGAACCACCTGGTGGAGATTCCTCCCAACCTGCCCAGCTCCCTGGTAGAACTACGAATCCATGACAACCGTATCCGCAAAGTGCCCAAGGGCGTGTTCAGCGGGCTCCGGAACATGAACTGCATTGAGATGGGCGGGAATCCCCTGGAGAACAGTGGCTTTGAACCAGGAGCCTTTGATGGCCTGAAGCTCAATTACCTGCGCATCTCAGAGGCCAAGCTCACTGGCATCCCCAAAGATCTCCCTGAGACCCTGAACGAACTTCACCTGGACCACAACAAAATCCAGGCTATTGAGTTGGAGGACCTACTTCGATACTCCAAGCTGTACAGGTTGGGCTTAGGTCACAATCAGATTCGGATGATTGAGAATGGGAGCCTGAGTTTTCTGCCTACCCTGAGGGAACTTCACTTGGACAACAACAAGCTGTCCCGGGTGCCTGCTGGCCTCCCAGATCTCAAGCTCCTCCAGGTTGTCTATCTGCACTCCAACAACATCACCAAGGTGGGCATCAATGACTTCTGTCCTATGGGCTTCGGAGTCAAGAGGGCCTACTATAATGGCATCAGCCTCTTCAACAACCCTGTGCCCTACTGGGAAGTGCAGCCTGCCACCTTCCGCTGCGTTACTGACCGCCTGGCCATCCAATTTGGAAATTATAAGAAGTACCCATACGACGTCCCAGACTACGCTTAGGAATTCTGCAGATATCCAGCACAGTGGCGGCCGCTCGAGTCTAGAGGGCCCGTTTAAACCCGCTGATCAGCCTCGACTGTGCCTTCTAGTTGCCAGCCATCTGTTGTTTGCCCCTCCCCCGTGCCTTCCTTGACCCTGGAAGGTGCCACTCCCACTGTCCTTTCCTAATAAAATGAGGAAATTGCATCGCATTGTCTGAGTAGGTGTCATTCTATTCTGGGGGGTGGGGTGGGGCAGGACAGCAAGGGGGAGGATTGGGAAGACAATAGCAGGCATGCTGGGGATGCGGTGGGCTCTATGGCTTCTGAGGCGGAAAGAACCA

mBgn-HA（Δ83-221aa）

GGGACTTTCCTACTTGGCAGTACATCTACGTATTAGTCATCGCTATTACCATGGTGATGCGGTTTTGGCAGTACATCAATGGGCGTGGATAGCGGTTTGACTCACGGGGATTTCCAAGTCTCCACCCCATTGACGTCAATGGGAGTTTGTTTTGGCACCAAAATCAACGGGACTTTCCAAAATGTCGTAACAACTCCGCCCCATTGACGCAAATGGGCGGTAGGCGTGTACGGTGGGAGGTCTATATAAGCAGAGCTCTCTGGCTAACTAGAGAACCCACTGCTTACTGGCTTATCGAAATTAATACGACTCACTATAGGGAGACCCAAGCTGGCTAGCGTTTAAACTTAAGCTTGGTACCGAGCTCGGATCCGCCACCATGTGTCCCCTGTGGCTACTCACCTTGCTGCTGGCCCTGAGCCAGGCCTTGCCCTTTGAGCAGAAGGGTTTCTGGGACTTCACCTTGGATGATGGGCTGCTCATGATGAATGATGAGGAGGCTTCAGGTTCAGACACCACTTCAGGTGTCCCCGACCTGGACTCTGTCACACCTACCTTCAGTGCCATGTGTCCTTTCGGTTGCCACTGCCACCTGCGGGTTGTTCAGTGCTCTGACTTGGGTCTGACTGGCATCCCCAAAGATCTCCCTGAGACCCTGAACGAACTTCACCTGGACCACAACAAAATCCAGGCTATTGAGTTGGAGGACCTACTTCGATACTCCAAGCTGTACAGGTTGGGCTTAGGTCACAATCAGATTCGGATGATTGAGAATGGGAGCCTGAGTTTTCTGCCTACCCTGAGGGAACTTCACTTGGACAACAACAAGCTGTCCCGGGTGCCTGCTGGCCTCCCAGATCTCAAGCTCCTCCAGGTTGTCTATCTGCACTCCAACAACATCACCAAGGTGGGCATCAATGACTTCTGTCCTATGGGCTTCGGAGTCAAGAGGGCCTACTATAATGGCATCAGCCTCTTCAACAACCCTGTGCCCTACTGGGAAGTGCAGCCTGCCACCTTCCGCTGCGTTACTGACCGCCTGGCCATCCAATTTGGAAATTATAAGAAGTACCCATACGACGTCCCAGACTACGCTTAGGAATTCTGCAGATATCCAGCACAGTGGCGGCCGCTCGAGTCTAGAGGGCCCGTTTAAACCCGCTGATCAGCCTCGACTGTGCCTTCTAGTTGCCAGCCATCTGTTGTTTGCCCCTCCCCCGTGCCTTCCTTGACCCTGGAAGGTGCCACTCCCACTGTCCTTTCCTAATAAAATGAGGAAATTGCATCGCATTGTCTGAGTAGGTGTCATTCTATTCTGGGGGGTGGGGTGGGGCAGGACAGCAAGGGGGAGGATTGGGAAGACAATAGCAGGCATGCTGGGGATGCGGTGGGCTCTATGGCTTCTGAGGCGGAAAGAACCAGCTGGGGCTCTAGGGGGTATCC

mBgn-HA（Δ222-369aa）

AAATGTCGTAACAACTCCGCCCCATTGACGCAAATGGGCGGTAGGCGTGTACGGTGGGAGGTCTATATAAGCAGAGCTCTCTGGCTAACTAGAGAACCCACTGCTTACTGGCTTATCGAAATTAATACGACTCACTATAGGGAGACCCAAGCTGGCTAGCGTTTAAACTTAAGCTTGGTACCGAGCTCGGATCCGCCACCATGTGTCCCCTGTGGCTACTCACCTTGCTGCTGGCCCTGAGCCAGGCCTTGCCCTTTGAGCAGAAGGGTTTCTGGGACTTCACCTTGGATGATGGGCTGCTCATGATGAATGATGAGGAGGCTTCAGGTTCAGACACCACTTCAGGTGTCCCCGACCTGGACTCTGTCACACCTACCTTCAGTGCCATGTGTCCTTTCGGTTGCCACTGCCACCTGCGGGTTGTTCAGTGCTCTGACTTGGGTCTGAAGACTGTGCCCAAGGAGATCTCACCTGACACCACACTGCTAGACCTGCAGAACAATGACATTTCTGAGCTTCGCAAGGATGACTTCAAAGGCCTCCAGCACCTCTACGCCCTGGTCTTGGTAAACAATAAGATCTCCAAGATCCATGAGAAGGCCTTTAGCCCTCTGCGGAAGCTGCAAAAACTCTACATCTCCAAGAACCACCTGGTGGAGATTCCTCCCAACCTGCCCAGCTCCCTGGTAGAACTACGAATCCATGACAACCGTATCCGCAAAGTGCCCAAGGGCGTGTTCAGCGGGCTCCGGAACATGAACTGCATTGAGATGGGCGGGAATCCCCTGGAGAACAGTGGCTTTGAACCAGGAGCCTTTGATGGCCTGAAGCTCAATTACCTGCGCATCTCAGAGGCCAAGCTCTACCCATACGACGTCCCAGACTACGCTTAGGAATTCTGCAGATATCCAGCACAGTGGCGGCCGCTCGAGTCTAGAGGGCCCGTTTAAACCCG

**Sequencing results of pcDNA3.1 TLR4 plasmids**

*Tag sequence was Yellow highlighted and gene sequence was green highlighted.

Vector structure


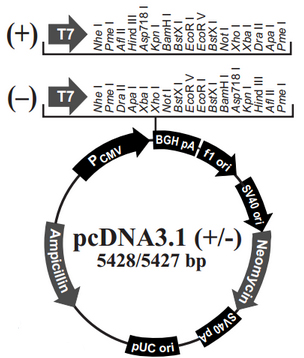


mTLr4-Flag

TGACGGTAAATGGCCCGCCTGGCATTATGCCCAGTACATGACCTTATGGGACTTTCCTACTTGGCAGTACATCTACGTATTAGTCATCGCTATTACCATGGTGATGCGGTTTTGGCAGTACATCAATGGGCGTGGATAGCGGTTTGACTCACGGGGATTTCCAAGTCTCCACCCCATTGACGTCAATGGGAGTTTGTTTTGGCACCAAAATCAACGGGACTTTCCAAAATGTCGTAACAACTCCGCCCCATTGACGCAAATGGGCGGTAGGCGTGTACGGTGGGAGGTCTATATAAGCAGAGCTCTCTGGCTAACTAGAGAACCCACTGCTTACTGGCTTATCGAAATTAATACGACTCACTATAGGGAGACCCAAGCTGGCTAGCGTTTAAACTTAAGCTTGGTACCGAGCTCGGATCCGCCACCATGATGCCTCCCTGGCTCCTGGCTAGGACTCTGATCATGGCACTGTTCTTCTCCTGCCTGACACCAGGAAGCTTGAATCCCTGCATAGAGGTAGTTCCTAATATTACCTACCAATGCATGGATCAGAAACTCAGCAAAGTCCCTGATGACATTCCTTCTTCAACCAAGAACATAGATCTGAGCTTCAACCCCTTGAAGATCTTAAAAAGCTATAGCTTCTCCAATTTTTCAGAACTTCAGTGGCTGGATTTATCCAGGTGTGAAATTGAAACAATTGAAGACAAGGCATGGCATGGCTTACACCACCTCTCAAACTTGATACTGACAGGAAACCCTATCCAGAGTTTTTCCCCAGGAAGTTTCTCTGGACTAACAAGTTTAGAGAATCTGGTGGCTGTGGAGACAAAATTGGCCTCTCTAGAAAGCTTCCCTATTGGACAGCTTATAACCTTAAAGAAACTCAATGTGGCTCACAATTTTATACATTCCTGTAAGTTACCTGCATATTTTTCCAATCTGACGAACCTAGTACATGTGGATCTTTCTTATAACTATATTCAAACTATTACTGTCAACGACTTACAGTTTCTACGTGAAAATCCACAAGTCAATCTCTCTTTAGACATGTCTTTGAACCCAATTGACTTCATTCAAGACCAAGCCTTTCAGGGAATTAAGCTCCATGAACTGACTCTAAGAGGTAATTTTAATAGCTCAAATATAATGAAAACTTGCCTTCAAAACCTGGCTGGTTTACACGTCCATCGGTTGATCTTGGGAGAATTTAAAGATGAAAGGAATCTGGAAATTTTTGAACCCTCTATCATGGAAGGACTATGTGATGTGACCATTGATGAGTTCAGGTTAACATATACAAATGATTTTTCAGATGATATTGTTAAGTTCCATTGCTTGGCGAATGTTTCTGCAATGTCTCTGGCAGGTGTATCTATAAAATATCTAGAAGATGTTCCTAAACATTTCAAATGGCAATCCTTATCAATCATTAGATGTCAACTTAAGCAGTTTCCAACTCTGGATCTACCCTTTCTTAAAAGTTTGACTTTAACTATGAACAAAGGGTCTATCAGTTTTAAAAAAGTGGCCCTACCAAGTCTCAGCTATCTAGATCTTAGTAGAAATGCACTGAGCTTTAGTGGTTGCTGTTCTTATTCTGATTTGGGAACAAACAGCCTGAGACACTTAGACCTCAGCTTCAATGGTGCCATCATTATGAGTGCCAATTTCATGGGTCTAGAAGAGCTGCAGCACCTGGATTTTCAGCACTCTACTTTAAAAAGGGTCACAGAATTCTCAGCGTTCTTATCCCTTGAAAAGCTACTTTACCTTGACATCTCTTATACTAACACCAAAATTGACTTCGATGGTATATTTCTTGGCTTGACCAGTCTCAACACATTAAAAATGGCTGGCAATTCTTTCAAAGACAACACCCTTTCAAATGTCTTTGCAAACACAACAAACTTGACATTCCTGGATCTTTCTAAATGTCAATTGGAACAAATATCTTGGGGGGTATTTGACACCCTCCATAGACTTCAATTATTAAATATGAGTCACAACAATCTATTGTTTTTGGATTCATCCCATTATAACCAGCTGTATTCCCTCAGCACTCTTGATTGCAGTTTCAATCGCATAGAGACATCTAAAGGAATACTGCAACATTTTCCAAAGAGTCTAGCCTTCTTCAATCTTACTAACAATTCTGTTGCTTGTATATGTGAACATCAGAAATTCCTGCAGTGGGTCAAGGAACAGAAGCAGTTCTTGGTGAATGTTGAACAAATGACATGTGCAACACCTGTAGAGATGAATACCTCCTTAGTGTTGGATTTTAATAATTCTACCTGTTATATGTACAAGACAATCATCAGTGTGTCAGTGGTCAGTGTGATTGTGGTATCCACTGTAGCATTTCTGATATACCACTTCTATTTTCACCTGATACTTATTGCTGGCTGTAAAAAGTACAGCAGAGGAGAAAGCATCTATGATGCATTTGTGATCTACTCGAGTCAGAATGAGGACTGGGTGAGAAATGAGCTGGTAAAGAATTTAGAAGAAGGAGTGCCCCGCTTTCACCTCTGCCTTCACTACAGAGACTTTATTCCTGGTGTAGCCATTGCTGCCAACATCATCCAGGAAGGCTTCCACAAGAGCCGGAAGGTTATTGTGGTAGTGTCTAGACACTTTATTCAGAGCCGTTGGTGTATCTTTGAATATGAGATTGCTCAAACATGGCAGTTTCTGAGCAGCCGCTCTGGCATCATCTTCATTGTCCTTGAGAAGGTTGAGAAGTCCCTGCTGAGGCAGCAGGTGGAATTGTATCGCCTTCTTAGCAGAAACACCTACCTGGAATGGGAGGACAATCCTCTGGGGAGGCACATCTTCTGGAGAAGACTTAAAAATGCCCTATTGGATGGAAAAGCCTCGAATCCTGAGCAAACAGCAGAGGAAGAACAAGAAACGGCAACTTGGACCGATTACAAGGATGACGACGATAAGTGAGAATTCTGCAGATATCCAGCACAGTGGCGGCCGCTCGAGTCTAGAGGGCCCGTTTAAACCCGCTGATCAGCCTCGACTGTGCCTTCTAGTTGCCAGCCATCTGTTGTTTGCCCCTCCCCCGTGCCTTCCTTGACCCTGGAAGGTGCCACTCCCACTGTCCTTTCCTAATAAAATGAGGAAATTGCATCGCATTGTCTGAGTAGGTGTCATTCTATTCTGGGGGGTGGGGTGGGGCAGGACAGCAAGGGGGAGGATTGGGAAGACAATAGCAGGCATGCTGGGGATGCGGTGGGCTCTATGGCTTCTGAGGCGGAAAGAACCAGCTGGGGCTCTAGGGGGTAT

mTLr4-Flag（Δ1-53aa）CCCAGTACATGACCTTATGGGACTTTCCTACTTGGCAGTACATCTACGTATTAGTCATCGCTATTACCATGGTGATGCGGTTTTGGCAGTACATCAATGGGCGTGGATAGCGGTTTGACTCACGGGGATTTCCAAGTCTCCACCCCATTGACGTCAATGGGAGTTTGTTTTGGCACCAAAATCAACGGGACTTTCCAAAATGTCGTAACAACTCCGCCCCATTGACGCAAATGGGCGGTAGGCGTGTACGGTGGGAGGTCTATATAAGCAGAGCTCTCTGGCTAACTAGAGAACCCACTGCTTACTGGCTTATCGAAATTAATACGACTCACTATAGGGAGACCCAAGCTGGCTAGCGTTTAAACTTAAGCTTGGTACCGAGCTCGGATCCGCCACCATGTCAACCAAGAACATAGATCTGAGCTTCAACCCCTTGAAGATCTTAAAAAGCTATAGCTTCTCCAATTTTTCAGAACTTCAGTGGCTGGATTTATCCAGGTGTGAAATTGAAACAATTGAAGACAAGGCATGGCATGGCTTACACCACCTCTCAAACTTGATACTGACAGGAAACCCTATCCAGAGTTTTTCCCCAGGAAGTTTCTCTGGACTAACAAGTTTAGAGAATCTGGTGGCTGTGGAGACAAAATTGGCCTCTCTAGAAAGCTTCCCTATTGGACAGCTTATAACCTTAAAGAAACTCAATGTGGCTCACAATTTTATACATTCCTGTAAGTTACCTGCATATTTTTCCAATCTGACGAACCTAGTACATGTGGATCTTTCTTATAACTATATTCAAACTATTACTGTCAACGACTTACAGTTTCTACGTGAAAATCCACAAGTCAATCTCTCTTTAGACATGTCTTTGAACCCAATTGACTTCATTCAAGACCAAGCCTTTCAGGGAATTAAGCTCCATGAACTGACTCTAAGAGGTAATTTTAATAGCTCAAATATAATGAAAACTTGCCTTCAAAACCTGGCTGGTTTACACGTCCATCGGTTGATCTTGGGAGAATTTAAAGATGAAAGGAATCTGGAAATTTTTGAACCCTCTATCATGGAAGGACTATGTGATGTGACCATTGATGAGTTCAGGTTAACATATACAAATGATTTTTCAGATGATATTGTTAAGTTCCATTGCTTGGCGAATGTTTCTGCAATGTCTCTGGCAGGTGTATCTATAAAATATCTAGAAGATGTTCCTAAACATTTCAAATGGCAATCCTTATCAATCATTAGATGTCAACTTAAGCAGTTTCCAACTCTGGATCTACCCTTTCTTAAAAGTTTGACTTTAACTATGAACAAAGGGTCTATCAGTTTTAAAAAAGTGGCCCTACCAAGTCTCAGCTATCTAGATCTTAGTAGAAATGCACTGAGCTTTAGTGGTTGCTGTTCTTATTCTGATTTGGGAACAAACAGCCTGAGACACTTAGACCTCAGCTTCAATGGTGCCATCATTATGAGTGCCAATTTCATGGGTCTAGAAGAGCTGCAGCACCTGGATTTTCAGCACTCTACTTTAAAAAGGGTCACAGAATTCTCAGCGTTCTTATCCCTTGAAAAGCTACTTTACCTTGACATCTCTTATACTAACACCAAAATTGACTTCGATGGTATATTTCTTGGCTTGACCAGTCTCAACACATTAAAAATGGCTGGCAATTCTTTCAAAGACAACACCCTTTCAAATGTCTTTGCAAACACAACAAACTTGACATTCCTGGATCTTTCTAAATGTCAATTGGAACAAATATCTTGGGGGGTATTTGACACCCTCCATAGACTTCAATTATTAAATATGAGTCACAACAATCTATTGTTTTTGGATTCATCCCATTATAACCAGCTGTATTCCCTCAGCACTCTTGATTGCAGTTTCAATCGCATAGAGACATCTAAAGGAATACTGCAACATTTTCCAAAGAGTCTAGCCTTCTTCAATCTTACTAACAATTCTGTTGCTTGTATATGTGAACATCAGAAATTCCTGCAGTGGGTCAAGGAACAGAAGCAGTTCTTGGTGAATGTTGAACAAATGACATGTGCAACACCTGTAGAGATGAATACCTCCTTAGTGTTGGATTTTAATAATTCTACCTGTTATATGTACAAGACAATCATCAGTGTGTCAGTGGTCAGTGTGATTGTGGTATCCACTGTAGCATTTCTGATATACCACTTCTATTTTCACCTGATACTTATTGCTGGCTGTAAAAAGTACAGCAGAGGAGAAAGCATCTATGATGCATTTGTGATCTACTCGAGTCAGAATGAGGACTGGGTGAGAAATGAGCTGGTAAAGAATTTAGAAGAAGGAGTGCCCCGCTTTCACCTCTGCCTTCACTACAGAGACTTTATTCCTGGTGTAGCCATTGCTGCCAACATCATCCAGGAAGGCTTCCACAAGAGCCGGAAGGTTATTGTGGTAGTGTCTAGACACTTTATTCAGAGCCGTTGGTGTATCTTTGAATATGAGATTGCTCAAACATGGCAGTTTCTGAGCAGCCGCTCTGGCATCATCTTCATTGTCCTTGAGAAGGTTGAGAAGTCCCTGCTGAGGCAGCAGGTGGAATTGTATCGCCTTCTTAGCAGAAACACCTACCTGGAATGGGAGGACAATCCTCTGGGGAGGCACATCTTCTGGAGAAGACTTAAAAATGCCCTATTGGATGGAAAAGCCTCGAATCCTGAGCAAACAGCAGAGGAAGAACAAGAAACGGCAACTTGGACCGATTACAAGGATGACGACGATAAGTGAGAATTCTGCAGATATCCAGCACAGTGGCGGCCGCTCGAGTCTAGAGGGCCCGTTTAAACCCGCTGATCAGCCTCGACTGTGCCTTCTAGTTGCCAGCCATCTGTTGTTTGCCCCTCCCCCGTGCCTTCCTTGACCCTGGAAGGTGCCACTCCCACTGTCCTTTCCTAATAAAATGAGGAAATTGCATCGCATTGTCTGAGTAGGTGTCATTCTATTCTGGGGGGTGGGGTGGGGCAGGACAGCAAGGGGGAGGATTGGGAAGACAATAGCAGGCATGCTGGGGATGCGGTGGGCTCTATGGCTTCTGAGGCGGAAAGAACCA

mTLr4-Flag（Δ54-198aa）TAGTAACGCCAATAGGGACTTTCCATTGACGTCAATGGGTGGAGTATTTACGGTAAACTGCCCACTTGGCAGTACATCAAGTGTATCATATGCCAAGTACGCCCCCTATTGACGTCAATGACGGTAAATGGCCCGCCTGGCATTATGCCCAGTACATGACCTTATGGGACTTTCCTACTTGGCAGTACATCTACGTATTAGTCATCGCTATTACCATGGTGATGCGGTTTTGGCAGTACATCAATGGGCGTGGATAGCGGTTTGACTCACGGGGATTTCCAAGTCTCCACCCCATTGACGTCAATGGGAGTTTGTTTTGGCACCAAAATCAACGGGACTTTCCAAAATGTCGTAACAACTCCGCCCCATTGACGCAAATGGGCGGTAGGCGTGTACGGTGGGAGGTCTATATAAGCAGAGCTCTCTGGCTAACTAGAGAACCCACTGCTTACTGGCTTATCGAAATTAATACGACTCACTATAGGGAGACCCAAGCTGGCTAGCGTTTAAACTTAAGCTTGGTACCGAGCTCGGATCCGCCACCATGATGCCTCCCTGGCTCCTGGCTAGGACTCTGATCATGGCACTGTTCTTCTCCTGCCTGACACCAGGAAGCTTGAATCCCTGCATAGAGGTAGTTCCTAATATTACCTACCAATGCATGGATCAGAAACTCAGCAAAGTCCCTGATGACATTCCTTCTTCAGAAAATCCACAAGTCAATCTCTCTTTAGACATGTCTTTGAACCCAATTGACTTCATTCAAGACCAAGCCTTTCAGGGAATTAAGCTCCATGAACTGACTCTAAGAGGTAATTTTAATAGCTCAAATATAATGAAAACTTGCCTTCAAAACCTGGCTGGTTTACACGTCCATCGGTTGATCTTGGGAGAATTTAAAGATGAAAGGAATCTGGAAATTTTTGAACCCTCTATCATGGAAGGACTATGTGATGTGACCATTGATGAGTTCAGGTTAACATATACAAATGATTTTTCAGATGATATTGTTAAGTTCCATTGCTTGGCGAATGTTTCTGCAATGTCTCTGGCAGGTGTATCTATAAAATATCTAGAAGATGTTCCTAAACATTTCAAATGGCAATCCTTATCAATCATTAGATGTCAACTTAAGCAGTTTCCAACTCTGGATCTACCCTTTCTTAAAAGTTTGACTTTAACTATGAACAAAGGGTCTATCAGTTTTAAAAAAGTGGCCCTACCAAGTCTCAGCTATCTAGATCTTAGTAGAAATGCACTGAGCTTTAGTGGTTGCTGTTCTTATTCTGATTTGGGAACAAACAGCCTGAGACACTTAGACCTCAGCTTCAATGGTGCCATCATTATGAGTGCCAATTTCATGGGTCTAGAAGAGCTGCAGCACCTGGATTTTCAGCACTCTACTTTAAAAAGGGTCACAGAATTCTCAGCGTTCTTATCCCTTGAAAAGCTACTTTACCTTGACATCTCTTATACTAACACCAAAATTGACTTCGATGGTATATTTCTTGGCTTGACCAGTCTCAACACATTAAAAATGGCTGGCAATTCTTTCAAAGACAACACCCTTTCAAATGTCTTTGCAAACACAACAAACTTGACATTCCTGGATCTTTCTAAATGTCAATTGGAACAAATATCTTGGGGGGTATTTGACACCCTCCATAGACTTCAATTATTAAATATGAGTCACAACAATCTATTGTTTTTGGATTCATCCCATTATAACCAGCTGTATTCCCTCAGCACTCTTGATTGCAGTTTCAATCGCATAGAGACATCTAAAGGAATACTGCAACATTTTCCAAAGAGTCTAGCCTTCTTCAATCTTACTAACAATTCTGTTGCTTGTATATGTGAACATCAGAAATTCCTGCAGTGGGTCAAGGAACAGAAGCAGTTCTTGGTGAATGTTGAACAAATGACATGTGCAACACCTGTAGAGATGAATACCTCCTTAGTGTTGGATTTTAATAATTCTACCTGTTATATGTACAAGACAATCATCAGTGTGTCAGTGGTCAGTGTGATTGTGGTATCCACTGTAGCATTTCTGATATACCACTTCTATTTTCACCTGATACTTATTGCTGGCTGTAAAAAGTACAGCAGAGGAGAAAGCATCTATGATGCATTTGTGATCTACTCGAGTCAGAATGAGGACTGGGTGAGAAATGAGCTGGTAAAGAATTTAGAAGAAGGAGTGCCCCGCTTTCACCTCTGCCTTCACTACAGAGACTTTATTCCTGGTGTAGCCATTGCTGCCAACATCATCCAGGAAGGCTTCCACAAGAGCCGGAAGGTTATTGTGGTAGTGTCTAGACACTTTATTCAGAGCCGTTGGTGTATCTTTGAATATGAGATTGCTCAAACATGGCAGTTTCTGAGCAGCCGCTCTGGCATCATCTTCATTGTCCTTGAGAAGGTTGAGAAGTCCCTGCTGAGGCAGCAGGTGGAATTGTATCGCCTTCTTAGCAGAAACACCTACCTGGAATGGGAGGACAATCCTCTGGGGAGGCACATCTTCTGGAGAAGACTTAAAAATGCCCTATTGGATGGAAAAGCCTCGAATCCTGAGCAAACAGCAGAGGAAGAACAAGAAACGGCAACTTGGACCGATTACAAGGATGACGACGATAAGTGAGAATTCTGCAGATATCCAGCACAGTGGCGGCCGCTCGAGTCTAGAGGGCCCGTTTAAACCCGCTGATCAGCCTCGACTGTGCCTTCTAGTTGCCAGCCATCTGTTGTTTGCCCCTCCCCCGTGCCTTCCTTGACCCTGGAAGGTGCCACTCCCACTGTCCTTTCCTAATAAAATGAGGAAATTGCATCGCATTGTCTGAGTAGGTGTCATTCTATTCTGGGGGGTGGGGTGGGGCAGGACAGCAAGGGGGAGGATTGGGAAGACAATAGCAGGCATGCTGGGGATGC

mTLr4-Flag（Δ 204-392aa）GGCGTGGATAGCGGTTTGACTCACGGGGATTTCCAAGTCTCCACCCCATTGACGTCAATGGGAGTTTGTTTTGGCACCAAAATCAACGGGACTTTCCAAAATGTCGTAACAACTCCGCCCCATTGACGCAAATGGGCGGTAGGCGTGTACGGTGGGAGGTCTATATAAGCAGAGCTCTCTGGCTAACTAGAGAACCCACTGCTTACTGGCTTATCGAAATTAATACGACTCACTATAGGGAGACCCAAGCTGGCTAGCGTTTAAACTTAAGCTTGGTACCGAGCTCGGATCCGCCACCATGATGCCTCCCTGGCTCCTGGCTAGGACTCTGATCATGGCACTGTTCTTCTCCTGCCTGACACCAGGAAGCTTGAATCCCTGCATAGAGGTAGTTCCTAATATTACCTACCAATGCATGGATCAGAAACTCAGCAAAGTCCCTGATGACATTCCTTCTTCAACCAAGAACATAGATCTGAGCTTCAACCCCTTGAAGATCTTAAAAAGCTATAGCTTCTCCAATTTTTCAGAACTTCAGTGGCTGGATTTATCCAGGTGTGAAATTGAAACAATTGAAGACAAGGCATGGCATGGCTTACACCACCTCTCAAACTTGATACTGACAGGAAACCCTATCCAGAGTTTTTCCCCAGGAAGTTTCTCTGGACTAACAAGTTTAGAGAATCTGGTGGCTGTGGAGACAAAATTGGCCTCTCTAGAAAGCTTCCCTATTGGACAGCTTATAACCTTAAAGAAACTCAATGTGGCTCACAATTTTATACATTCCTGTAAGTTACCTGCATATTTTTCCAATCTGACGAACCTAGTACATGTGGATCTTTCTTATAACTATATTCAAACTATTACTGTCAACGACTTACAGTTTCTACGTGAAAATCCACAAGTCGATTTGGGAACAAACAGCCTGAGACACTTAGACCTCAGCTTCAATGGTGCCATCATTATGAGTGCCAATTTCATGGGTCTAGAAGAGCTGCAGCACCTGGATTTTCAGCACTCTACTTTAAAAAGGGTCACAGAATTCTCAGCGTTCTTATCCCTTGAAAAGCTACTTTACCTTGACATCTCTTATACTAACACCAAAATTGACTTCGATGGTATATTTCTTGGCTTGACCAGTCTCAACACATTAAAAATGGCTGGCAATTCTTTCAAAGACAACACCCTTTCAAATGTCTTTGCAAACACAACAAACTTGACATTCCTGGATCTTTCTAAATGTCAATTGGAACAAATATCTTGGGGGGTATTTGACACCCTCCATAGACTTCAATTATTAAATATGAGTCACAACAATCTATTGTTTTTGGATTCATCCCATTATAACCAGCTGTATTCCCTCAGCACTCTTGATTGCAGTTTCAATCGCATAGAGACATCTAAAGGAATACTGCAACATTTTCCAAAGAGTCTAGCCTTCTTCAATCTTACTAACAATTCTGTTGCTTGTATATGTGAACATCAGAAATTCCTGCAGTGGGTCAAGGAACAGAAGCAGTTCTTGGTGAATGTTGAACAAATGACATGTGCAACACCTGTAGAGATGAATACCTCCTTAGTGTTGGATTTTAATAATTCTACCTGTTATATGTACAAGACAATCATCAGTGTGTCAGTGGTCAGTGTGATTGTGGTATCCACTGTAGCATTTCTGATATACCACTTCTATTTTCACCTGATACTTATTGCTGGCTGTAAAAAGTACAGCAGAGGAGAAAGCATCTATGATGCATTTGTGATCTACTCGAGTCAGAATGAGGACTGGGTGAGAAATGAGCTGGTAAAGAATTTAGAAGAAGGAGTGCCCCGCTTTCACCTCTGCCTTCACTACAGAGACTTTATTCCTGGTGTAGCCATTGCTGCCAACATCATCCAGGAAGGCTTCCACAAGAGCCGGAAGGTTATTGTGGTAGTGTCTAGACACTTTATTCAGAGCCGTTGGTGTATCTTTGAATATGAGATTGCTCAAACATGGCAGTTTCTGAGCAGCCGCTCTGGCATCATCTTCATTGTCCTTGAGAAGGTTGAGAAGTCCCTGCTGAGGCAGCAGGTGGAATTGTATCGCCTTCTTAGCAGAAACACCTACCTGGAATGGGAGGACAATCCTCTGGGGAGGCACATCTTCTGGAGAAGACTTAAAAATGCCCTATTGGATGGAAAAGCCTCGAATCCTGAGCAAACAGCAGAGGAAGAACAAGAAACGGCAACTTGGACCGATTACAAGGATGACGACGATAAGTGAGAATTCTGCAGATATCCAGCACAGTGGCGGCCGCTCGAGTCTAGAGGGCCCGTTTAAACCCGCTGATCAGCCTCGACTGTGCCTTCTAGTTGCCAGCCATCTGTTGTTTGCCCCTCCCCCGTGCCTTCCTTGACCCTGGAAGGTGCCACTCCCACTGTCCTTTCCTAATAAAATGAGGAAATTGCATCGCATTGTCTGAGTAGGTGTCATTCTATTCTGGGGGGTGGGGTGGGGCAGGACAGCAAGGGGGAGGATTGGGAAGACAATAGCAGGCATGCTGGGGATGCGGTGGGCTCTATGGCTTCTGAGGCGGAAAGAACCA

mTLr4-Flag（Δ398-564aa）CGGGACTTTCCAAAATGTCGTAACAACTCCGCCCCATTGACGCAAATGGGCGGTAGGCGTGTACGGTGGGAGGTCTATATAAGCAGAGCTCTCTGGCTAACTAGAGAACCCACTGCTTACTGGCTTATCGAAATTAATACGACTCACTATAGGGAGACCCAAGCTGGCTAGCGTTTAAACTTAAGCTTGGTACCGAGCTCGGATCCGCCACCATGATGCCTCCCTGGCTCCTGGCTAGGACTCTGATCATGGCACTGTTCTTCTCCTGCCTGACACCAGGAAGCTTGAATCCCTGCATAGAGGTAGTTCCTAATATTACCTACCAATGCATGGATCAGAAACTCAGCAAAGTCCCTGATGACATTCCTTCTTCAACCAAGAACATAGATCTGAGCTTCAACCCCTTGAAGATCTTAAAAAGCTATAGCTTCTCCAATTTTTCAGAACTTCAGTGGCTGGATTTATCCAGGTGTGAAATTGAAACAATTGAAGACAAGGCATGGCATGGCTTACACCACCTCTCAAACTTGATACTGACAGGAAACCCTATCCAGAGTTTTTCCCCAGGAAGTTTCTCTGGACTAACAAGTTTAGAGAATCTGGTGGCTGTGGAGACAAAATTGGCCTCTCTAGAAAGCTTCCCTATTGGACAGCTTATAACCTTAAAGAAACTCAATGTGGCTCACAATTTTATACATTCCTGTAAGTTACCTGCATATTTTTCCAATCTGACGAACCTAGTACATGTGGATCTTTCTTATAACTATATTCAAACTATTACTGTCAACGACTTACAGTTTCTACGTGAAAATCCACAAGTCAATCTCTCTTTAGACATGTCTTTGAACCCAATTGACTTCATTCAAGACCAAGCCTTTCAGGGAATTAAGCTCCATGAACTGACTCTAAGAGGTAATTTTAATAGCTCAAATATAATGAAAACTTGCCTTCAAAACCTGGCTGGTTTACACGTCCATCGGTTGATCTTGGGAGAATTTAAAGATGAAAGGAATCTGGAAATTTTTGAACCCTCTATCATGGAAGGACTATGTGATGTGACCATTGATGAGTTCAGGTTAACATATACAAATGATTTTTCAGATGATATTGTTAAGTTCCATTGCTTGGCGAATGTTTCTGCAATGTCTCTGGCAGGTGTATCTATAAAATATCTAGAAGATGTTCCTAAACATTTCAAATGGCAATCCTTATCAATCATTAGATGTCAACTTAAGCAGTTTCCAACTCTGGATCTACCCTTTCTTAAAAGTTTGACTTTAACTATGAACAAAGGGTCTATCAGTTTTAAAAAAGTGGCCCTACCAAGTCTCAGCTATCTAGATCTTAGTAGAAATGCACTGAGCTTTAGTGGTTGCTGTTCTTATTCTGATTTGGGAACAAACCCAAAGAGTCTAGCCTTCTTCAATCTTACTAACAATTCTGTTGCTTGTATATGTGAACATCAGAAATTCCTGCAGTGGGTCAAGGAACAGAAGCAGTTCTTGGTGAATGTTGAACAAATGACATGTGCAACACCTGTAGAGATGAATACCTCCTTAGTGTTGGATTTTAATAATTCTACCTGTTATATGTACAAGACAATCATCAGTGTGTCAGTGGTCAGTGTGATTGTGGTATCCACTGTAGCATTTCTGATATACCACTTCTATTTTCACCTGATACTTATTGCTGGCTGTAAAAAGTACAGCAGAGGAGAAAGCATCTATGATGCATTTGTGATCTACTCGAGTCAGAATGAGGACTGGGTGAGAAATGAGCTGGTAAAGAATTTAGAAGAAGGAGTGCCCCGCTTTCACCTCTGCCTTCACTACAGAGACTTTATTCCTGGTGTAGCCATTGCTGCCAACATCATCCAGGAAGGCTTCCACAAGAGCCGGAAGGTTATTGTGGTAGTGTCTAGACACTTTATTCAGAGCCGTTGGTGTATCTTTGAATATGAGATTGCTCAAACATGGCAGTTTCTGAGCAGCCGCTCTGGCATCATCTTCATTGTCCTTGAGAAGGTTGAGAAGTCCCTGCTGAGGCAGCAGGTGGAATTGTATCGCCTTCTTAGCAGAAACACCTACCTGGAATGGGAGGACAATCCTCTGGGGAGGCACATCTTCTGGAGAAGACTTAAAAATGCCCTATTGGATGGAAAAGCCTCGAATCCTGAGCAAACAGCAGAGGAAGAACAAGAAACGGCAACTTGGACCGATTACAAGGATGACGACGATAAGTGAGAATTCTGCAGATATCCAGCACAGTGGCGGCCGCTCGAGTCTAGAGGGCCCGTTTAAACCCGCTGATCAGCCTCGACTGTGCCTTCTAGTTGCCAGCCATCTGTTGTTTGCCCCTCCCCCGTGCCTTCCTTGACCCTGGAAGGTGCCACTCCCACTGTCCTTTCCTAATAAAATGAGGAAATTGCATCGCATTGTCTGAGTAGGTGTCATTCTATTCTGGGGGGTGGGGTGGGGCAGGACAGCAAGGGGGAGGATTGGGAAGACAATAGCAGGCATGCTGGGGATGCGGTGGGCTCTATGGCTTCTGAGGCGGAAAGAACCAGCTGGGGCTCTAGGGG

mTLr4-Flag（Δ576-627aa）CCATTGACGTCAATAATGACGTATGTTCCCATAGTAACGCCAATAGGGACTTTCCATTGACGTCAATGGGTGGAGTATTTACGGTAAACTGCCCACTTGGCAGTACATCAAGTGTATCATATGCCAAGTACGCCCCCTATTGACGTCAATGACGGTAAATGGCCCGCCTGGCATTATGCCCAGTACATGACCTTATGGGACTTTCCTACTTGGCAGTACATCTACGTATTAGTCATCGCTATTACCATGGTGATGCGGTTTTGGCAGTACATCAATGGGCGTGGATAGCGGTTTGACTCACGGGGATTTCCAAGTCTCCACCCCATTGACGTCAATGGGAGTTTGTTTTGGCACCAAAATCAACGGGACTTTCCAAAATGTCGTAACAACTCCGCCCCATTGACGCAAATGGGCGGTAGGCGTGTACGGTGGGAGGTCTATATAAGCAGAGCTCTCTGGCTAACTAGAGAACCCACTGCTTACTGGCTTATCGAAATTAATACGACTCACTATAGGGAGACCCAAGCTGGCTAGCGTTTAAACTTAAGCTTGGTACCGAGCTCGGATCCGCCACCATGATGCCTCCCTGGCTCCTGGCTAGGACTCTGATCATGGCACTGTTCTTCTCCTGCCTGACACCAGGAAGCTTGAATCCCTGCATAGAGGTAGTTCCTAATATTACCTACCAATGCATGGATCAGAAACTCAGCAAAGTCCCTGATGACATTCCTTCTTCAACCAAGAACATAGATCTGAGCTTCAACCCCTTGAAGATCTTAAAAAGCTATAGCTTCTCCAATTTTTCAGAACTTCAGTGGCTGGATTTATCCAGGTGTGAAATTGAAACAATTGAAGACAAGGCATGGCATGGCTTACACCACCTCTCAAACTTGATACTGACAGGAAACCCTATCCAGAGTTTTTCCCCAGGAAGTTTCTCTGGACTAACAAGTTTAGAGAATCTGGTGGCTGTGGAGACAAAATTGGCCTCTCTAGAAAGCTTCCCTATTGGACAGCTTATAACCTTAAAGAAACTCAATGTGGCTCACAATTTTATACATTCCTGTAAGTTACCTGCATATTTTTCCAATCTGACGAACCTAGTACATGTGGATCTTTCTTATAACTATATTCAAACTATTACTGTCAACGACTTACAGTTTCTACGTGAAAATCCACAAGTCAATCTCTCTTTAGACATGTCTTTGAACCCAATTGACTTCATTCAAGACCAAGCCTTTCAGGGAATTAAGCTCCATGAACTGACTCTAAGAGGTAATTTTAATAGCTCAAATATAATGAAAACTTGCCTTCAAAACCTGGCTGGTTTACACGTCCATCGGTTGATCTTGGGAGAATTTAAAGATGAAAGGAATCTGGAAATTTTTGAACCCTCTATCATGGAAGGACTATGTGATGTGACCATTGATGAGTTCAGGTTAACATATACAAATGATTTTTCAGATGATATTGTTAAGTTCCATTGCTTGGCGAATGTTTCTGCAATGTCTCTGGCAGGTGTATCTATAAAATATCTAGAAGATGTTCCTAAACATTTCAAATGGCAATCCTTATCAATCATTAGATGTCAACTTAAGCAGTTTCCAACTCTGGATCTACCCTTTCTTAAAAGTTTGACTTTAACTATGAACAAAGGGTCTATCAGTTTTAAAAAAGTGGCCCTACCAAGTCTCAGCTATCTAGATCTTAGTAGAAATGCACTGAGCTTTAGTGGTTGCTGTTCTTATTCTGATTTGGGAACAAACAGCCTGAGACACTTAGACCTCAGCTTCAATGGTGCCATCATTATGAGTGCCAATTTCATGGGTCTAGAAGAGCTGCAGCACCTGGATTTTCAGCACTCTACTTTAAAAAGGGTCACAGAATTCTCAGCGTTCTTATCCCTTGAAAAGCTACTTTACCTTGACATCTCTTATACTAACACCAAAATTGACTTCGATGGTATATTTCTTGGCTTGACCAGTCTCAACACATTAAAAATGGCTGGCAATTCTTTCAAAGACAACACCCTTTCAAATGTCTTTGCAAACACAACAAACTTGACATTCCTGGATCTTTCTAAATGTCAATTGGAACAAATATCTTGGGGGGTATTTGACACCCTCCATAGACTTCAATTATTAAATATGAGTCACAACAATCTATTGTTTTTGGATTCATCCCATTATAACCAGCTGTATTCCCTCAGCACTCTTGATTGCAGTTTCAATCGCATAGAGACATCTAAAGGAATACTGCAACATTTTCCAAAGAGTCTAGCCTTCTTCAATCTTACTAACTACAAGACAATCATCAGTGTGTCAGTGGTCAGTGTGATTGTGGTATCCACTGTAGCATTTCTGATATACCACTTCTATTTTCACCTGATACTTATTGCTGGCTGTAAAAAGTACAGCAGAGGAGAAAGCATCTATGATGCATTTGTGATCTACTCGAGTCAGAATGAGGACTGGGTGAGAAATGAGCTGGTAAAGAATTTAGAAGAAGGAGTGCCCCGCTTTCACCTCTGCCTTCACTACAGAGACTTTATTCCTGGTGTAGCCATTGCTGCCAACATCATCCAGGAAGGCTTCCACAAGAGCCGGAAGGTTATTGTGGTAGTGTCTAGACACTTTATTCAGAGCCGTTGGTGTATCTTTGAATATGAGATTGCTCAAACATGGCAGTTTCTGAGCAGCCGCTCTGGCATCATCTTCATTGTCCTTGAGAAGGTTGAGAAGTCCCTGCTGAGGCAGCAGGTGGAATTGTATCGCCTTCTTAGCAGAAACACCTACCTGGAATGGGAGGACAATCCTCTGGGGAGGCACATCTTCTGGAGAAGACTTAAAAATGCCCTATTGGATGGAAAAGCCTCGAATCCTGAGCAAACAGCAGAGGAAGAACAAGAAACGGCAACTTGGACCGATTACAAGGATGACGACGATAAGTGAGAATTCTGCAGATATCCAGCACAGTGGCGGCCGCTCGAGTCTAGAGGGCCCGTTTAAACCCGCTGATCAGCCTCGACTGTGCCTTCTAGTTGCCAGCCATCTGTTGTTTGCCCCTCCCCCGTGCCTTCCTTGACCCTGGAAGGTGCCACTCCCACTGTCCTTTCCTAATAAAATGAGGAAATTGCATCGCATTGTCTGAGTAGGTGTCATTCTATTCTGGGGGGTGGGGTGGGGCAGGACAGCAAGGGGGAGGATTGGGAAGACAATAGCAGGCATGCTGGGGATGCGGTGGGCTCTATGGCTTCTGAGGCGGAAAGAACCAGCTGGGGCTCTAGGGGGTATCCCCACGCGCCCTGTAGCGGCGCATTAAGCGCGGCGGGTGTGGTGGTTACGCGCAGCGTGACCGCTACACTTGCCAGCGCCCTAGCGCCCGCTCCTTTCGCTTTCTTCCCTTCCTTTCTCGCCACGTTCGCCGGCTTTCCCCGTCAAGCTCTAAATCGGGGGCTCCCTTTAGGGTTCCGATTTAGTGCTTTACGGCACCTCGACCCCAAAAAACTTGATTAGGGTGATGGTTCACGTAGTGGGCCATCGCCCTGATAGACGGTTTTTCGCCCTTTGACGTTGGAGTCCACGTTCTTTAATAGTGG

mTLr4-Flag（Δ670-835aa）AGTACATGACCTTATGGGACTTTCCTACTTGGCAGTACATCTACGTATTAGTCATCGCTATTACCATGGTGATGCGGTTTTGGCAGTACATCAATGGGCGTGGATAGCGGTTTGACTCACGGGGATTTCCAAGTCTCCACCCCATTGACGTCAATGGGAGTTTGTTTTGGCACCAAAATCAACGGGACTTTCCAAAATGTCGTAACAACTCCGCCCCATTGACGCAAATGGGCGGTAGGCGTGTACGGTGGGAGGTCTATATAAGCAGAGCTCTCTGGCTAACTAGAGAACCCACTGCTTACTGGCTTATCGAAATTAATACGACTCACTATAGGGAGACCCAAGCTGGCTAGCGTTTAAACTTAAGCTTGGTACCGAGCTCGGATCCGCCACCATGATGCCTCCCTGGCTCCTGGCTAGGACTCTGATCATGGCACTGTTCTTCTCCTGCCTGACACCAGGAAGCTTGAATCCCTGCATAGAGGTAGTTCCTAATATTACCTACCAATGCATGGATCAGAAACTCAGCAAAGTCCCTGATGACATTCCTTCTTCAACCAAGAACATAGATCTGAGCTTCAACCCCTTGAAGATCTTAAAAAGCTATAGCTTCTCCAATTTTTCAGAACTTCAGTGGCTGGATTTATCCAGGTGTGAAATTGAAACAATTGAAGACAAGGCATGGCATGGCTTACACCACCTCTCAAACTTGATACTGACAGGAAACCCTATCCAGAGTTTTTCCCCAGGAAGTTTCTCTGGACTAACAAGTTTAGAGAATCTGGTGGCTGTGGAGACAAAATTGGCCTCTCTAGAAAGCTTCCCTATTGGACAGCTTATAACCTTAAAGAAACTCAATGTGGCTCACAATTTTATACATTCCTGTAAGTTACCTGCATATTTTTCCAATCTGACGAACCTAGTACATGTGGATCTTTCTTATAACTATATTCAAACTATTACTGTCAACGACTTACAGTTTCTACGTGAAAATCCACAAGTCAATCTCTCTTTAGACATGTCTTTGAACCCAATTGACTTCATTCAAGACCAAGCCTTTCAGGGAATTAAGCTCCATGAACTGACTCTAAGAGGTAATTTTAATAGCTCAAATATAATGAAAACTTGCCTTCAAAACCTGGCTGGTTTACACGTCCATCGGTTGATCTTGGGAGAATTTAAAGATGAAAGGAATCTGGAAATTTTTGAACCCTCTATCATGGAAGGACTATGTGATGTGACCATTGATGAGTTCAGGTTAACATATACAAATGATTTTTCAGATGATATTGTTAAGTTCCATTGCTTGGCGAATGTTTCTGCAATGTCTCTGGCAGGTGTATCTATAAAATATCTAGAAGATGTTCCTAAACATTTCAAATGGCAATCCTTATCAATCATTAGATGTCAACTTAAGCAGTTTCCAACTCTGGATCTACCCTTTCTTAAAAGTTTGACTTTAACTATGAACAAAGGGTCTATCAGTTTTAAAAAAGTGGCCCTACCAAGTCTCAGCTATCTAGATCTTAGTAGAAATGCACTGAGCTTTAGTGGTTGCTGTTCTTATTCTGATTTGGGAACAAACAGCCTGAGACACTTAGACCTCAGCTTCAATGGTGCCATCATTATGAGTGCCAATTTCATGGGTCTAGAAGAGCTGCAGCACCTGGATTTTCAGCACTCTACTTTAAAAAGGGTCACAGAATTCTCAGCGTTCTTATCCCTTGAAAAGCTACTTTACCTTGACATCTCTTATACTAACACCAAAATTGACTTCGATGGTATATTTCTTGGCTTGACCAGTCTCAACACATTAAAAATGGCTGGCAATTCTTTCAAAGACAACACCCTTTCAAATGTCTTTGCAAACACAACAAACTTGACATTCCTGGATCTTTCTAAATGTCAATTGGAACAAATATCTTGGGGGGTATTTGACACCCTCCATAGACTTCAATTATTAAATATGAGTCACAACAATCTATTGTTTTTGGATTCATCCCATTATAACCAGCTGTATTCCCTCAGCACTCTTGATTGCAGTTTCAATCGCATAGAGACATCTAAAGGAATACTGCAACATTTTCCAAAGAGTCTAGCCTTCTTCAATCTTACTAACAATTCTGTTGCTTGTATATGTGAACATCAGAAATTCCTGCAGTGGGTCAAGGAACAGAAGCAGTTCTTGGTGAATGTTGAACAAATGACATGTGCAACACCTGTAGAGATGAATACCTCCTTAGTGTTGGATTTTAATAATTCTACCTGTTATATGTACAAGACAATCATCAGTGTGTCAGTGGTCAGTGTGATTGTGGTATCCACTGTAGCATTTCTGATATACCACTTCTATTTTCACCTGATACTTATTGCTGGCTGTAAAAAGTACAGCAGAGGAGAAGATTACAAGGATGACGACGATAAGTGAGAATTCTGCAGATATCCAGCACAGTGGCGGCCGCTCGAGTCTAGAGGGCCCGTTTAAACCCGCTGATCAGCCTCGACTGTGCCTTCTAGTTGCCAGCCATCTGTTGTTTGCCCCTCCCCCGTGCCTTCCTTGACCCTGGAAGGTGCCACTCCCACTGTCCTTTCCTAATAAAATGAGGAAATTGCATCGCATTGTCTGAGTAGGTGTCATTCTATTCTGGGGGGTGGGGTGGGGCAGGACAGCAAGGGGGAGGATTGGGAAGACAATAGCAGGCATGCTGGGGATGCGGTGGGCTCTATGGCTTCTGAGGCGGAAAGAACCAGCTGGGGCTCTAGGGGGTATCCCCACGCGCCCTGTAGCGGCGCATTAAGCGCGGCGGGTGTGGTGGTTACGCGCAGCGTGACCGCTACACTTGCCAGCGCCCTAGCGCCCGCTCCTTTCGCTTTCTTCCCTTCCTTTCTCGCCACGTTCGCCGGCTTTCCCCGTCAA
